# Prediction of the reliability of genomic breeding values for crossbred performance

**DOI:** 10.1186/s12711-017-0318-1

**Published:** 2017-05-12

**Authors:** Jérémie Vandenplas, Jack J. Windig, Mario P. L. Calus

**Affiliations:** Animal Breeding and Genomics Centre, Wageningen UR Livestock Research, P.O. Box 338, 6700 AH Wageningen, The Netherlands

## Abstract

**Background:**

In crossbreeding programs, various genomic prediction models have been proposed for using phenotypic records of crossbred animals to increase the selection response for crossbred performance in purebred animals. A possible model is a model that assumes identical single nucleotide polymorphism (SNP) effects for the crossbred performance trait across breeds (ASGM). Another model is a genomic model that assumes breed-specific effects of SNP alleles (BSAM) for crossbred performance. The aim of this study was to derive and validate equations for predicting the reliability of estimated genomic breeding values for crossbred performance in both these models. Prediction equations were derived for situations when all (phenotyping and) genotyping data have already been collected, i.e. based on the genetic evaluation model, and for situations when all genotyping data are not yet available, i.e. when designing breeding programs.

**Results:**

When all genotyping data are available, prediction equations are based on selection index theory. Without availability of all genotyping data, prediction equations are based on population parameters (e.g., heritability of the traits involved, genetic correlation between purebred and crossbred performance, effective number of chromosome segments). Validation of the equations for predicting the reliability of genomic breeding values without all genotyping data was performed based on simulated data of a two-way crossbreeding program, using either two closely-related breeds, or two unrelated breeds, to produce crossbred animals. The proposed equations can be used for an easy comparison of the reliability of genomic estimated breeding values across many scenarios, especially if all genotyping data are available. We show that BSAM outperforms ASGM for a specific breed, if the effective number of chromosome segments that originate from this breed and are shared by selection candidates of this breed and crossbred reference animals is less than half the effective number of all chromosome segments that are independently segregating in the same animals.

**Conclusions:**

The derived equations can be used to predict the reliability of genomic estimated breeding values for crossbred performance using ASGM or BSAM in many scenarios, and are thus useful to optimize the design of breeding programs. Scenarios can vary in terms of the genetic correlation between purebred and crossbred performances, heritabilities, number of reference animals, or distance between breeds.

**Electronic supplementary material:**

The online version of this article (doi:10.1186/s12711-017-0318-1) contains supplementary material, which is available to authorized users.

## Background

Several livestock production systems are based on crossbreeding schemes (e.g., [1–3]), and take advantage of the increased performance of crossbred animals compared to purebred animals, along with breed complementarity. For such production systems based on crossbreeding, the breeding goal for the purebred populations is to optimize the performance of crossbred descendants. However, the selection of purebred animals for crossbred performance has not been extensively implemented in livestock, partly due to the difficulty of routine collection of pedigree information on crossbred animals [4].

With the advent of genomic selection, various genomic prediction models have been proposed, which use phenotypic records of crossbred animals to increase the selection response for crossbred performance in purebred animals (e.g., [2, 4–6]). These approaches predict breeding values for crossbred performance of selection candidates using the estimated allele substitution effects of many single nucleotide polymorphisms (SNPs). The SNP allele substitution effects are estimated from phenotypes of genotyped reference animals. In the context of crossbreeding, several breeds and their crosses are involved in genomic prediction, and purebred and crossbred performances are often considered to be different but correlated traits (e.g., [1, 3, 5, 7, 8]). Therefore, estimates of SNP allele substitution effects for purebred and crossbred performance traits may not be the same for purebred and crossbred populations, e.g., due to genotype by environment interactions. Assuming only additive gene action, one approach to accommodate this is to model differences between allele substitution SNP effects using a multivariate genomic model that assumes a correlation structure between the effects of SNPs across the purebred and crossbred populations, or equivalently, by assuming a genetic correlation structure across the trait measured in purebred and crossbred populations [9, 10]. These multivariate genomic models are referred to hereafter as across-breed SNP genotype models (ASGM), since the estimates of SNP allele substitution effects for the crossbred performance trait are also used to predict breeding values for crossbred performance of purebred selection candidates, regardless of their breed of origin [4, 6]. Thus, estimates of SNP effects for the crossbred performance trait using ASGM are not breed-specific. However, a number of factors may have an impact on the effect that can be measured for a SNP for the crossbred performance trait. First, the two parental alleles at a SNP in a crossbred animal may have different effects on the phenotype due to different levels of linkage disequilibrium (LD) with a quantitative trait locus (QTL) in the parental purebred populations. Second, different genetic backgrounds, such as dominance or epistatic interactions, can result in the effects of the same QTL to be different in purebred versus crossbred animals. And third, purebred and crossbred animals may be exposed to different environments, leading to genotype by environment interactions. Because of these reasons, estimated allele substitution effects at SNPs for the crossbred performance trait may be breed-specific. To accommodate all these differences, previously an approach was proposed [3–6] that estimates breed-specific allele substitution effects for the crossbred performance trait (BSAM), assuming that the breed origin of SNP alleles in crossbred animals is known. Results from simulations have shown that BSAM can result in greater accuracy of genomic estimated breeding values (EBV) of purebreds for crossbred performance than ASGM under some conditions [2, 4, 11].

In order to be able to evaluate many different breeding program designs that apply genomic prediction for crossbreeding performance, it would be useful to be able to predict the reliability of genomic EBV using, for example, different genomic models or different breeding schemes. Prediction of reliability should preferably consider the genotype data of all reference animals and selection candidates when available, although it is also desirable to be able to predict the reliability when genotype data of, e.g., selection candidates is not available, i.e. when designing breeding programs. Various equations have been proposed in the literature to predict the reliability or the accuracy (i.e., the square root of reliability) of genomic EBV for (groups of) animals. The investigated genomic predictions rely on single-population genomic models [12, 13], and on ASGM [10, 14]. When genotypes are available for both reference animals and selection candidates, prediction equations are derived using selection index (SI) theory, while before availability of all genotyping data, they are derived using population parameters (e.g., heritability, number of reference animals) [10, 13, 15]. However, to our knowledge, equations for predicting the reliability of genomic EBV for crossbreeding performance for (groups of) animals have not yet been reported.

The primary aim of this study was to derive equations for predicting the reliability of genomic EBV for crossbred performance based on ASGM or BSAM. Prediction equations were derived for situations when all genotyping data are available for both reference animals and selection candidates (referred to as “with availability of genotyping data”), and for situations when the genotyping data are not available (referred to as “without availability of genotyping data”). The second aim was to compare the predictions of the reliability of genomic EBV without availability of genotyping data to the predictions obtained from the equations with availability of genotyping data, because the former are an approximation of the latter. Both reliabilities have the same expectation, since they both rely on prediction error variances (PEV) and assume absence of selection. Finally, the equation for predicting reliability without availability of genotyping data was used to investigate the expected ranges of reliabilities of genomic EBV using BSAM for a pig breeding program.

## Methods

The first part of this section describes equations for predicting the reliability of genomic EBV for crossbred performance using ASGM or BSAM. For the derivations of these equations, we assumed a crossbreeding program with two breeds, A and B, with their F1 being crossbred AB animals. In order to simplify the derivation of the equations, we assumed that phenotypes are corrected for all fixed and random effects, other than additive genetic effects. Furthermore, reference animals are defined as animals with genotypes and phenotypes, and selection candidates are defined as animals with genotypes but without their own phenotype. The assumption that all reference animals have genotypes is likely to be correct in the near future, as genotyping costs continue to decrease. The aim is to predict the reliability of genomic EBV for crossbred performance for selection candidates of breed A. For the reference population, three scenarios were investigated: (1) the reference population includes only breed A animals (PB–PB), i.e. purebred (PB) phenotypes are used to predict EBV for crossbred (CB) performance of PB selection candidates; (2) the reference population includes only crossbred AB animals (CB–PB), i.e. CB phenotypes are used to predict EBV for CB performance of PB selection candidates; and (iii) the reference population includes both crossbred AB and breed A animals (CB + PB–PB), i.e. CB and PB phenotypes are used to predict EBV for CB performance of PB selection candidates. These scenarios represent situations where crossbred animals are terminal animals in commercial herds of pigs and chickens. The second part of this section describes simulations of the three scenarios used to validate the prediction equations without availability of genotyping data. In the equations below, reference animals are indicated by uppercase letters, while selection candidates are indicated by lowercase letters.

### Across-breed SNP genotype models

Equations for predicting the reliability of genomic EBV for crossbred performance using ASGM were developed for the three scenarios. As ASGM is assumed, breed A and crossbred AB animals can be considered as belonging to different populations, assuming the genetic correlation between the PB and CB performance traits ($r_{PC}$) to be the genetic correlation between these breed A and crossbred AB populations. Therefore, equations for predicting the reliability of genomic EBV for crossbred performance for the three scenarios using ASGM can be derived from previous studies by, for example, Daetwyler et al. [12] and Wientjes et al. [10], without availability of genotyping data, and by VanRaden [15] with availability of genotyping data.

#### PB–PB scenario

The PB–PB scenario considers breed A animals for both reference animals and selection candidates. Phenotypes are therefore associated with the purebred performance trait, while the trait of interest is the crossbred performance trait. Indeed, selection candidates must be selected to optimize crossbred performance of their crossbred descendants.

Wientjes et al. [10] developed equations for predicting the accuracy of across-population genomic EBV values without and with availability of genotyping data. Assuming additive gene action, differences in allele substitution effects that underlie the population-specific trait of interest were modelled by the genetic correlation between traits, which implies a multivariate genomic model. Similarly, for the PB–PB scenario, differences in allele substitution effects that underlie the purebred and crossbred performance traits can be considered in terms of the genetic correlation between the purebred and crossbred performance traits ($r_{PC}$) [10, 16]. Therefore, following Wientjes et al. [10], with availability of genotyping data, the average predicted reliability of genomic EBV for crossbred performance across-breed A selection candidates using breed A reference animals can be computed as follows, based on SI theory:

1 $$r_{{P\_{{ASGM\_{with} }} }}^{2} = \frac{1}{{N_{a} }}\mathop \sum \limits_{i} r_{{P\_{{ASGM\_{with_{i}} }} }}^{2} = \frac{1}{{N_{a} }}\mathop \sum \limits_{i} r_{PC}^{2} \frac{{{\mathbf{G}}_{{a_{i} ,A}} \left( {{\mathbf{G}}_{AA} + {\mathbf{I}}\frac{{1 - h_{a}^{2} }}{{h_{a}^{2} }}} \right)^{ - 1} {\mathbf{G}}_{{A,a_{i} }} }}{{{\mathbf{G}}_{{a_{i} ,a_{i} }} }},$$

where $N_{a}$ is the number of breed A selection candidates; $h_{a}^{2} = \frac{{\sigma_{a}^{2} }}{{\sigma_{a}^{2} + \sigma_{{e_{A} }}^{2} }}$ is the heritability of the purebred performance trait, with $\sigma_{a}^{2}$ being the genetic variance of the purebred performance trait, and $\sigma_{{e_{A} }}^{2}$ the residual variance of purebred performance trait; $r_{PC} = \frac{{\sigma_{a,c} }}{{\sqrt {\sigma_{a}^{2} \sigma_{c}^{2} } }}$ with $\sigma_{a,c}$ being the genetic covariance between the purebred and crossbred performance trait and $\sigma_{c}^{2}$ the genetic variance of the crossbred performance trait; matrix ${\mathbf{G}}_{A,A}$ is the $N_{A} \times N_{A}$ genomic relationship matrix for the $N_{A}$ reference animals of breed A; vector ${\mathbf{G}}_{{a_{i} ,A}}$ is the row corresponding to the *i*th selection candidate of breed A of the $N_{a} \times N_{A}$ genomic relationship matrix ${\mathbf{G}}_{a,A}$ between selection candidates of breed A and reference animals of breed A; ${\mathbf{G}}_{{a_{i} ,a_{i} }}$ is the diagonal element corresponding to the *i*th selection candidate of breed A of the $N_{a} \times N_{a}$ genomic relationship matrix ${\mathbf{G}}_{a,a}$ between selection candidates of breed A; and matrix ${\mathbf{I}}$ is the identity matrix.

Matrices ${\mathbf{G}}_{A,A}$, ${\mathbf{G}}_{a,a}$, and ${\mathbf{G}}_{a,A}$ are parts of the genomic relationship matrix among all reference animals and selection candidates of breed A, i.e. $${\mathbf{G}} = \left[ {\begin{array}{*{20}c} {{\mathbf{G}}_{A,A} } & {\quad {\mathbf{G}}_{A,a} } \\ {{\mathbf{G}}_{a,A} } & {\quad {\mathbf{G}}_{a,a} } \\ \end{array} } \right]$$. Without loss of generality, and similar to Wientjes et al. [10], matrix ${\mathbf{G}}$ is computed following the second method of VanRaden [15], i.e., ${\mathbf{G}} = \frac{{{\mathbf{ZZ}}^{\prime } }}{m}$ where *m* is the number of SNP genotypes, and matrix ${\mathbf{Z}}$ contains the standardized genotypes as ${\mathbf{Z}}_{lk} = \frac{{{\mathbf{M}}_{lk} - 2p_{k} }}{{\sqrt {2p_{k} \left( {1 - p_{k} } \right)} }}$, with ${\mathbf{M}}_{lk}$ being the SNP genotype (coded as 0 for one homozygous genotype, 1 for the heterozygous genotype, or 2 for the alternate homozygous genotype) of the *l*th animal of breed A for the *k*th locus, and $p_{k}$ is the allele frequency at the *k*th locus.

Without availability of genotyping data, the predicted reliability of genomic EBV for crossbred performance of breed A selection candidates and using breed A reference animals can be computed as [10]:

2 $$r_{P\_ASGM\_without}^{2} = r_{PC}^{2} \frac{{N_{A} h_{a}^{2} }}{{N_{A} h_{a}^{2} + Me_{a,A} }},$$

where $Me_{a,A}$ is the effective number of chromosome segments that are shared between selection candidates and reference animals of breed A. If the term $r_{PC}^{2}$ is ignored, or equal to 1, Eq. (2) has the same form as the equation proposed by Daetwyler et al. [12]. One of the assumptions in the derivation of this equation was that the error variance was approximately equal to the phenotypic variance, because only one locus was taken into account at a time and each locus explains only a small part of the additive genetic variance [10, 12, 14]. However, as explained by Daetwyler et al. [12] in the Appendix of their paper, this approximation results in slight underestimation of the predicted reliabilities, because the error variance decreases when multiple loci are used. In Additional file 1 of the current study, we proposed a derivation of Eq. (2) based on the mixed model theory and ignoring the term $r_{PC}^{2}$. We assumed that a single population was used and that effects of all independent loci are estimated simultaneously. Our derivation leads to the same equation as proposed by Daetwyler et al. [12] in the Appendix of their paper, which corrects for the fact that the error variance decreases when multiple loci are used. This derivation using the mixed model theory can be extended for deriving prediction equations using ASGM, and it will be also the basis for deriving prediction equations using BSAM.

#### CB–PB scenario

The reference population for the CB–PB scenario includes genotyped crossbred AB animals that have phenotypes for the crossbred performance trait. The selection candidates are breed A animals that are related to the reference population and that must be selected to optimize crossbred performance of their crossbred AB descendants. Because the trait of interest is the crossbred performance trait and because allele substitution SNP effects are estimated from crossbred data, the average predicted reliability of genomic EBV for crossbred performance across breed A selection candidates using a crossbred AB reference population can be computed with availability of genotyping data as follows [10]:

3 $$r_{{C\_{{ASGM\_{with} }} }}^{2} = \frac{1}{{N_{a} }}\mathop \sum \limits_{i} r_{{C\_{{ASGM\_{{with_{i} }} }} }}^{2} = \frac{1}{{N_{a} }}\mathop \sum \limits_{i} \frac{{{\mathbf{G}}_{{a_{i} ,AB}} \left( {{\mathbf{G}}_{AB,AB} + {\mathbf{I}}\frac{{1 - h_{c}^{2} }}{{h_{c}^{2} }}} \right)^{ - 1} {\mathbf{G}}_{{AB,a_{i} }} }}{{{\mathbf{G}}_{{a_{i} ,a_{i} }} }}$$

where $h_{c}^{2} = \frac{{\sigma_{c}^{2} }}{{\sigma_{c}^{2} + \sigma_{{e_{c} }}^{2} }}$ is the heritability of the crossbred performance trait, with $\sigma_{{e_{c} }}^{2}$ being the residual variance; matrix ${\mathbf{G}}_{AB,AB}$ is the $N_{AB} \times N_{AB}$ genomic relationship matrix between the $N_{AB}$ crossbred AB reference animals; and vector ${\mathbf{G}}_{{a_{i} ,AB}}$ is the row corresponding to the *i*th selection candidate of breed A of the $N_{a} \times N_{AB}$ genomic relationship matrix ${\mathbf{G}}_{aAB}$ between breed A selection candidates and crossbred AB reference animals. Similarly to Wientjes et al. [10], the genomic relationship matrix between breed A selection candidates and crossbred AB reference animals, ${\mathbf{G}}$, is computed following the second method of VanRaden [15] but taking into account that the selection candidates and reference animals belong to two different populations. It then follows that $${\mathbf{G}} = \left[ {\begin{array}{*{20}c} {{\mathbf{G}}_{AB,AB} } & {\quad {\mathbf{G}}_{AB,a} } \\ {{\mathbf{G}}_{a,AB} } & {\quad {\mathbf{G}}_{a,a} } \\ \end{array} } \right] = \frac{{{\mathbf{ZZ}}^{\prime } }}{m}$$, where $m$ is the number of SNPs and matrix ${\mathbf{Z}}$ contains the standardized genotypes as ${\mathbf{Z}}_{ljk} = \frac{{{\mathbf{M}}_{ljk} - 2p_{jk} }}{{\sqrt {2p_{jk} \left( {1 - p_{jk} } \right)} }}$, with ${\mathbf{M}}_{ljk}$ being the SNP genotype (coded as previously) of the *l*th individual from the *j*th population (i.e., purebred or crossbred) for the *k*th locus, and $p_{jk}$ is the allele frequency of the *j*th population at the *k*th locus.

Without availability of data, an equation that predicts the reliability of genomic EBV for crossbred performance of breed A selection candidates using $N_{AB}$ crossbred AB reference animals can be simply written as follows:

4 $$r_{C\_ASGM\_without}^{2} = \frac{{N_{AB} h_{c}^{2} }}{{N_{AB} h_{c}^{2} + Me_{a,AB} }},$$

where $Me_{a,AB}$ is the effective number of chromosome segments shared by breed A selection candidates and crossbred AB reference animals [10].

#### CB + PB–PB scenario

The reference population for the CB + PB–PB scenario includes animals of breed A with phenotypes for the purebred performance trait and crossbred AB animals with phenotypes for the crossbred performance trait. The selection candidates are animals of breed A that are related to the reference population. Since the crossbred performance trait is the trait of interest, the average predicted reliability of genomic EBV across selection candidates for crossbred performance of breed A selection candidates using breed A and crossbred AB reference animals can be computed with availability of genotyping data as follows [10]:

5 $$r_{C+P{\_}ASGM{\_}with }^{2} = \frac{1}{{N_{a} }}\mathop \sum \limits_{i} r_{{C + P\_ASGM\_with_{i} }}^{2} = \frac{1}{{N_{a} }}\mathop \sum \limits_{i} \frac{1}{{{\mathbf{G}}_{{a_{i} ,a_{i} }} }}\left[ {\begin{array}{*{20}c} {r_{PC} {\mathbf{G}}_{{a_{i} ,A}} } & {\quad {\mathbf{G}}_{{a_{i} ,AB}} } \\ \end{array} } \right] \times \left[ {\begin{array}{*{20}c} {{\mathbf{G}}_{A,A} + {\mathbf{I}}\frac{{1 - h_{a}^{2} }}{{h_{a}^{2} }}} & {\quad {\mathbf{G}}_{A,AB} r_{PC} } \\ {r_{PC} {\mathbf{G}}_{AB,A} } & {\quad {\mathbf{G}}_{AB,AB} + {\mathbf{I}}\frac{{1 - h_{c}^{2} }}{{h_{c}^{2} }}} \\ \end{array} } \right]^{ - 1} \left[ {\begin{array}{*{20}c} {r_{PC} {\mathbf{G}}_{{a_{i} ,A}} } \\ {{\mathbf{G}}_{{a_{i} ,AB}} } \\ \end{array} } \right].$$

Without availability of genotyping data, the prediction equation for the reliability of genomic EBV for crossbred performance of breed A selection candidates using breed A and crossbred AB reference animals can be written as follows [14]:

6 $$r_{C + P\_ASGM\_without}^{2} = \left[ {\begin{array}{*{20}c} {r_{PC} \sqrt {\frac{{h_{a}^{2} }}{{Me_{a,A} }}} } & {\sqrt {\frac{{h_{c}^{2} }}{{Me_{a,AB} }}} } \\ \end{array} } \right]\left[ {\begin{array}{*{20}c} {\frac{{h_{a}^{2} }}{{Me_{a,A} }} + \frac{1}{{N_{A} }}} & {\quad r_{PC} \sqrt {\frac{{h_{a}^{2} h_{c}^{2} }}{{Me_{a,A} Me_{a,AB} }}} } \\ {r_{PC} \sqrt {\frac{{h_{a}^{2} h_{c}^{2} }}{{Me_{a,A} Me_{a,AB} }}} } & {\quad \frac{{h_{c}^{2} }}{{Me_{a,AB} }} + \frac{1}{{N_{AB} }}} \\ \end{array} } \right]^{ - 1} \left[ {\begin{array}{*{20}c} {r_{PC} \sqrt {\frac{{h_{a}^{2} }}{{Me_{a,A} }}} } \\ {\sqrt {\frac{{h_{c}^{2} }}{{Me_{a,AB} }}} } \\ \end{array} } \right].$$

### Breed-specific allele substitution models

In crossbred populations, SNP effects may be breed-specific due to a number of factors [4], including different extents of LD between SNP and QTL between breeds, which can be accommodated by using BSAM, which fits breed-specific allele substitution effects [3, 4]. In this section, it is assumed that the breed origin of SNP alleles is known, as required by BSAM. Moreover, only the CB–PB and CB + PB–PB scenarios are considered, since the PB–PB scenario involves data on only one breed. To our knowledge, equations for predicting the reliability of genomic EBV using BSAM have not previously been developed.

#### CB–PB scenario

For the CB–PB scenario, assuming that each individual has one phenotypic record corrected for all effects other than the additive genetic effects, BSAM for the crossbred performance trait can be written as follows [3, 4]:

$${\mathbf{y}}_{AB} = {\mathbf{Z}}_{AB}^{\left( A \right)} {\varvec{\upbeta}}_{c}^{\left( A \right)} + {\mathbf{Z}}_{AB}^{\left( B \right)} {\varvec{\upbeta}}_{c}^{\left( B \right)} + {\mathbf{e}}_{AB} ,$$

where ${\mathbf{y}}_{AB}$ is the vector of corrected records of crossbred performance; ${\mathbf{Z}}_{AB}^{\left( A \right)}$ (${\mathbf{Z}}_{AB}^{\left( B \right)}$) contains the standardized breed A (B) SNP alleles of each crossbred animal; ${\varvec{\upbeta}}_{c}^{\left( A \right)}$ (${\varvec{\upbeta}}_{c}^{\left( B \right)}$) is the vector of breed A (B)-specific allele substitution effects for all SNPs; and ${\mathbf{e}}_{\text{AB}}$ is the residual vector. Entries of matrix ${\mathbf{Z}}_{AB}^{\left( A \right)}$ are defined as ${\text{Z}}_{{AB_{lk} }}^{\left( A \right)} = \frac{{{\mathbf{M}}_{lk} - p_{Ak} }}{{\sqrt {2p_{Ak} \left( {1 - p_{Ak} } \right)} }}$, where element ${\text{M}}_{lk}$ is set to 0 or 1 when the *k*th locus of the *l*th individual has breed A allele 1 or 2, respectively; and $p_{Ak}$ is the frequency at the *k*th locus for breed A. Matrix ${\mathbf{Z}}_{AB}^{\left( B \right)}$ is defined similarly. Expectations and variances of ${\varvec{\upbeta}}_{c}^{\left( A \right)}$ and ${\varvec{\upbeta}}_{c}^{\left( B \right)}$ are assumed to be $$E\left[ {\begin{array}{*{20}c} {{\varvec{\upbeta}}_{c}^{\left( A \right)} } \\ {{\varvec{\upbeta}}_{c}^{\left( B \right)} } \\ \end{array} } \right] = \left[ {\begin{array}{*{20}c} {\mathbf{0}} \\ {\mathbf{0}} \\ \end{array} } \right]$$ and

$$Var\left[ {\begin{array}{*{20}c} {{\varvec{\upbeta}}_{c}^{\left( A \right)} } \\ {{\varvec{\upbeta}}_{c}^{\left( B \right)} } \\ \end{array} } \right] = \left[ {\begin{array}{*{20}c} {{\mathbf{I}}\sigma_{{\beta_{c}^{\left( A \right)} }}^{2} } & {\quad {\mathbf{0}}} \\ {\mathbf{0}} & {\quad {\mathbf{I}}\sigma_{{\beta_{c}^{\left( B \right)} }}^{2} } \\ \end{array} } \right] = \left[ {\begin{array}{*{20}c} {{\mathbf{I}}\frac{{\sigma_{{c_{A} }}^{2} }}{m}} & {\quad {\mathbf{0}}} \\ {\mathbf{0}} & {\quad {\mathbf{I}}\frac{{\sigma_{{c_{B} }}^{2} }}{m}} \\ \end{array} } \right]$$

where $\sigma_{{\beta_{c}^{\left( A \right)} }}^{2}$ ($\sigma_{{\beta_{c}^{\left( B \right)} }}^{2}$) is the variance of the breed A (B)-specific allele substitution effect, and $\sigma_{{c_{A} }}^{2}$ ($\sigma_{{c_{B} }}^{2}$) is the additive genetic variance due to alleles from population A (B) in the crossbred population for the crossbred performance trait [3, 4].

Equivalently, BSAM for the crossbred performance trait can be written as [3]:

$${\mathbf{y}}_{\text{AB}} = {\mathbf{c}}_{AB}^{\left( A \right)} + {\mathbf{c}}_{AB}^{\left( B \right)} + {\mathbf{e}}_{AB} ,$$

where ${\mathbf{c}}_{AB}^{\left( A \right)} = {\mathbf{Z}}_{AB}^{\left( A \right)} {\varvec{\upbeta}}_{c}^{\left( A \right)}$ (${\mathbf{c}}_{AB}^{\left( B \right)} = {\mathbf{Z}}_{AB}^{\left( B \right)} {\varvec{\upbeta}}_{c}^{\left( B \right)}$) is the vector of breed A (B) of origin additive genetic effects for the crossbred performance trait. It then follows that expectations and variances of ${\mathbf{c}}_{AB}^{\left( A \right)}$ and ${\mathbf{c}}_{AB}^{\left( B \right)}$ are defined as $$E\left[ {\begin{array}{*{20}c} {{\mathbf{c}}_{AB}^{\left( A \right)} } \\ {{\mathbf{c}}_{AB}^{\left( B \right)} } \\ \end{array} } \right] = \left[ {\begin{array}{*{20}c} {\mathbf{0}} \\ {\mathbf{0}} \\ \end{array} } \right]$$ and

$$Var\left[ {\begin{array}{*{20}c} {{\mathbf{c}}_{AB}^{\left( A \right)} } \\ {{\mathbf{c}}_{AB}^{\left( B \right)} } \\ \end{array} } \right] = \left[ {\begin{array}{*{20}c} {{\mathbf{Z}}_{AB}^{\left( A \right)} {\mathbf{Z}}_{AB}^{{\left( {\text{A}} \right)^{\prime } }} \frac{{\sigma_{{c_{A} }}^{2} }}{m}} & {\quad {\mathbf{0}}} \\ {\quad {\mathbf{0}}} & {{\mathbf{Z}}_{AB}^{\left( B \right)} {\mathbf{Z}}_{AB}^{{\left( B \right)^{\prime } }} {\mathbf{I}}\frac{{\sigma_{{c_{B} }}^{2} }}{m}} \\ \end{array} } \right] = \left[ {\begin{array}{*{20}c} {{\mathbf{G}}_{AB,AB}^{\left( A \right)} \sigma_{{c_{A} }}^{2} } & {\quad {\mathbf{0}}} \\ {\quad {\mathbf{0}}} & {{\mathbf{G}}_{AB,AB}^{\left( B \right)} \sigma_{{c_{B} }}^{2} } \\ \end{array} } \right],$$

where ${\mathbf{G}}_{AB,AB}^{\left( A \right)}$ (${\mathbf{G}}_{AB,AB}^{\left( B \right)}$) is the breed A (B) partial genomic relationship among the $N_{AB}$ crossbred AB animals [3]. These assumptions imply that ${\mathbf{c}}_{AB}^{\left( A \right)}$ and ${\mathbf{c}}_{AB}^{\left( B \right)}$, as well as ${\varvec{\upbeta}}_{c}^{\left( A \right)}$ and ${\varvec{\upbeta}}_{c}^{\left( B \right)}$, are independent of each other.

Based on SI theory, genomic EBV for crossbred performance of selection candidates from breed A (${\mathbf{c}}_{a}^{\left( A \right)}$) can be predicted from records of crossbred AB reference animals as follows:

$${\hat{\mathbf{c}}}_{a}^{\left( A \right)} = Cov\left( {{\mathbf{c}}_{a}^{\left( A \right)} ,{\mathbf{y}}_{AB} } \right)\left( {Var\left( {{\mathbf{y}}_{AB} } \right)} \right)^{ - 1} {\mathbf{y}}_{AB} .$$

Using the model description, it can then be shown that the variance of ${\mathbf{y}}_{\text{AB}}$ is equal to:

$$Var\left( {{\mathbf{y}}_{AB} } \right) = {\mathbf{G}}_{ABAB}^{\left( A \right)} \sigma_{{c_{A} }}^{2} + {\mathbf{G}}_{ABAB}^{\left( B \right)} \sigma_{{c_{B} }}^{2} + {\mathbf{I}}\sigma_{{e_{AB} }}^{2} ,$$

and that the covariance between ${\mathbf{c}}_{a}^{\left( A \right)}$ and ${\mathbf{y}}_{AB}$ is equal to:

$$Cov\left( {{\mathbf{c}}_{a}^{\left( A \right)} ,{\mathbf{y}}_{AB} } \right) = Cov\left( {{\mathbf{c}}_{a}^{\left( A \right)} ,{\mathbf{c}}_{AB}^{\left( A \right)} + {\mathbf{c}}_{AB}^{\left( B \right)} + {\mathbf{e}}_{AB} } \right) = Cov\left( {{\mathbf{c}}_{a}^{\left( A \right)} ,{\mathbf{c}}_{AB}^{\left( A \right)} } \right) + Cov\left( {{\mathbf{c}}_{a}^{\left( A \right)} ,{\mathbf{c}}_{AB}^{\left( B \right)} } \right) + Cov\left( {{\mathbf{c}}_{a}^{\left( A \right)} ,{\mathbf{e}}_{AB} } \right) = Cov\left( {{\mathbf{c}}_{a}^{\left( A \right)} ,{\mathbf{c}}_{AB}^{\left( A \right)} } \right) = {\mathbf{G}}_{a,AB}^{\left( A \right)} \sigma_{{c_{A} }}^{2} ,$$

with matrix ${\mathbf{G}}_{a,AB}^{\left( A \right)} = \frac{1}{m}{\mathbf{Z}}_{a}^{\left( A \right)} {\mathbf{Z}}_{AB}^{{\left( A \right)^{\prime } }}$ being the breed A-specific partial genomic relationship matrix between the $N_{a}$ selection candidates of breed A and the $N_{AB}$ crossbred AB reference animals.

The reliability of ${\hat{\text{c}}}_{{a_{i} }}^{\left( A \right)}$ of the *i*th selection candidate of breed A is then equal to:

$$r_{{C{\_}BSAM{\_}with_{i} }}^{2} = \frac{{\left( {Cov\left( {{\hat{\text{c}}}_{{{\text{a}}_{\text{i}} }}^{{\left( {\text{A}} \right)}} ,{\text{c}}_{{{\text{a}}_{\text{i}} }}^{{\left( {\text{A}} \right)}} } \right)} \right)^{2} }}{{Var\left( {{\hat{\text{c}}}_{{{\text{a}}_{\text{i}} }}^{{\left( {\text{A}} \right)}} } \right)Var\left( {{\text{c}}_{{{\text{a}}_{\text{i}} }}^{{\left( {\text{A}} \right)}} } \right)}} = \frac{{Var\left( {{\hat{\text{c}}}_{{{\text{a}}_{\text{i}} }}^{{\left( {\text{A}} \right)}} } \right)}}{{Var\left( {{\text{c}}_{{{\text{a}}_{\text{i}} }}^{{\left( {\text{A}} \right)}} } \right)}}= \frac{1}{{{\mathbf{G}}_{{a_{i} ,a_{i} }}^{{\left( A \right)}} }}{\mathbf{G}}_{{a_{i} ,AB}}^{{\left( A \right)}} \left( {{\mathbf{G}}_{{AB,AB}}^{{\left( A \right)}} + {\mathbf{G}}_{{AB,AB}}^{{\left( B \right)}} \frac{{{{\upsigma }}_{{{\text{c}}_{B} }}^{2} }}{{{{\upsigma }}_{{{\text{c}}_{A} }}^{2} }} + {\mathbf{I}}\frac{{{{\upsigma }}_{{\text{e}}}^{2} }}{{{{\upsigma }}_{{{\text{c}}_{A} }}^{2} }}} \right)^{{ - 1}} {\mathbf{G}}_{{AB,a_{i} }}^{{\left( A \right)}}.$$

With availability of genotyping data, the average predicted reliability of genomic EBV across all breed A selection candidates is equal to:

7 $$r_{C\_BSAM\_with}^{2} = \frac{1}{{N_{a} }}\mathop \sum \limits_{\text{i}} r_{{C\_BSAM\_with_{i} }}^{2} = \frac{1}{{N_{a} }}\mathop \sum \limits_{i} \frac{1}{{{\mathbf{G}}_{{a_{i} ,a_{i} }}^{\left( A \right)} }}{\mathbf{G}}_{{a_{i} ,AB}}^{\left( A \right)} \left( {{\mathbf{G}}_{AB,AB}^{\left( A \right)} + {\mathbf{G}}_{AB,AB}^{\left( B \right)} \frac{{h_{{c_{B} }}^{2} }}{{h_{{c_{A} }}^{2} }} + {\mathbf{I}}\frac{{1 - \frac{1}{2}h_{{c_{A} }}^{2} - \frac{1}{2}h_{{c_{B} }}^{2} }}{{h_{{c_{A} }}^{2} }}} \right)^{ - 1} {\mathbf{G}}_{{AB,a_{i} }}^{\left( A \right)} ,$$

where $h_{{c_{A} }}^{2} = \frac{{\sigma_{{c_{A} }}^{2} }}{{\frac{{\sigma_{{c_{A} }}^{2} }}{2} + \frac{{\sigma_{{c_{B} }}^{2} }}{2} + \sigma_{{e_{AB} }}^{2} }}\;\left( {h_{{c_{B} }}^{2} = \frac{{\sigma_{{c_{B} }}^{2} }}{{\frac{{\sigma_{{c_{A} }}^{2} }}{2} + \frac{{\sigma_{{c_{B} }}^{2} }}{2} + \sigma_{{e_{AB} }}^{2} }}} \right)$ is the breed A (B)-specific heritability of crossbred performance.

Since no equation has previously been proposed to predict the reliability of genomic EBV for BSAM without availability of genotyping data, here, we put forward a derivation based on mixed model theory [17], assuming that allele substitution effects for breeds A and B are estimated simultaneously. Equivalence between the mixed model and SI theories has previously been shown under certain conditions, including the use of the same estimates of the fixed effects [15, 17, 18]. Our derivation of the equation for predicting the reliability of genomic EBV for BSAM without availability of genotyping data [i.e., Eq. (8) below] is detailed in Additional file 2, and the result is briefly described in the following.

Consider $N_{AB}$ unrelated genotyped crossbred AB reference animals. For simplicity, it is assumed that the breed A-specific effect ${{\upbeta }}_{{c_{k} }}^{ *\left( A \right)}$ of each *k*th independent locus explains an equal amount of the breed A-specific additive genetic variance $\sigma_{{c_{A} }}^{2}$, i.e., $\sigma_{{c_{A} }}^{2} = Me_{a,AB}^{\left( A \right)} \sigma_{{\beta_{c}^{*\left( A \right)} }}^{2}$, with $Me_{a,AB}^{\left( A \right)}$ being the effective number of chromosome segments underlying the crossbred performance trait for breed A and segregating in both breed A selection candidates and crossbred AB reference animals. The same assumption is made for the breed B-specific effect ${{\upbeta }}_{{c_{k} }}^{ *\left( B \right)}$. The genomic EBV (${\text{c}}_{{a_{i} }}^{\left( A \right)}$) for the *i*th selection candidate of breed A can be predicted as follows:

$${\hat{\text{c}}}_{{a_{i} }}^{(A)} = {\mathbf{z}}_{{a_{i} }}^{*(A)} {\hat{\varvec{\upbeta }}}_{c}^{*(A)} ,$$

where ${\mathbf{z}}_{{a_{i} }}^{{{*}\left( A \right)}}$ is a vector of the standardized genotypes for the $Me_{a,AB}^{\left( A \right)}$ independent loci of the *i*th selection candidate of breed A and ${\hat{\varvec{\upbeta }}}_{c}^{*(A)}$ is the vector of the predictions of ${\varvec{\upbeta}}_{c}^{*\left( A \right)}$. Following mixed model theory [17, 19], the reliability of ${\hat{\text{c}}}_{{a_{i} }}^{\left( A \right)}$ can be computed from the prediction error variance, $Var\left( {{\hat{\text{c}}}_{{a_{i} }}^{\left( A \right)} - {\text{c}}_{{a_{i} }}^{\left( A \right)} } \right)$, and is equal to:

$$r_{{C\_{{BSAM\_{{without_{i} }} }} }}^{2} = 1 - \frac{{Var\left( {{\hat{\text{c}}}_{{a_{i} }}^{\left( A \right)} - {\text{c}}_{{a_{i} }}^{\left( A \right)} } \right)}}{{Var\left( {{\text{c}}_{{a_{i} }}^{\left( A \right)} } \right)}} = \frac{{Var\left( {{\hat{\text{c}}}_{{a_{i} }}^{\left( A \right)} } \right)}}{{Var\left( {{\text{c}}_{{a_{i} }}^{\left( A \right)} } \right)}} = \frac{{Var\left( {{\mathbf{z}}_{{a_{i} }}^{{{*}\left( A \right)}} \varvec{ }{\hat{\varvec{\upbeta }}}_{c}^{*\left( A \right)} } \right)}}{{Var\left( {{\mathbf{z}}_{{a_{i} }}^{{{*}\left( A \right)}} \varvec{ }{\varvec{\upbeta}}_{c}^{*\left( A \right)} } \right)}} = \frac{{{\mathbf{z}}_{{a_{i} }}^{{{*}\left( A \right)}} Var\left( {\varvec{ }{\hat{\varvec{\upbeta }}}_{c}^{*\left( A \right)} } \right){\mathbf{z}}_{{a_{i} }}^{{{*}\left( A \right)^{\prime} }} }}{{{\mathbf{z}}_{{a_{i} }}^{{{*}\left( A \right)}} Var\left( {\varvec{ }{\varvec{\upbeta}}_{c}^{*\left( A \right)} } \right){\mathbf{z}}_{{a_{i} }}^{{{*}\left( A \right)^{\prime} }} }}.$$

Assuming that the allele substitution effect ${{\upbeta }}_{{c_{k} }}^{ *\left( A \right)}$ of each *k*th independent locus explains an equal amount of the breed A-specific additive genetic variance $\sigma_{{c_{A} }}^{2}$ and that the reliability of the estimated effect, $r_{{\beta_{c}^{*\left( A \right)} }}^{2}$, is the same for each locus, it follows that:

$$r_{{C\_BSAM\_without_{i} }}^{2} = \frac{{Var\left( {{\hat{\upbeta }}_{{c_{k} }}^{*\left( A \right)} } \right)}}{{Var\left( {\upbeta_{{c_{k} }}^{*\left( A \right)} } \right)}} = r_{{\beta_{c}^{*\left( A \right)} }}^{2} .$$

Reliability $r_{{\beta_{c}^{*\left( A \right)} }}^{2}$ can be approximated as follows. Let $\widehat{{{\mathbf{y}}_{AB}^{*} }}$ be the vector of phenotypes corrected for all other fixed effects for the breed A-specific allele substitution effects other than the *k*th effect, ${\hat{\varvec{\upbeta }}}_{{c_{ \ne k} }}^{*\left( A \right)}$, as well as for the breed B-specific allele substitution effects, ${\hat{\varvec{\upbeta }}}_{c}^{ *\left( B \right)}$. The prediction of ${{\upbeta }}_{{c_{k} }}^{*\left( A \right)}$ for the *k*th locus can then be performed using the following model:

$$\widehat{{{\mathbf{y}}_{AB}^{*} }} = {\mathbf{z}}_{{AB_{k} }}^{{{*}\left( A \right)}} \upbeta_{{c_{k} }}^{*\left( A \right)} + {\varvec{\upvarepsilon}}_{{AB_{k} }} ,$$

where vector ${\mathbf{z}}_{{AB_{k} }}^{{{*}\left( A \right)}}$ contains the standardized breed A alleles of crossbred AB reference animals, and ${\varvec{\upvarepsilon}}_{{AB_{k} }}$ is the residual vector.

The variance of $\widehat{{{\mathbf{y}}_{AB}^{*} }}$ is equal to:

$$Var\left( {\widehat{{{\mathbf{y}}_{AB}^{*} }}} \right) = Var\left( {{\mathbf{z}}_{{AB_{k} }}^{{{*}\left( A \right)}} \upbeta_{{c_{k} }}^{*\left( A \right)} } \right) + Var\left( {{\varvec{\upvarepsilon}}_{{AB_{k} }} } \right) = {\mathbf{z}}_{{AB_{k} }}^{{{*}\left( A \right)}} {\mathbf{z}}_{{AB_{k} }}^{{{*}\left( A \right)^{\prime} }} \sigma_{{\beta_{c}^{*\left( A \right)} }}^{2} + Var\left( {{\varvec{\upvarepsilon}}_{{AB_{k} }} } \right),$$

from which it follows that, after some algebra and assuming unrelated genotyped animals (see Additional file 2 for details):

$$Var\left( {{\varvec{\upvarepsilon}}_{{AB_{k} }} } \right) = Var\left( {\widehat{{{\mathbf{y}}_{AB}^{*} }}} \right) - {\mathbf{z}}_{{AB_{k} }}^{{{*}\left( A \right)}} {\mathbf{z}}_{{AB_{k} }}^{{{*}\left( A \right)^{\prime} }} \sigma_{{\beta_{c}^{*\left( A \right)} }}^{2} = {\mathbf{I}}\left( {\frac{{\sigma_{{c_{A} }}^{2} }}{2}\left( {1 - r_{{\beta_{c}^{*\left( A \right)} }}^{2} } \right) + \frac{{\sigma_{{c_{B} }}^{2} }}{2}\left( {1 - r_{{\beta_{c}^{*\left( B \right)} }}^{2} } \right) + \sigma_{{e_{AB} }}^{2} } \right) \approx {\mathbf{I}}\left( {\sigma_{{P_{AB} }}^{2} - \frac{{\sigma_{{c_{A} }}^{2} }}{2}r_{{\beta_{c}^{*\left( A \right)} }}^{2} - \frac{{\sigma_{{c_{B} }}^{2} }}{2}r_{{\beta_{c}^{*\left( B \right)} }}^{2} } \right) = {\mathbf{I}}\sigma_{{\varepsilon_{AB} }}^{2} ,$$

where $\sigma_{{P_{AB} }}^{2}$ $\left( {\sigma_{{e_{AB} }}^{2} } \right)$ is the phenotypic (residual) variance of the crossbred performance trait.

Therefore, following mixed model theory [17, 19], the prediction of $\upbeta_{{c_{k} }}^{ *\left( A \right)} ,{\hat{\upbeta }}_{{c_{k} }}^{*\left( A \right)}$, is equal to:

$${\hat{{\upbeta }}}_{{c_{k} }}^{*\left( A \right)} = \left( {{\mathbf{z}}_{{AB_{k} }}^{{{*}\left( A \right)^{\prime} }} {\mathbf{z}}_{{AB_{k} }}^{{{*}\left( A \right)}} \sigma_{{\varepsilon_{AB} }}^{ - 2} + \sigma_{{\beta_{c}^{*\left( A \right)} }}^{ - 2} } \right)^{ - 1} \sigma_{{\varepsilon_{AB} }}^{ - 2} {\mathbf{z}}_{{AB_{k} }}^{{{*}\left( A \right)^{\prime} }} \widehat{{{\mathbf{y}}_{AB}^{*} }},$$

and the reliability of ${\hat{{\upbeta }}}_{{c_{k} }}^{*\left( A \right)}$ is equal to (see Additional file 2 for more details):

$$r_{{\beta_{c}^{*\left( A \right)} }}^{2} = \frac{{Var\left( {\upbeta_{{c_{k} }}^{ *\left( A \right)} } \right) - Var\left( {{\hat{\upbeta }}_{{c_{k} }}^{*\left( A \right)} - {{\upbeta }}_{{c_{k} }}^{ *\left( A \right)} } \right)}}{{Var\left( {\upbeta_{{c_{k} }}^{ *\left( A \right)} } \right)}} = \frac{{N_{AB} \sigma_{{\varepsilon_{AB} }}^{ - 2} \sigma_{{\beta_{c}^{*\left( A \right)} }}^{2} }}{{N_{AB} \sigma_{{\varepsilon_{AB} }}^{ - 2} \sigma_{{\beta_{c}^{*\left( A \right)} }}^{2} + 2}}.$$

It then follows that, without availability of genotyping data, the predicted reliability of the genomic EBV for breed A selection candidates is equal to (see Additional file 2 for more details):

$$r_{{C\_{{BSAM\_{without} }} }}^{2} = r_{{\beta_{c}^{*\left( A \right)} }}^{2} = \frac{{N_{AB} h_{{c_{A} }}^{2} }}{{N_{AB} h_{{c_{A} }}^{2} + 2Me_{a,AB}^{\left( A \right)} \left( {1 - \frac{1}{2}h_{{c_{A} }}^{2} r_{{c_{a}^{\left( A \right)} }}^{2} - \frac{1}{2}h_{{c_{B} }}^{2} r_{{c_{a}^{\left( B \right)} }}^{2} } \right)}}.$$

By ignoring the term $\left( { - \frac{1}{2}h_{{c_{A} }}^{2} r_{{c_{a}^{\left( A \right)} }}^{2} - \frac{1}{2}h_{{c_{B} }}^{2} r_{{c_{a}^{\left( B \right)} }}^{2} } \right)$ for low $h_{{c_{A} }}^{2}$ and $h_{{c_{B} }}^{2}$, the prediction equation simplifies to:

8 $$r_{C\_BSAM\_without}^{2} = \frac{{N_{AB} h_{{c_{A} }}^{2} }}{{N_{AB} h_{{c_{A} }}^{2} + 2Me_{a,AB}^{\left( A \right)} }}.$$

#### CB + PB–PB scenario

For the CB + PB–PB scenario, the reference population includes breed A and crossbred AB reference animals, each with their own phenotypes. The BSAM for the crossbred performance trait, assuming that each individual has one record corrected for all effects other than additive genetic effects, can be written as follows [3, 4]:

$$\left[ {\begin{array}{*{20}c} {{\mathbf{y}}_{A} } \\ {{\mathbf{y}}_{AB} } \\ \end{array} } \right] = \left[ {\begin{array}{*{20}c} {{\mathbf{Z}}_{A}^{\left( A \right)} } & {\quad {\mathbf{0}}} \\ {\mathbf{0}} & {\quad {\mathbf{Z}}_{AB}^{\left( A \right)} } \\ \end{array} } \right]\left[ {\begin{array}{*{20}c} {{\varvec{\upbeta}}_{a}^{\left( A \right)} } \\ {{\varvec{\upbeta}}_{c}^{\left( A \right)} } \\ \end{array} } \right] + \left[ {\begin{array}{*{20}c} {\mathbf{0}} & {\quad {\mathbf{0}}} \\ {\mathbf{0}} & {\quad {\mathbf{Z}}_{AB}^{\left( B \right)} } \\ \end{array} } \right]\left[ {\begin{array}{*{20}c} {\mathbf{0}} \\ {{\varvec{\upbeta}}_{c}^{\left( B \right)} } \\ \end{array} } \right] + \left[ {\begin{array}{*{20}c} {{\mathbf{e}}_{A} } \\ {{\mathbf{e}}_{AB} } \\ \end{array} } \right],$$

where ${\mathbf{y}}_{A}$ is the vector of corrected records of purebred performance, ${\mathbf{Z}}_{A}^{\left( A \right)}$ contains the standardized SNP genotypes of breed A reference animals, and ${\varvec{\upbeta}}_{a}^{\left( A \right)}$ is the vector of breed A allele substitution effects for all SNPs for purebred performance.

Equivalently, the previous BSAM can be written as [3]:

$$\left[ {\begin{array}{*{20}c} {{\mathbf{y}}_{A} } \\ {{\mathbf{y}}_{AB} } \\ \end{array} } \right] = \left[ {\begin{array}{*{20}c} {{\mathbf{a}}_{A}^{\left( A \right)} } \\ {{\mathbf{c}}_{AB}^{\left( A \right)} } \\ \end{array} } \right] + \left[ {\begin{array}{*{20}c} {\mathbf{0}} \\ {{\mathbf{c}}_{AB}^{\left( B \right)} } \\ \end{array} } \right] + \left[ {\begin{array}{*{20}c} {{\mathbf{e}}_{A} } \\ {{\mathbf{e}}_{AB} } \\ \end{array} } \right],$$

where ${\mathbf{a}}_{A}^{\left( A \right)} = {\mathbf{Z}}_{A}^{\left( A \right)} {\varvec{\upbeta}}_{a}^{\left( A \right)}$ is the vector of additive genetic effects for the purebred performance trait.

Expectations and variances and covariances of ${\mathbf{a}}_{A}^{\left( A \right)}$, ${\mathbf{c}}_{AB}^{\left( A \right)}$ and ${\mathbf{c}}_{AB}^{\left( B \right)}$ are assumed to be $$E\left[ {\begin{array}{*{20}c} {{\mathbf{a}}_{A}^{\left( A \right)} } \\ {{\mathbf{c}}_{AB}^{\left( A \right)} } \\ {{\mathbf{c}}_{AB}^{\left( B \right)} } \\ \end{array} } \right] = \left[ {\begin{array}{*{20}c} {\mathbf{0}} \\ {\mathbf{0}} \\ {\mathbf{0}} \\ \end{array} } \right]$$ and

$$Var\left[ {\begin{array}{*{20}c} {{\mathbf{a}}_{A}^{\left( A \right)} } \\ {{\mathbf{c}}_{AB}^{\left( A \right)} } \\ {{\mathbf{c}}_{AB}^{\left( B \right)} } \\ \end{array} } \right] = \left[ {\begin{array}{*{20}c} {{\mathbf{Z}}_{A}^{\left( A \right)} {\mathbf{Z}}_{A}^{\left( A \right)^{\prime} } \frac{{\sigma_{{a_{A} }}^{2} }}{m}} & {\quad {\mathbf{Z}}_{A}^{\left( A \right)} {\mathbf{Z}}_{AB}^{\left( A \right)^{\prime} } \frac{{\sigma_{{a_{A} c_{A} }} }}{m}} & {\quad {\mathbf{0}}} \\ {{\mathbf{Z}}_{AB}^{\left( A \right)} {\mathbf{Z}}_{A}^{\left( A \right)^{\prime} } \frac{{\sigma_{{a_{A} c_{A} }} }}{m}} & {\quad {\mathbf{Z}}_{AB}^{\left( A \right)} {\mathbf{Z}}_{AB}^{\left( A \right)^{\prime} } \frac{{\sigma_{{c_{A} }}^{2} }}{m}} & {\quad {\mathbf{0}}} \\ {\mathbf{0}} & {\quad {\mathbf{0}}} & {{\mathbf{Z}}_{AB}^{\left( B \right)} {\mathbf{Z}}_{AB}^{\left( B \right)^{\prime} } \frac{{\sigma_{{c_{B} }}^{2} }}{m}} \\ \end{array} } \right] = \left[ {\begin{array}{*{20}c} {{\mathbf{G}}_{A,A}^{\left( A \right)} \sigma_{{a_{A} }}^{2} } & {\quad {\mathbf{G}}_{A,AB}^{\left( A \right)} \sigma_{{a_{A} c_{A} }} } & {\quad {\mathbf{0}}} \\ {{\mathbf{G}}_{AB,A}^{\left( A \right)} \sigma_{{a_{A} c_{A} }} } & {\quad {\mathbf{G}}_{AB,AB}^{\left( A \right)} \sigma_{{c_{A} }}^{2} } & {\quad {\mathbf{0}}} \\ {\mathbf{0}} & {\quad {\mathbf{0}}} & {\quad {\mathbf{G}}_{AB,AB}^{\left( B \right)} \sigma_{{c_{B} }}^{2} } \\ \end{array} } \right],$$

where ${\mathbf{G}}_{A,A}^{\left( A \right)}$ is the breed A genomic relationship matrix between $N_{A}$ reference animals of breed A and ${\mathbf{G}}_{A,AB}^{\left( A \right)}$ is the breed A-specific partial genomic relationship matrix between $N_{A}$ selection candidates of breed A and $N_{AB}$ crossbred AB reference animals [3].

Based on the SI theory, genomic EBV for the crossbred performance trait for breed A selection candidates (${\mathbf{c}}_{a}^{\left( A \right)}$) can be predicted from records of breed A reference animals and of crossbred AB reference animals:

$${\hat{\mathbf{c}}}_{a}^{\left( A \right)} = Cov\left( {{\mathbf{c}}_{a}^{\left( A \right)} ,{\mathbf{y}}} \right)\left( {Var\left( {\mathbf{y}} \right)} \right)^{ - 1} {\mathbf{y}},$$

with $${\mathbf{y}} = \left[ {\begin{array}{*{20}c} {{\mathbf{y}}_{A} } \\ {{\mathbf{y}}_{AB} } \\ \end{array} } \right]$$.

Based on the model description, the variance of ${\mathbf{y}}$ is equal to:

$$Var\left( {\mathbf{y}} \right) = Var\left( {\left[ {\begin{array}{*{20}c} {{\mathbf{y}}_{A} } \\ {{\mathbf{y}}_{AB} } \\ \end{array} } \right]} \right) = \left[ {\begin{array}{*{20}c} {{\mathbf{G}}_{AA}^{\left( A \right)} \sigma_{{c_{A} }}^{2} + {\mathbf{I}}\sigma_{{e_{A} }}^{2} } & {\quad {\mathbf{G}}_{A,AB}^{\left( A \right)} \sigma_{{a_{A} c_{A} }} } \\ {{\mathbf{G}}_{A,AB}^{\left( A \right)} \sigma_{{a_{A} c_{A} }} } & {\quad {\mathbf{G}}_{ABAB}^{\left( A \right)} \sigma_{{c_{A} }}^{2} + {\mathbf{G}}_{ABAB}^{\left( B \right)} \sigma_{{c_{B} }}^{2} + {\mathbf{I}}\sigma_{{e_{AB} }}^{2} } \\ \end{array} } \right],$$

since:

$$Cov\left( {{\mathbf{y}}_{A} ,{\mathbf{y}}_{AB} } \right) = Cov\left( {{\mathbf{a}}_{A}^{\left( A \right)} + {\mathbf{e}}_{A} ,{\mathbf{c}}_{AB}^{\left( A \right)} + {\mathbf{c}}_{AB}^{\left( B \right)} + {\mathbf{e}}_{AB} } \right) = Cov\left( {{\mathbf{a}}_{A}^{\left( A \right)} ,{\mathbf{c}}_{AB}^{\left( A \right)} + {\mathbf{c}}_{AB}^{\left( B \right)} } \right) = Cov\left( {{\mathbf{a}}_{A}^{\left( A \right)} ,{\mathbf{c}}_{AB}^{\left( A \right)} } \right) = {\mathbf{G}}_{A,AB}^{\left( A \right)} \sigma_{{a_{A} c_{A} }} .$$

Similarly, the covariance between ${\mathbf{c}}_{a}^{\left( A \right)}$ and ${\mathbf{y}}$ is equal to:

$$Cov\left( {{\mathbf{c}}_{a}^{\left( A \right)} ,{\mathbf{y}}} \right) = \left[ {\begin{array}{*{20}c} {{\mathbf{G}}_{a,A}^{\left( A \right)} \sigma_{{a_{A} c_{A} }} } & {{\mathbf{G}}_{a,AB}^{\left( A \right)} \sigma_{{c_{A} }}^{2} } \\ \end{array} } \right].$$

The reliability of ${\hat{\text{c}}}_{{a_{i} }}^{\left( A \right)}$ of the *i*th selection candidate of breed A is then equal to:

$$ \begin{aligned} r_{{C + P\_{{BSAM\_{{with_{i} }} }} }}^{2} & = \frac{{\left( {Cov\left( {{\hat{\text{c}}}_{{a_{i} }}^{\left( A \right)} ,{\text{c}}_{{a_{i} }}^{\left( A \right)} } \right)} \right)^{2} }}{{Var\left( {{\hat{\text{c}}}_{{a_{i} }}^{\left( A \right)} } \right)Var\left( {{\text{c}}_{{a_{i} }}^{\left( A \right)} } \right)}} = \frac{{Var\left( {{\hat{\text{c}}}_{{a_{i} }}^{\left( A \right)} } \right)}}{{Var\left( {{\text{c}}_{{a_{i} }}^{\left( A \right)} } \right)}} = \frac{1}{{Var\left( {{\text{c}}_{{a_{i} }}^{\left( A \right)} } \right)}}\left[ {\begin{array}{*{20}c} {{\mathbf{G}}_{{a_{i} ,A}}^{\left( A \right)} \sigma_{{a_{A} c_{A} }} } & {{\mathbf{G}}_{{a_{i} ,AB}}^{\left( A \right)} \sigma_{{c_{A} }}^{2} } \\ \end{array} } \right] \times \left[ {\begin{array}{*{20}c} {{\mathbf{G}}_{A,A}^{\left( A \right)} \sigma_{{a_{A} }}^{2} + {\mathbf{I}}\sigma_{{e_{A} }}^{2} } & {\quad {\mathbf{G}}_{A,AB}^{\left( A \right)} \sigma_{{a_{A} c_{A} }} } \\ {{\mathbf{G}}_{A,AB}^{\left( A \right)} \sigma_{{a_{A} c_{A} }} } & {\quad {\mathbf{G}}_{AB,AB}^{\left( A \right)} \sigma_{{c_{A} }}^{2} + {\mathbf{G}}_{AB,AB}^{\left( B \right)} \sigma_{{c_{B} }}^{2} + {\mathbf{I}}\sigma_{{e_{AB} }}^{2} } \\ \end{array} } \right]^{ - 1} \left[ {\begin{array}{*{20}c} {{\mathbf{G}}_{{A,a_{i} }}^{\left( A \right)} \sigma_{{a_{A} c_{A} }} } \\ {{\mathbf{G}}_{{AB,a_{i} }}^{\left( A \right)} \sigma_{{c_{A} }}^{2} } \\ \end{array} } \right] \\ & = \frac{1}{{{\text{G}}_{{a_{i} ,a_{i} }}^{\left( A \right)} }}\left[ {\begin{array}{*{20}c} {r_{PC}^{\left( A \right)} {\mathbf{G}}_{{a_{i} ,A}}^{\left( A \right)} } & {{\mathbf{G}}_{{a_{i} A,B}}^{\left( A \right)} } \\ \end{array} } \right] \times \left[ {\begin{array}{*{20}c} {{\mathbf{G}}_{A,A}^{\left( A \right)} + {\mathbf{I}}\frac{{1 - h_{{a_{A} }}^{2} }}{{h_{{a_{A} }}^{2} }}} & {\quad r_{PC}^{\left( A \right)} {\mathbf{G}}_{A,AB}^{\left( A \right)} } \\ {r_{PC}^{\left( A \right)} {\mathbf{G}}_{AB,A}^{\left( A \right)} } & {\quad {\mathbf{G}}_{AB,AB}^{\left( A \right)} + {\mathbf{G}}_{AB,AB}^{\left( B \right)} \frac{{h_{{c_{B} }}^{2} }}{{h_{{c_{A} }}^{2} }} + {\mathbf{I}}\frac{{1 - \frac{1}{2}h_{{c_{A} }}^{2} - \frac{1}{2}h_{{c_{B} }}^{2} }}{{h_{{c_{A} }}^{2} }}} \\ \end{array} } \right]^{ - 1} \left[ {\begin{array}{*{20}c} {r_{PC}^{\left( A \right)} {\mathbf{G}}_{{A,a_{i} }}^{\left( A \right)} } \\ {{\mathbf{G}}_{{AB,a_{i} }}^{\left( A \right)} } \\ \end{array} } \right], \\ \end{aligned} $$

where the breed A-specific genetic correlation between purebred and crossbred performance traits $r_{PC}^{\left( A \right)}$ is equal to $r_{PC}^{\left( A \right)} = \frac{{\sigma_{{a_{A} c_{A} }} }}{{\sqrt {\sigma_{{a_{A} }}^{2} \sigma_{{c_{A} }}^{2} } }}$.

With availability of genotyping data, the average predicted reliability of genomic EBV across all breed A selection candidates is therefore equal to:

9 $$r_{C + P\_BSAM\_with}^{2} = \frac{1}{{N_{a} }}\mathop \sum \limits_{\text{i}} r_{{C + P\_BSAM\_with_{i} }}^{2} = \frac{1}{{{\text{G}}_{{a_{i} ,a_{i} }}^{\left( A \right)} }}\left[ {\begin{array}{*{20}c} {r_{PC}^{\left( A \right)} {\mathbf{G}}_{{a_{i} ,A}}^{\left( A \right)} } & {{\mathbf{G}}_{{a_{i} A,B}}^{\left( A \right)} } \\ \end{array} } \right] \times \frac{1}{{N_{a} }}\mathop \sum \limits_{i} \begin{array}{*{20}c} {} \\ {\left[ {\begin{array}{*{20}c} {{\mathbf{G}}_{A,A}^{\left( A \right)} + {\mathbf{I}}\frac{{1 - h_{{a_{A} }}^{2} }}{{h_{{a_{A} }}^{2} }}} & {r_{PC}^{\left( A \right)} {\mathbf{G}}_{A,AB}^{\left( A \right)} } \\ {r_{PC}^{\left( A \right)} {\mathbf{G}}_{AB,A}^{\left( A \right)} } & {{\mathbf{G}}_{AB,AB}^{\left( A \right)} + {\mathbf{G}}_{AB,AB}^{\left( B \right)} \frac{{h_{{c_{B} }}^{2} }}{{h_{{c_{A} }}^{2} }} + {\mathbf{I}}\frac{{1 - \frac{1}{2}h_{{c_{A} }}^{2} - \frac{1}{2}h_{{c_{B} }}^{2} }}{{h_{{c_{A} }}^{2} }}} \\ \end{array} } \right]^{ - 1} } \\ \end{array} \times \left[ {\begin{array}{*{20}c} {r_{PC}^{\left( A \right)} {\mathbf{G}}_{{A,a_{i} }}^{\left( A \right)} } \\ {{\mathbf{G}}_{{AB,a_{i} }}^{\left( A \right)} } \\ \end{array} } \right]$$

Without availability of genotyping data, the prediction equation for the reliability of genomic EBV based on BSAM, $r_{C + P\_BSAM\_without}^{2}$, can be derived similarly to the prediction equation for the CB–PB scenario, $r_{C\_BSAM\_with}^{2}$. The derivation is based on mixed model theory and assumes that independent allele substitution effects for breeds A and B for both purebred and crossbred performances were estimated simultaneously. The detailed derivation can be found in Additional file 3.

Consider $N_{A}$ unrelated genotyped breed A reference animals and $N_{AB}$ unrelated genotyped crossbred AB reference animals. Similar to the CB–PB scenario, the genomic EBV (${\text{c}}_{{a_{i} }}^{\left( A \right)}$) for the *i*th selection candidate of breed A can be predicted as ${\hat{\text{c}}}_{{a_{i} }}^{\left( A \right)} = {\mathbf{z}}_{{a_{i} }}^{{{*}\left( A \right)}} {\hat{\varvec{\upbeta }}}_{c}^{*\left( A \right)}$ and its reliability is equal to:

$$r_{{C + P\_BSAM\_without_{i} }}^{2} = \frac{{Var\left( {{\hat{\text{c}}}_{{a_{i} }}^{\left( A \right)} } \right)}}{{Var\left( {{\text{c}}_{{a_{i} }}^{\left( A \right)} } \right)}} = \frac{{Var\left( { {\hat{{\upbeta }}}_{{c_{k} }}^{*\left( A \right)} } \right)}}{{Var\left( { {{\upbeta }}_{{c_{k} }}^{ *\left( A \right)} } \right)}} = r_{{\beta_{c}^{*\left( A \right)} }}^{2} .$$

Reliability $r_{{\beta_{c}^{*\left( A \right)} }}^{2}$ can be approximated as follows. The prediction of ${{\upbeta }}_{{c_{k} }}^{*\left( A \right)}$ for the *k*th independent locus can be performed using the phenotypes of both purebred and crossbred performances, $$\left[ {\begin{array}{*{20}c} {\widehat{{{\mathbf{y}}_{A}^{*} }}} \\ {\widehat{{{\mathbf{y}}_{AB}^{*} }}} \\ \end{array} } \right]$$, corrected for all other fixed effects, as well as for the breed B-specific allele substitution effects and correlated effects, using the model:

$$\left[ {\begin{array}{*{20}c} {\widehat{{{\mathbf{y}}_{A}^{*} }}} \\ {\widehat{{{\mathbf{y}}_{AB}^{*} }}} \\ \end{array} } \right] = \left[ {\begin{array}{*{20}c} {{\mathbf{z}}_{{A_{k} }}^{{{*}\left( A \right)}} } & {\quad {\mathbf{0}}} \\ {\mathbf{0}} & {\quad {\mathbf{z}}_{{AB_{k} }}^{{{*}\left( A \right)}} } \\ \end{array} } \right]\left[ {\begin{array}{*{20}c} {{\hat{{\upbeta }}}_{{a_{k} }}^{*\left( A \right)} } \\ {{\hat{{\upbeta }}}_{{c_{k} }}^{*\left( A \right)} } \\ \end{array} } \right] + \left[ {\begin{array}{*{20}c} {{\varvec{\upvarepsilon}}_{{A_{k} }} } \\ {{\varvec{\upvarepsilon}}_{{AB_{k} }} } \\ \end{array} } \right],$$

where ${\varvec{\upvarepsilon}}_{{A_{k} }}$ is the residual vector. For simplicity, we will assume that $$Var\left( {\left[ {\begin{array}{*{20}c} {{\varvec{\upvarepsilon}}_{{A_{k} }} } \\ {{\varvec{\upvarepsilon}}_{{AB_{k} }} } \\ \end{array} } \right]} \right) = \left[ {\begin{array}{*{20}c} {{\mathbf{I}}\sigma_{{\varepsilon_{A} }}^{2} } & {\mathbf{0}} \\ {\mathbf{0}} & {{\mathbf{I}}\sigma_{{\varepsilon_{AB} }}^{2} } \\ \end{array} } \right]$$, with $\sigma_{{\varepsilon_{A} }}^{2}$ being the residual variance associated with $\widehat{{{\mathbf{y}}_{A}^{*} }}$. Then, following mixed model theory [17, 19], the prediction of $$\left[ {\begin{array}{*{20}c} {{{\upbeta }}_{{a_{k} }}^{ *\left( A \right)} } \\ {{{\upbeta }}_{{c_{k} }}^{ *\left( A \right)} } \\ \end{array} } \right]$$ is equal to:

$$\left[ {\begin{array}{*{20}c} {{\hat{{\upbeta }}}_{{a_{k} }}^{*\left( A \right)} } \\ {{\hat{{\upbeta }}}_{{c_{k} }}^{*\left( A \right)} } \\ \end{array} } \right] = \left( {\left[ {\begin{array}{*{20}c} {{\mathbf{z}}_{{A_{k} }}^{{{*}\left( A \right)^{\prime} }} } & {\quad {\mathbf{0}}} \\ {\quad {\mathbf{0}}} & {{\mathbf{z}}_{{AB_{k} }}^{{{*}\left( A \right)^{\prime} }} } \\ \end{array} } \right]\left[ {\begin{array}{*{20}c} {{\mathbf{I}}\sigma_{{\varepsilon_{A} }}^{ - 2} } & {\quad {\mathbf{0}}} \\ {\quad {\mathbf{0}}} & {{\mathbf{I}}\sigma_{{\varepsilon_{AB} }}^{ - 2} } \\ \end{array} } \right]\left[ {\begin{array}{*{20}c} {{\mathbf{z}}_{{A_{k} }}^{{{*}\left( A \right)}} } & {\quad {\mathbf{0}}} \\ {\quad {\mathbf{0}}} & {{\mathbf{z}}_{{AB_{k} }}^{{{*}\left( A \right)}} } \\ \end{array} } \right] + \left[ {\begin{array}{*{20}c} {\sigma_{{\beta_{a}^{*\left( A \right)} }}^{2} } & {\quad \sigma_{{\beta_{a}^{*\left( A \right)} \beta_{c}^{*\left( A \right)} }} } \\ {\sigma_{{\beta_{a}^{*\left( A \right)} \beta_{c}^{*\left( A \right)} }} } & {\quad \sigma_{{\beta_{c}^{*\left( A \right)} }}^{2} } \\ \end{array} } \right]^{ - 1} } \right)^{ - 1} \left[ {\begin{array}{*{20}c} {{\mathbf{z}}_{{A_{k} }}^{{{*}\left( A \right)^{\prime }}} } & {\quad {\mathbf{0}}} \\ {\quad {\mathbf{0}}} & {{\mathbf{z}}_{{AB_{k} }}^{{{*}\left( A \right)^{\prime} }} } \\ \end{array} } \right]\left[ {\begin{array}{*{20}c} {{\mathbf{I}}\sigma_{{\varepsilon_{A} }}^{ - 2} } & {\quad {\mathbf{0}}} \\ {\quad {\mathbf{0}}} & {{\mathbf{I}}\sigma_{{\varepsilon_{AB} }}^{ - 2} } \\ \end{array} } \right]\left[ {\begin{array}{*{20}c} {\widehat{{{\mathbf{y}}_{A}^{*} }}} \\ {\widehat{{{\mathbf{y}}_{AB}^{*} }}} \\ \end{array} } \right],$$

and the reliability of ${\hat{{\upbeta }}}_{{c_{k} }}^{*\left( A \right)}$ is equal to:

$$r_{{\beta_{c}^{*\left( A \right)} }}^{2} = \frac{{Var\left( {{{\upbeta }}_{{c_{k} }}^{ *\left( A \right)} } \right) - Var\left( {{\hat{{\upbeta }}}_{{c_{k} }}^{*\left( A \right)} - {{\upbeta }}_{{c_{k} }}^{ *\left( A \right)} } \right)}}{{Var\left( {{{\upbeta }}_{{c_{k} }}^{ *\left( A \right)} } \right)}} = \frac{{\sigma_{{\beta_{c}^{*\left( A \right)} }}^{2} - PEV_{{{\hat{{\beta }}}_{{c_{k} }}^{*\left( A \right)} }} }}{{\sigma_{{\beta_{c}^{*\left( A \right)} }}^{2} }},$$

where $PEV_{{{\hat{{\beta }}}_{{c_{k} }}^{*\left( A \right)} }}$ is the prediction error variance of ${\hat{{\upbeta }}}_{{c_{k} }}^{*\left( A \right)}$ and is equal to the diagonal element of the inverse of the left-hand side of the mixed model equations associated with the prediction of $$\left[ {\begin{array}{*{20}c} {{{\upbeta }}_{{a_{k} }}^{ *\left( A \right)} } \\ {{{\upbeta }}_{{c_{k} }}^{ *\left( A \right)} } \\ \end{array} } \right]$$. After some algebra, which is detailed in Additional file 3, it follows that the predicted reliability of genomic EBV for breed A selection candidates without data is equal to:

10 $$ r_{ C+ P{\_}BSAM{\_}without}^{2} = r_{{\beta_{c}^{*\left( A \right)} }}^{2} = \left[ {\begin{array}{*{20}c} {r_{PC}^{\left( A \right)} \sqrt {\frac{{h_{{a_{A} }}^{2} }}{{Me_{a,A} }}} } & {\sqrt {\frac{{h_{{c_{A} }}^{2} }}{{2Me_{a,AB}^{\left( A \right)} }}} } \\ \end{array} } \right] \times \left[ {\begin{array}{*{20}c} {\frac{{h_{{a_{A} }}^{2} }}{{Me_{a,A} }} + \frac{1}{{N_{A} }}} & {\quad r_{PC}^{\left( A \right)} \sqrt {\frac{{h_{{c_{A} }}^{2} }}{{2Me_{a,AB}^{\left( A \right)} }}\frac{{h_{{a_{A} }}^{2} }}{{Me_{a,A} }}} } \\ {r_{PC}^{\left( A \right)} \sqrt {\frac{{h_{{c_{A} }}^{2} }}{{2Me_{a,AB}^{\left( A \right)} }}\frac{{h_{{a_{A} }}^{2} }}{{Me_{a,A} }}} } & {\quad \frac{{h_{{c_{A} }}^{2} }}{{2Me_{a,AB}^{\left( A \right)} }} + \frac{1}{{N_{AB} }}} \\ \end{array} } \right]^{ - 1} \left[ {\begin{array}{*{20}c} {r_{PC}^{\left( A \right)} \sqrt {\frac{{h_{{a_{A} }}^{2} }}{{Me_{a,A} }}} } \\ {\sqrt {\frac{{h_{{c_{A} }}^{2} }}{{2Me_{a,AB}^{\left( A \right)} }}} } \\ \end{array} } \right] $$

### Computation of the effective number of chromosome segments (Me)

The prediction equations without availability of genotyping data require the effective number of chromosome segments that are independently segregating in a population, including selection candidates ($S$) and reference animals ($R$) (i.e., that are shared between the two populations), $Me_{S,R}$. The value of $Me_{S,R}$ can be computed as proposed by Wientjes et al. [14]:

$$Me_{S,R} = \frac{1}{{S_{{{\mathbf{G}}_{{{\text{S}},{\text{R}}}} - {\mathbf{A}}_{{{\text{S}},{\text{R}}}} }}^{2} }},$$

where ${\mathbf{G}}_{{{\text{S}},{\text{R}}}}$ is the genomic relationship matrix between selection candidates and reference animals, ${\mathbf{A}}_{{{\text{S}},{\text{R}}}}$ is the pedigree relationship matrix, and $S_{{{\mathbf{G}}_{{{\text{S}},{\text{R}}}} - {\mathbf{A}}_{{{\text{S}},{\text{R}}}} }}^{2}$ is the empirical variance of the differences between corresponding elements of ${\mathbf{G}}_{{{\text{S}},{\text{R}}}}$ and ${\mathbf{A}}_{{{\text{S}},{\text{R}}}}$. In our study, ${\mathbf{G}}_{{{\text{S}},{\text{R}}}}$ and ${\mathbf{A}}_{{{\text{S}},{\text{R}}}}$ were scaled to the same base population by rescaling the inbreeding level in ${\mathbf{G}}_{{{\text{S}},{\text{R}}}}$ to the inbreeding in ${\mathbf{A}}_{{{\text{S}},{\text{R}}}}$ as follows [20]:

$${\mathbf{G}}_{{{\text{S}},{\text{R}}}}^{ *} = \left( {1 - {\bar{\text{F}}}} \right){\mathbf{G}}_{{{\text{S}},{\text{R}}}} + 2{\bar{\text{F}}}{\mathbf{J}},$$

where ${\bar{\text{F}}}$ is the average pedigree inbreeding level computed from the pedigree, and ${\mathbf{J}}$ is a matrix filled with 1s.

The proposed computation of $Me$ requires genotypes for both selection candidates and reference animals, which may be inconsistent with its use in the computation of reliabilities without availability of genotyping data. However, it is reasonable to assume that genotypes are already available for a limited number of animals, for example at least 100, that have the right family structure that is representative of the evaluated scenario, such that an accurate approximation of $Me$ can be computed [14].

The effective number of chromosome segments originating from a specific breed (*b*) and that are shared between purebred selection candidates ($S$) of this breed and crossbred reference animals ($Rc$), $Me_{S,Rc}^{\left( b \right)}$, is required for the prediction equations for BSAM. In this study, $Me_{a,AB}^{\left( A \right)}$, is required in Eqs. (8) and (10) and was assumed to be equal to $Me_{a,A}$, which is required in Eqs. (2) and (6). The equality $Me_{a,AB}^{\left( A \right)} = Me_{a,A}$ was assumed since the selection candidates were the same for Eqs. (2), (4), (6), (8), and (10) the number of reference animals $R$ and $Rc$ was large, and the parents of breed A and crossbred AB reference animals were sampled from the same finite pool.

### Simulated data

Data were simulated to validate Eqs. (2), (4), (6), (8), and (10), which predict the reliability of genomic EBV for crossbred performance using ASGM or BSAM, without availability of genotyping data. Two extreme scenarios were considered, in which either two closely-related or two unrelated breeds were used to produce crossbred animals. The reliabilities predicted by Eqs. (2), (4), (6), (8), and (10) were validated against the reliabilities computed with the corresponding prediction equations with availability of genotyping data, that is, Eqs. (1), (3), (5), (7), and (9). The reliabilities predicted by equations with availability of genotyping data are equivalent to those computed from PEV associated with selection candidates of a genomic best linear unbiased prediction including both reference animals and selection candidates, based on phenotypes corrected with the best linear unbiased estimates of the fixed effects, and assuming the absence of selection [15].

#### Populations

**Fig. 1:**
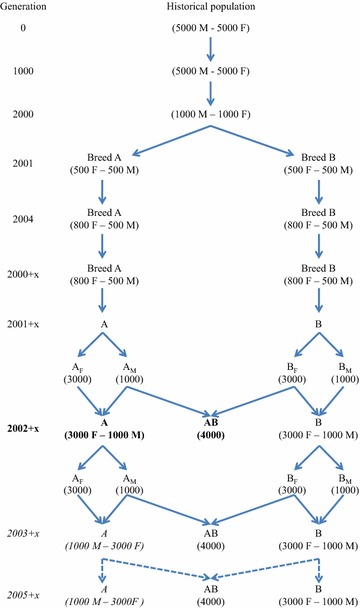
Schematic representation of the simulation. The crossbreeding program started at generation 2001 + x, with x being equal to 10 for the scenario with related breeds and equal to 100 for the scenario with unrelated breeds. The number of males (M) and females (F) per generation are reported within *brackets*. Reference animals were randomly selected from generation 2002 + x (in *bold*). Breed A selection candidates were randomly selected from generations 2003 + x, 2004 + x, and 2005 + x (in *italic characters*)

First, historical and breed populations were simulated using the QMSim software [21]; second, a two-way crossbreeding program with five generations of random selection was simulated using a customized Fortran program. For the historical population, 1000 discrete random mating generations with a constant size of 10,000 individuals were simulated, which was followed by 1000 generations in which the population size was gradually decreased to 2000 individuals. In these 2000 historical generations, half of the simulated animals were males and the other half were females. Offspring were produced by the random union of gametes from the male and female gametic pools, and the number of offspring was equal to the number of animals required in the next generation. To simulate the two breed populations, A and B, two random samples were drawn from the last generation of the historical population (i.e., generation 2000), each including 500 males and 500 females. Subsequently, within each of the breeds, 10 or 100 generations of random mating were simulated before the two-way crossbreeding scheme was begun. These two scenarios (i.e., a common origin either 10 or 100 generations ago) will be referred to as related and unrelated breeds, respectively. For the 10 and 100 generations of random mating, a litter of four offspring (two males and two females) per female was simulated. From these offspring, 500 males were selected at random for the next generation. The number of females selected randomly for the next generation was gradually increased from 500 to 800 during the first four generations of the simulation of the breed populations, in order to enlarge the size of the population (Fig. 1).

In a second step, a two-way crossbreeding program with five generations of random selection was simulated. The animals of breeds A and B that were used to start the crossbreeding program were sampled from generation 2010 for the related breeds and from generation 2100 for the unrelated breeds. During the crossbreeding program, and for both breeds, animals of breeds A and B were randomly selected and mated to simulate the next generation of a constant size of 1000 males and 3000 females for each breed. From each of these five generations, animals of breeds A and B were randomly crossed to produce five generations of 4000 crossbred AB animals. Purebred animals used as parents of crossbred animals could also be parents of the next generation of purebred animals (Fig. 1).

#### Genotypes

The total length of the simulated genome was 10 Morgans (M) (10 chromosomes of 1 M and 4000 SNPs each). The positions of SNPs and of recombinations were randomized per chromosome and a recurrent mutation rate of 2.5 × 10^−4^ was assumed. All SNPs with a minor allele frequency (MAF) higher than or equal to 0.05 in the last historical generation (i.e., generation 2000) and were used to simulate the SNP genotypes of the purebred and crossbred animals. For subsequent analyses, 2000 SNPs were randomly selected from these SNPs for each chromosome. The breed origin of each allele for each crossbred animal was recorded. All scenarios (including the historical populations) were replicated 10 times.

#### Validation of prediction equations without availability of genotyping data

The validation required a set of known genotypes, as described previously, but no phenotype, since the reliabilities predicted without availability of genotyping data were validated against the reliabilities predicted with availability of genotyping data. However, estimates of heritabilities and genetic correlations between purebred and crossbred performance were required. Heritabilities of 0.20, 0.40, and 0.95 were used for both the purebred and crossbred performance traits. A high heritability, such as 0.95, and a single record per reference animal can be assumed when phenotypes of reference animals are derived from highly reliable EBV (e.g., deregressed EBV) [10]. Genetic correlations between purebred and crossbred performance traits were assumed to be equal to 0.30 or 0.70.

In the simulated data, two groups of reference animals and one group of selection candidates were defined for each scenario of related and unrelated breeds. For the scenarios with related and unrelated breeds, the two groups of reference animals were randomly selected from generations 2012 and 2102, respectively. For scenarios PB–PB and CB–PB, the two groups of reference animals included 2000 and 4000 animals that were randomly chosen from breed A and crossbred AB animals, respectively. For scenario CB + PB–PB, the first group included 4000 randomly chosen breed A animals and 2000 randomly chosen crossbred AB animals and the second group included 4000 breed A animals and 4000 crossbred AB animals. For the selection candidates for scenarios PB–PB, CB–PB and CB + PB–PB, 1000 breed A animals were randomly selected from each generation, starting from generation 2013 for the related breeds scenario and from generation 2103 for the unrelated breeds scenario, to create the groups of selection candidates. In the following, selection candidates from generations 2013 or 2103 are referred to as “G1” selection candidates. Similarly, selection candidates from generations 2014 and 2104 and from generations 2015 and 2105 are referred to as “G2” and “G3” selection candidates, respectively.

For each ‘reference population-selection candidates’ combination and for each scenario, reliabilities of the genomic EBV for crossbred performance were computed using Eqs. (1), (3), (5), (7), and (9) for the scenarios in which all data was available, and using Eqs. (2), (4), (6), (8), and (10) for scenarios without availability of genotyping data. The required genomic relationship matrices and values of $Me$ were computed using our in-house software calc_grm [22]. The predicted reliabilities were averaged across the 10 replicates.

### Application of a prediction equation

The proposed equations can be used to investigate the reliability of genomic EBV for crossbred performance in crossbreeding schemes. As an illustration, Eq. (10), which predicts the reliability of genomic EBV using both purebred and crossbred animals as reference animals by BSAM, was used to predict the reliability of genomic EBV for a pig production system for which 10,000 breed A animals were previously genotyped and phenotyped. The aim was to investigate the effect of the addition of crossbred AB animals to the reference population on the reliability of genomic EBV for crossbred performance. A heritability of 0.20 was assumed for both purebred and crossbred performance traits and the genetic correlation between purebred and crossbred performance traits for breed A, ($r_{PC}^{\left( A \right)}$), ranged from 0.0 to 1.0. Both values of $Me$ required by Eq. (10) (i.e. $Me_{a,AB}^{\left( A \right)}$ and $Me_{a,A}$) were assumed to be equal to 476.6, based on the equation $Me = 2N_{e} L/\left( {\ln \left( {4N_{e} L} \right)} \right)$ [23], with $N_{e}$ being the effective population size and $L$ being the total length of the genome in M. For $N_{e}$ and $L$, we assumed values of 80 and 27 respectively, based on the study of Landrace pigs by Uimari and Tapio [24] and the study by Lin et al. [25]. The use of equal values of $Me$ for the purebred and crossbred populations was based on the assumption that breed A parents of purebred and crossbred animals were sampled from the same pool.

## Results

This section first presents the results of the validation of the equations for predicting reliability without availability of genotyping data. As defined previously, the reliabilities without availability of genotyping data were validated against the reliabilities computed with availability of genotyping data. The second part of this section describes the increase in reliabilities from the addition of crossbred animals to a purebred reference population in a pig breeding program.

### PB–PB scenario

**Fig. 2:**
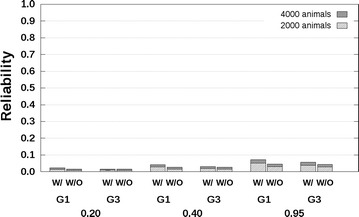
Reliabilities with a purebred reference population and a genetic correlation equal to 0.3. Reliabilities of genomic estimated breeding values for crossbred performance with (W/) and without (W/O) availability of genotyping data, using a reference population with 2000 or 4000 breed A animals, which are separated from breed A selection candidates by one (G1), or three (G3) generation(s). Heritabilities of 0.20, 0.40, and 0.95, were assumed. Results were averaged across replicates

**Fig. 3:**
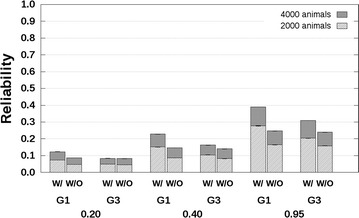
Reliabilities with a purebred reference population and a genetic correlation equal to 0.7. Reliabilities of genomic estimated breeding values for crossbred performance with (W/) and without (W/O) availability of genotyping data, using a reference population with 2000 or 4000 breed A animals, which are separated from breed A selection candidates by one (G1), or three (G3) generation(s). Heritabilities of 0.20, 0.40, and 0.95, were assumed. Results were averaged across replicates

For the PB–PB scenario, the results show that reliabilities predicted without availability of genotyping data were of the same order of magnitude as reliabilities computed with availability of genotyping data (Figs. 2, 3). For the scenario with related breeds and $r_{PC} = 0.3$ (Fig. 2), the predicted reliabilities with availability of genotyping data were around 0.01 for $h_{a}^{2} = 0.2$, in the range [0.02; 0.03] for $h_{a}^{2} = 0.4$, and in the range [0.04; 0.05] for $h_{a}^{2} = 0.95$, across all three groups of G1, G2 or G3 selection candidates and with 2000 reference animals from breed A. When $r_{PC} = 0.7$ (Fig. 3), the corresponding predicted reliabilities with availability of genotyping data were in the range [0.05; 0.07] for $h_{a}^{2} = 0.2$, in the range [0.10; 0.15] for $h_{a}^{2} = 0.4$, and in the range [0.20; 0.28] for $h_{a}^{2} = 0.95$. For both scenarios with $r_{PC} = 0.3$ and $r_{PC} = 0.7$, the addition of 2000 breed A reference animals slightly increased the predicted reliabilities (Figs. 2, 3).

Reliabilities predicted without availability of genotyping data were always lower than those predicted with availability of genotyping data, which agrees with theory (see “PB–PB scenario” section in the “Methods” section). For the scenario with related breeds and $r_{PC} = 0.3$ (Fig. 2), the differences between reliabilities predicted without and with availability of genotyping data were around 0.00 for $h_{a}^{2} = 0.2$, in the range [−0.02; 0.00] for $h_{a}^{2} = 0.4$, and in the range [−0.02; −0.01] for $h_{a}^{2} = 0.95$ across all three groups of G1, G2 or G3 selection candidates and with 2000 breed A reference animals. When $r_{PC} = 0.7$ (Fig. 3), the corresponding differences between reliabilities predicted without and with availability of genotyping data were in the range [−0.03; 0.00] for $h_{a}^{2} = 0.2$, in the range [−0.06; −0.02] for $h_{a}^{2} = 0.4$, and in the range [−0.11; −0.04] for $h_{a}^{2} = 0.95$. The largest differences between reliabilities predicted without and with availability of genotyping data were always observed for the G1 selection candidates.

Similar results were obtained for the scenario with unrelated breeds (see Additional file 4: Tables S1, S2). Such similar results were expected since the distance between breeds is not taken into account by ASGM. The SD of the reliabilities across replicates were in the range [0.000; 0.001] (see Additional file 4: Tables S1, S2).

### CB–PB scenario

**Fig. 4:**
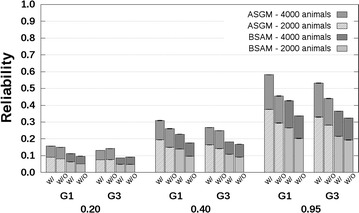
Reliabilities with a crossbred reference population that originated from two related breeds. Reliabilities of genomic estimated breeding values for crossbred performance with (W/) and without (W/O) availability of genotyping data, based on an across-breed SNP genotype model (ASGM) or on a breed-specific allele substitution effects model (BSAM), and using a reference population with 2000 or 4000 crossbred AB animals. Reference animals were separated from breed A selection candidates by one (G1), or three (G3) generation(s). Heritabilities of 0.20, 0.40, and 0.95, were assumed. Results were averaged across replicates

**Fig. 5:**
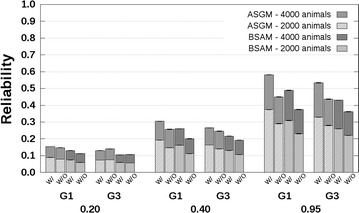
Reliabilities with a crossbred reference population that originated from two unrelated breeds. Reliabilities of genomic estimated breeding values for crossbred performance with (W/) and without (W/O) availability of genotyping data, based on an across-breed SNP genotype model (ASGM) or on a breed-specific allele substitution effect model (BSAM), and using a reference population with 2000 or 4000 crossbred AB animals. Reference animals were separated from breed A selection candidates by one (G1), or three (G3) generation(s). Heritabilities of 0.20, 0.40, and 0.95, were assumed. Results were averaged across replicates

Reliabilities with and without availability of genotyping data are presented in Fig. 4 for related breeds and in Fig. 5 for unrelated breeds. The CB–PB scenario included both ASGM and BSAM. For both models, the reliabilities predicted without availability of genotyping data underestimated the reliabilities predicted with availability of genotyping data. Underestimation was close to 0 when $h_{c}^{2} = 0.20$, and increased up to 0.1 with increasing $h_{c}^{2}$ and number of crossbred AB reference animals. Similar to the PB–PB scenario, the underestimation of reliabilities predicted with availability of genotyping data by the reliabilities predicted without availability of genotyping data was largest for the G1 selection candidates.

For the G1 selection candidates, the reliabilities for ASGM with availability of genotyping data were around 0.09 with 2000 crossbred reference animals, independent of the relationship between the breeds, and around 0.16 with 4000 crossbred reference animals, using $h_{c}^{2} = 0.20$ (Figs. 4, 5). Differences between the reliabilities predicted without and with availability of genotyping data were around −0.01 for both 2000 and 4000 crossbred reference animals. The corresponding reliabilities using $h_{c}^{2} = 0.95$ were around 0.37 and 0.58 with 2000 and 4000 crossbred reference animals, respectively. The corresponding differences between reliabilities predicted without and with availability of genotyping data were in the range [−0.13; −0.08].

For G1 selection candidates with related breeds, the reliabilities for BSAM with availability of genotyping data were around 0.06 and 0.11 with 2000 and 4000 crossbred reference animals, respectively, when using $h_{c}^{2} = 0.20$ (Fig. 4). Differences between reliabilities predicted without and with availability of genotyping data were around −0.01 with both 2000 and 4000 crossbred reference animals. The corresponding reliabilities using $h_{c}^{2} = 0.95$ were around 0.27 and 0.43 with 2000 and 4000 crossbred reference animals, respectively. Corresponding differences between reliabilities predicted without and with availability of genotyping data were in the range [−0.09; −0.06]. Similar differences were observed with unrelated breeds (Fig. 5). The SD of reliabilities across replicates were in the range [0.000; 0.002] (see Additional file 4: Tables S3, S4).

A comparison of reliabilities with availability of genotyping data between ASGM and BSAM showed that ASGM consistently performed better than BSAM. However, reliabilities for BSAM increased with increasing distance between breeds, while reliabilities for ASGM were only slightly affected (Figs. 4, 5). The increase in reliabilities with increasing distance between breeds, which compensates for the larger number of effects fitted in BSAM compared to ASGM, is in agreement with previous studies, e.g., Ibanez-Escriche et al. [4].

### CB + PB–PB scenario

The CB + PB–PB scenario included both breed A and crossbred AB animals in the reference population. The number of breed A reference animals was always 4000. The number of crossbred AB animals was equal to 2000 or 4000. The CB + PB–PB scenario also included both ASGM and BSAM.

**Fig. 6:**
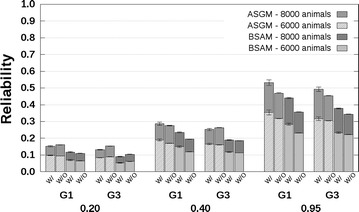
Reliabilities with a mixed reference population assuming two related breeds and a genetic correlation of 0.3. Reliabilities of genomic estimated breeding values for crossbred performance with (W/) and without (W/O) availability of genotyping data, based on an across-breed SNP genotype model (ASGM) or on a breed-specific allele substitution effects model (BSAM). The reference population included 4000 breed A animals and 2000 or 4000 crossbred AB animals. Reference animals were separated from breed A selection candidates by one (G1), or three (G3) generation(s). Heritabilities of 0.20, 0.40, and 0.95, were assumed. Results were averaged across replicates

**Fig. 7:**
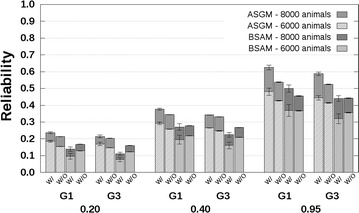
Reliabilities with a mixed reference population assuming two related breeds and a genetic correlation of 0.7. Reliabilities of genomic estimated breeding values for crossbred performance with (W/) and without (W/O) availability of genotyping data, based on an across-breed SNP genotype model (ASGM) or on a breed-specific allele substitution effects model (BSAM). The reference population included 4000 breed A animals and 2000 or 4000 crossbred AB animals. Reference animals were separated from breed A selection candidates by one (G1), or three (G3) generation(s). Heritabilities of 0.20, 0.40, and 0.95, were assumed. Results were averaged across replicates

For related breeds, reliabilities without and with availability of genotyping data are presented in Fig. 6 for $r_{PC} = 0.3$ and in Fig. 7 for $r_{PC} = 0.7$. Reliabilities predicted without and with availability of genotyping data were of the same order of magnitude, for both ASGM and BSAM. Differences between the two predicted reliabilities were in the range [−0.09; 0.05]. Similar to previous results, these differences increased with heritability. Reliabilities for BSAM with availability of genotyping data were about 0.03 to 0.04 lower than the corresponding reliabilities for ASGM when heritabilities were assumed to be 0.20. This difference between reliabilities for BSAM and for ASGM increased with increasing heritability and $r_{PC}$, and with decreasing distance between breeds. For example, reliabilities for BSAM with availability of genotyping data were between 0.07 and 0.12 points lower than the corresponding reliabilities for ASGM when heritabilities were equal to 0.95. These lower reliabilities for BSAM can be attributed to the additional breed-specific effects fitted in the model for a given number of records. Similar trends were observed for reliabilities without availability of genotyping data.

**Fig. 8:**
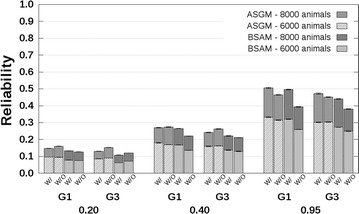
Reliabilities with a mixed reference population assuming two unrelated breeds and a genetic correlation of 0.3. Reliabilities of genomic estimated breeding values for crossbred performance with (W/) and without (W/O) availability of genotyping data, based on an across-breed SNP genotype model (ASGM) or on a breed-specific allele substitution effects model (BSAM). The reference population included 4000 breed A animals and 2000 or 4000 crossbred AB animals. Reference animals were separated from breed A selection candidates by one (G1), or three (G3) generation(s). Heritabilities of 0.20, 0.40, and 0.95, were assumed. Results were averaged across replicates

**Fig. 9:**
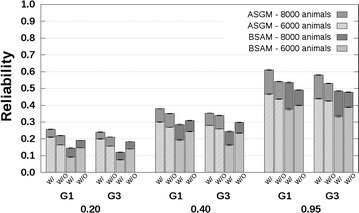
Reliabilities with a mixed reference population assuming two unrelated breeds and a genetic correlation of 0.7. Reliabilities of genomic estimated breeding values for crossbred performance with (W/) and without (W/O) availability of genotyping data, based on an across-breed SNP genotype model (ASGM) or on a breed-specific allele substitution effects model (BSAM). The reference population included 4000 breed A animals and 2000 or 4000 crossbred AB animals. Reference animals were separated from breed A selection candidates by one (G1), or three (G3) generation(s). Heritabilities of 0.20, 0.40, and 0.95, were assumed. Results were averaged across replicates

With unrelated breeds, the reliabilities without and with availability of genotyping data, averaged across replicates, are presented in Fig. 8 for $r_{PC} = 0.3$ and in Fig. 9 for $r_{PC} = 0.7$. Differences between reliabilities predicted without and with availability of genotyping data were in the range [−0.04; 0.07] for all scenarios with heritabilities equal to 0.20 and to 0.40, and in the range [−0.10; 0.05] for all scenarios with heritabilities equal to 0.95. Reliabilities predicted with availability of genotyping data for BSAM with unrelated breeds were higher by about 0.01 to 0.06 than the reliabilities predicted with availability of genotyping data for BSAM with related breeds. Similar trends were observed for reliabilities predicted without availability of genotyping data, showing a reliable prediction of reliability computed with availability of genotyping data. The SD of reliabilities across replicates were in the range [0.000; 0.006] (see Additional file 4: Tables S5, S6, S7, S8).

### Reliabilities in a pig-breeding program

**Fig. 10:**
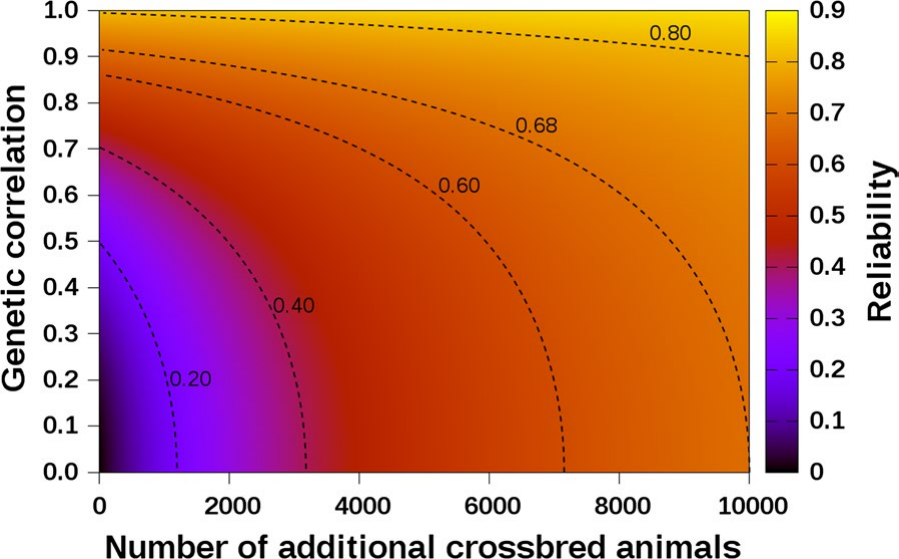
Reliabilities with additional crossbred reference animals and different genetic correlations. Reliabilities predicted without availability of genotyping data for genomic estimated breeding values of crossbred performance using a breed-specific allele substitution effects model. The reference population included 10,000 purebred animals and a number of crossbred animals that varied from 0 to 10,000. A heritability of 0.20 was assumed for both purebred and crossbred performance traits, and the genetic correlation between purebred and crossbred performance traits varied from 0.0 to 1.0. All the required values of $Me$ were assumed to be equal to 476.6

Predicted reliabilities for BSAM when up to 10,000 crossbred AB animals were added to a reference population of 10,000 breed A animals are in Fig. 10, showing that predicted reliabilities increased when crossbred AB animals were added to the reference population. The increase in reliabilities decreased with increasing $r_{PC}^{\left( A \right)}$. The reliabilities obtained for ASGM with $r_{PC}^{\left( A \right)} = 0.92$ based on 10,000 breed A reference animals (and no crossbred AB reference animals) (0.68) was the same as that for BSAM based on only 10,000 crossbred AB reference animals (i.e., with $r_{PC}^{\left( A \right)} = 0.0$). Therefore, for $r_{PC}^{\left( A \right)} < 0.92$, BSAM with only crossbred reference animals can be at least as accurate as ASGM with a larger number of purebred reference animals.

## Discussion

In this study, the term “reliability” refers to the precision of genomic EBV obtained by relating their PEV to the additive genetic variance of the base population, i.e., assuming absence of selection. Equations for predicting the reliability of genomic EBV for crossbred performance are proposed for reference populations that include purebred animals, crossbred animals, or both. Reliabilities were predicted for two models: ASGM and BSAM. For the BSAM, we used the true breed-of-origin of all alleles for the crossbred animals, which would have to be estimated in practice, which may negatively impact the reliability obtained. However, we expect this to have only a very minor effect, since we showed in previous studies that it is possible to accurately derive breed-of-origin of alleles in three-breed crossbred pigs [26, 27].

Reliabilities of genomic EBV can be predicted when genotype data are already available, i.e., with availability of genotyping data, or without availability of genotyping data. For scenarios without availability of genotyping data, it is assumed that the required genetic parameters are computed using pedigree instead of genomic data, or that estimates are available from the literature. The results of this study showed that the reliabilities of genomic EBV for crossbred performance predicted without availability of genotyping data were of the same order of magnitude as those predicted with availability of genotyping data. Therefore, while prediction of reliability should preferably take the genotype data of selection candidates into account when available, both methods can predict the reliability of genomic EBV for crossbred performance for different reference populations, heritabilities, and $r_{PC}$. The derived equations can therefore be useful to optimize the design of breeding programs.

### Reliabilities predicted without and with availability of genotyping data

The aim of this study was to predict the precision of genomic EBV based on PEV in the absence of selection. Thus, the derivation of our prediction equations without and with availability of genotyping data was based on the SI and mixed model theories and assumed that phenotypes were corrected for all fixed and random effects other than the considered genetic additive effects. The equivalence between SI and mixed model theories under certain conditions, such as the use of the same estimates for the fixed effects, has previously been shown by several studies (e.g., [15, 17, 28, 29]). Therefore, reliabilities predicted with availability of genotyping data would be expected to be close to reliabilities computed from PEV obtained from genomic best linear unbiased prediction, in the absence of selection. Equations for predicting the reliability of genomic EBV without availability of genotyping data were validated against the equations for predicting reliability with availability of genotyping data, and not against the reliability of selection, i.e., the squared correlation between estimated and true genomic breeding values, which is often obtained by cross-validation. Indeed, the reliability of genomic EBV is not equivalent to the reliability of selection for populations that are under selection, although they are equivalent for populations without selection [30–33]. Reliability of selection can be predicted from the reliability of genomic EBV by considering the intensity of selection using, e.g., the equations proposed by Dekkers [30] and Bijma [31].

Our prediction equations with availability of genotyping data can be extended to situations when phenotypes are already available for selection candidates and even to situations when some reference animals are not genotyped. First, in our study, selection candidates were defined as animals with genotypes but without phenotypes. In the context of poultry and pig breeding, this reflects for instance carcass, disease, or fertility traits. However, for growth-related traits, phenotypes are typically available for selection candidates at the time of selection. For these situations, prediction equations with availability of genotyping data can be used simply by including the selection candidates with genotypes and phenotypes as both reference animals and selection candidates, such that they are used both for computing the genomic relationship matrix between reference animals and selection candidates and the genomic relationship matrix between reference animals (e.g., for computing both ${\text{G}}_{{{\text{a}}_{\text{i}} ,{\text{A}}}}$ and ${\text{G}}_{{{\text{A}},{\text{A}}}}$ for the PB–PB scenario using ASGM). Second, situations in which some reference animals are not genotyped can also be modelled by our prediction equations with availability of genotyping data through the use of a combined pedigree-genomic relationship matrix ${\text{H}}$ [34–36] instead of the genomic relationship matrix ${\mathbf{G}}$ used in this study. Third, our prediction equations with availability of genotyping data can be extended to situations in which reference animals have repeated phenotypes, or pseudo-phenotypes, such as deregressed proofs and associated weights. For instance, the predicted reliability for the PB–PB scenario using ASGM can be computed as:

$$r_{P\_ASGM\_with}^{2} = \frac{1}{{N_{a} }}\mathop \sum \limits_{i} r_{PC}^{2} \frac{{{\mathbf{G}}_{{a_{i} ,A}} {\mathbf{W}}^{\prime } \left( {{\mathbf{WG}}_{A,A}^{{}} {\mathbf{W}}^{\prime } + {\mathbf{R}}\frac{{1 - h_{a}^{2} }}{{h_{a}^{2} }}} \right)^{ - 1} {\mathbf{WG}}_{{A,a_{i} }} }}{{{\mathbf{G}}_{{a_{i} ,a_{i} }} }},$$

where ${\mathbf{W}}$ is the incidence matrix relating (pseudo-)phenotypes to reference animals and ${\mathbf{R}}$ is a diagonal matrix with elements equal to 1 for real phenotypes, or equal to the inverse of weights associated with pseudo-phenotypes [17, 28]. Unlike the prediction equations with availability of genotyping data, the extension of prediction equations without availability of genotyping data to more complex scenarios is not as straightforward.

We also assumed that all additive genetic variance was captured by the SNPs in the derivation of the prediction equations. When only a portion of the additive genetic variance is captured by the SNPs, the prediction equations need to take this into account, as proposed by Goddard et al. [13] and Wientjes et al. [14]. This proportion could be empirically estimated when the reference population includes only one population by comparing predicted and realized (cross-validation) reliabilities [14].

For most scenarios, predicted reliabilities without availability of genotyping data underestimated the reliabilities predicted with availability of genotyping data (Figs. 2, 3, 4, 5, 6, 7, 8, 9). While this is in agreement with the theory, only a part of the underestimation is due to the fact that the decrease of the error variance when multiple loci are used was ignored (see “Methods” section; [12, 13]). This underestimation is greater when heritability and reliability increase to a value of 1 [13], as observed in our results. Most of the underestimation is, however, primarily due to an overestimation of $Me$, especially for the PB–PB and CB–PB scenarios with only one generation separating reference animals and selection candidates, for which the largest underestimations were observed. For instance, the fractional underestimation of $r_{P\_ASGM\_with}^{2}$ that can be attributed to not considering the reduction in error variance $\left( {1 - h_{a}^{2} r_{P\_ASGM\_without}^{2} } \right)$ (see Additional file 1) is approximately equal to $0.03$ (i.e., 3% error) for the PB–PB scenario with one generation separating 4000 reference animals and selection candidates, $h_{a}^{2} = 0.95$, $r_{PC} = 1.0$, and $Me_{a,A} = 3730$ (obtained from one random replicate). This does, however, explains only part of the fractional underestimation of about 0.57 that is observed in Fig. 3. Thus, the underestimation appears to be mainly due to the overestimation of $Me$, particularly when only one generation separates the reference animals and selection candidates. Indeed, while estimates of $Me$ increased with decreasing predicted reliabilities with availability of genotyping data, the results show that the reliabilities predicted without availability of genotyping data decreased at a lower rate than reliabilities predicted with availability of genotyping data when the relationships between the reference population and the selection candidates decreased. Further work to improve estimation of $Me$ is needed, especially for scenarios in which reference animals and selection candidates are highly related.

While predicted reliabilities without availability of genotyping data were underestimated for most scenarios, overestimations were observed for some scenarios with reference populations that included both purebred and crossbred animals (Figs. 6, 7, 8, 9). These overestimations may be the result of estimation of $Me$ and assumptions taken for the derivation of the equations without availability of genotyping data (e.g., a diagonal residual (co)variance matrix for corrected phenotypes associated with BSAM and using purebred and crossbred reference animals).

### Potential use of the prediction equations

The equations derived in this study can be used to compare the effects of modifying the values of various factors (e.g., $r_{PC}$, numbers of reference animals, or relationships between the reference population and the selection candidates) on the reliability of genomic EBV for crossbred performance and for the optimization of the design of breeding programs. However, the effects of some factors should be compared carefully. For example, the results show that the prediction equations without availability of genotyping data should be used with care for the comparison of the effects of different relationships between the reference population and the selection candidates. The prediction equations without availability of genotyping data should also be used with care for the comparison of the reliabilities of the ASGM and BSAM models, especially when the reference population includes both purebred and crossbred animals (e.g., for the PB + CB–PB scenario with unrelated breeds and $r_{PC} = 0.7$; Fig. 9). Nevertheless, the prediction equations without availability of genotyping data can still provide some insight into the reliability of both models in different scenarios. For instance, the results (Figs. 4, 5, 6, 7, 8, 9) showed that reliabilities for BSAM tended to increase with increasing distance between breeds, while the reliabilities for ASGM were only slightly affected. The increase in reliabilities with increasing distance between breeds, which compensates for fitting more effects in BSAM in comparison to ASGM, is in agreement with previous studies, e.g., Ibanez-Escriche et al. [4]. For instance, assume that a reference population of a fixed number of crossbred AB animals is available, and that heritabilities of crossbred performance traits estimated for ASGM and BSAM for the breed A are equal. Therefore, from Eq. (4), $r_{C\_ASGM\_without}^{2} = \frac{{N_{AB} h_{c}^{2} }}{{N_{AB} h_{c}^{2} + Me_{a,AB} }}$, and Eq. (8), $r_{C\_BSAM\_without}^{2} = \frac{{N_{AB} h_{{c_{A} }}^{2} }}{{N_{AB} h_{{c_{A} }}^{2} + 2Me_{a,AB}^{\left( A \right)} }}$, it follows that the reliability of genomic EBV based on BSAM would be higher than the reliability based on ASGM if $Me_{a,AB} > 2Me_{a,AB}^{\left( A \right)}$. This will be the case if the LD patterns between breeds A and B are sufficiently different, which is more likely in the case when the breeds have diverged for many generations [37]. This is in agreement with our results (Figs. 4, 5) and previous studies based on simulated data (e.g., [4, 11]) which show that reliabilities for BSAM increase with increasing distance between breeds. The additional effects fitted in BSAM are taken into account in Eq. (8) by the factor of 2, which was also considered by van Grevenhof and van der Werf [38], who evaluated the benefit of including crossbred animals in the reference population of a crossbreeding program using genomic selection.

### Computation of *Me*

The evaluation of different scenarios based on the prediction equations without availability of genotyping data requires accurate estimates of all parameters, and especially of $Me$ (e.g., [10, 14, 39, 40]). Parameters such as heritabilities and correlations, if estimated inaccurately, would similarly bias reliabilities predicted without and with availability of genotyping data, since these parameters are used in both equations. However, $Me$, the effective number of segments that are shared and segregating in both selection candidates and reference animals, is only used when predicting reliability without availability of genotyping data, and has a large impact. In our study, the estimates of $Me$ were computed from the differences between genomic and pedigree relationships between reference animals and selection candidates, as proposed by Wientjes et al. [14]. However, our results showed that these estimates of $Me$ did not adequately consider the close relationships that can exist between reference animals and selection candidates. As already proposed by Daetwyler et al. [39] and Brard and Ricard [40], another approach would be to reverse the prediction equations without availability of genotyping data for computing $Me$. Required reliabilities and other parameters should be obtained from a reference population and different generations of selection candidates in which genomic prediction is already applied. However, estimates of $Me$ obtained by the reversion of prediction equations would be underestimates, since this would include a correction for the fact that the error variance decreases when multiple loci are used, which is trait-dependent.

This study has introduced the concept of the effective number of chromosome segments originating from a specific breed ($b$), and shared by selection candidates ($S$) from this breed and crossbred reference animals ($Rc$), $Me_{S,Rc}^{\left( b \right)}$. This $Me_{S,Rc}^{\left( b \right)}$ is different from $Me_{S,Rc}$ as defined previously, since the latter does not take the breed origin of the chromosome segments of the crossbred animals into consideration. Indeed, each purebred population has its own value of $Me$, while the genome of crossbred animals combines segments from the different populations they originated from. Thus, the value of $Me_{S,Rc}$ includes both the effective number of chromosome segments segregating in breed $b$, and the effective number of chromosome segments segregating in the other breed(s) of origin for the crossbred animals, while $Me_{S,Rc}^{\left( b \right)}$ only involves the effective number of chromosome segments segregating in breed $b$. For this study, it was assumed that $Me_{S,Rc}^{\left( b \right)}$ (i.e., $Me_{a,AB}^{\left( A \right)}$) was equal to $Me_{S,R}$ (i.e., $Me_{a,A}$) for which the breed $b$ selection candidates and reference animals ($R$) share the same parents as the crossbred $Rc$ animals. This assumption was valid based on the results obtained. In practice, such an assumption would not be possible, since the purebred and crossbred reference animals may not share the same parents, or reference animals may belong to different generations. Further research on accurate estimation of $Me$ is therefore required.

## Conclusions

Several equations for predicting the reliability of genomic EBV for crossbred performance based on ASGM or on BSAM were derived for three different scenarios. These three scenarios involved a reference population that included only purebred animals, only crossbred animals, or both. The prediction equations were derived for application either without or with availability of genotyping data. Results showed that the reliabilities predicted without availability of genotyping data were of the same order of magnitude as the predictions of reliabilities predicted with availability of genotyping data. Thus, the proposed equations applied either without or with availability of genotyping data can be used to evaluate the effects of several parameters on the reliability of genomic EBV for crossbred performance (e.g., the genetic correlation between purebred and crossbred performances, heritabilities of the traits, number of reference animals, distance between breeds), and for the optimization of the design of breeding programs. Moreover, we showed that model BSAM can outperform model ASGM for a breed, if the effective number of chromosome segments originating from this breed and shared by selection candidates of this breed and crossbred reference animals is less than half the effective number of all chromosome segments that are independently segregating in these same animals, provided all other parameters remain equal. It is necessary to improve estimation of the effective number of chromosome segments to predict the reliability of genomic EBV without availability of genotyping data more accurately.

## Additional files

**Additional file 1.** Derivation of the equation for predicting the reliability of genomic estimated breeding values without availability of data. Based on the mixed model theory, a derivation of the equation for predicting the reliability of genomic estimated breeding values is detailed in this document, assuming that effects of all independent loci are estimated simultaneously, and assuming a single population.

**Additional file 2.** Derivation of the prediction equation without availability of data, using a breed-specific allele substitution effects model and only crossbred reference animals. A derivation of the equation that predicts the reliability of genomic estimated breeding values for crossbred performance using a breed-specific allele substitution effects model and only crossbred animals without availability of genotyping data is detailed in this document.

**Additional file 3.** Derivation of the prediction equation without availability of data, using a breed-specific allele substitution effects model, and purebred and crossbred reference animals. A derivation of the equation for predicting the reliability of genomic estimated breeding values for crossbred performance using a breed-specific allele substitution effects model and a reference population including both purebred and crossbred animals, without availability of genotyping data, is detailed in this document.

**Additional file 4: Table S1.** Reliabilities for crossbred performance with a purebred reference population and a genetic correlation equal to 0.3. **Table S2.** Reliabilities for crossbred performance with a purebred reference population and a genetic correlation equal to 0.7. **Table S3.** Reliabilities for crossbred performance with a crossbred reference population originated from two related breeds. **Table S4.** Reliabilities for crossbred performance with a crossbred reference population originated from two unrelated breeds. **Table S5.** Reliabilities for crossbred performance with a mixed reference population assuming two related breeds and a genetic correlation of 0.3. **Table S6.** Reliabilities for crossbred performance with a mixed reference population assuming two related breeds and a genetic correlation of 0.7. **Table S7.** Reliabilities for crossbred performance with a mixed reference population assuming two unrelated breeds and a genetic correlation of 0.3. **Table S8.** Reliabilities for crossbred performance with a mixed reference population assuming two unrelated breeds and a genetic correlation of 0.7.
