# Supplementary material for: Prediction of the reliability of genomic breeding values for crossbred performance
Source: Genet Sel Evol. 2017 May 12;49:43. doi: 10.1186/s12711-017-0318-1 (PMC5439167; doi:10.1186/s12711-017-0318-1)
Supplement: Supplementary file 1 — Additional file 1. Derivation of the equation for predicting the reliability of genomic estimated breeding values without availability of data. Based on the mixed model theory, a derivation of the equation for predicting the reliability of genomic estimated breeding values is detailed in this document, assuming that effects of all independent loci are estimated simultaneously, and assuming a single population. [file 12711_2017_318_MOESM1_ESM.pdf]

Additional file 1: Derivation of the equation for predicting the reliability of genomic estimated breeding values without availability of data.

Based on the mixed model theory, a derivation of the equation for predicting the reliability of genomic estimated breeding values is detailed hereafter, assuming that effects of all independent loci are estimated simultaneously, and assuming a single population. Consider N unrelated reference animals genotyped for Me independent loci and associated with one record. It is assumed that the effect β_k^* of each kth independent locus explains an equal amount of the additive genetic variance σ_a^2, i.e. $\sigma_a^2 = Me\sigma_{\beta^*}^2$ with $\sigma_{\beta^*}^2$ being the variance at one locus. It is also assumed that reliability of the estimated effect is the same for each locus ($r_{\beta^*}^2$). The matrix $\mathbf{Z}^*$ contains the standardized genotypes as $\mathbf{Z}_{lk}^* = \frac{\mathbf{M}_{lk}-2p_k}{\sqrt{2p_k(1-p_k)}}$ with $\mathbf{M}_{ik}$ being the genotype (coded as 0 for homozygous genotypes, 1 for heterozygous genotypes, or 2 for alternate homozygous genotypes) of the lth individual for the kth locus, and p_k being the allele frequency of the kth locus.

The genomic breeding value (a_i) for the ith selection candidate can be predicted as:

$$\hat{\mathrm{a}}_i = \mathbf{z}_i^* \widehat{\boldsymbol{\beta}^*},$$

where $\mathbf{z}_\mathrm{i}'$ is a row vector for the Me independent loci of the ith selection candidate, and the vector $\widehat{\boldsymbol{\beta}^*}$ is the vector of the predictions of $\boldsymbol{\beta}^*$.

Following the mixed model theory [17, 19], the reliability of $\hat{\mathrm{a}}_i$, $r_{\mathrm{a}_i}^2$, is equal to:

$$r_{\mathrm{a}_i}^2 = 1 - \frac{Var(\hat{\mathrm{a}}_i - \mathrm{a}_i)}{Var(\mathrm{a}_i)} = \frac{Var(\hat{\mathrm{a}}_i)}{Var(\mathrm{a}_i)} = \frac{Var\left(\mathbf{z}_i^* \widehat{\boldsymbol{\beta}^*}\right)}{Var(\mathbf{z}_i^* \boldsymbol{\beta}^*)}$$

$$= \frac{\mathbf{z}_i^* Var(\widehat{\boldsymbol{\beta}^*})\mathbf{z}_i^{*'}}{\mathbf{z}_i^* Var(\boldsymbol{\beta}^*)\mathbf{z}_i^{*'}} = \frac{Var(\widehat{\beta_k^*})}{Var(\beta_k^*)}$$

$= \frac{Var(\beta_k^*) - Var(\beta_k^* - \widehat{\beta_k^*})}{Var(\beta_k^*)} = r_{\beta^*}^2,$

because it was assumed that the effect β_k^* of each kth independent locus explains an equal amount of the additive genetic variance, and that the reliability of the predicted effect, $r_{\beta^*}^2$, is the same for each locus.

The reliability $r_{\beta^*}^2$ can be approximated as follows. The prediction of β_k^* for the kth locus can be performed from the phenotypes, $\mathbf{y}$, corrected for all other fixed and random effects (e.g., $\widehat{\boldsymbol{\beta}_{\neq k}^*}$), $\hat{\mathbf{y}}$, using the model:

$\hat{\mathbf{y}} = \mathbf{y} - \mathbf{Z}_{\neq k}^* \widehat{\boldsymbol{\beta}_{\neq k}^*} =$

$\mathbf{z}_k^* \beta_k^* + \mathbf{Z}_{\neq k}^* \boldsymbol{\beta}_{\neq k}^* + \mathbf{e} - \mathbf{Z}_{\neq k}^* \widehat{\boldsymbol{\beta}_{\neq k}^*} = \mathbf{z}_k^* \beta_k^* + \boldsymbol{\varepsilon}_k,$

with $\mathbf{Z}^* = [\mathbf{z}_k^* \quad \mathbf{Z}_{\neq k}^*]$, $\boldsymbol{\beta}' = [\beta_k^* \quad \boldsymbol{\beta}_{\neq k}^{*\prime}]$, and $\boldsymbol{\varepsilon}_k$ is a residual vector.

It follows that $\boldsymbol{\varepsilon}_k = \mathbf{Z}_{\neq k}^* \boldsymbol{\beta}_{\neq k}^* - \mathbf{Z}_{\neq k}^* \widehat{\boldsymbol{\beta}_{\neq k}^*} + \mathbf{e}$. The variance of $\mathbf{y}$ is equal to $Var(\mathbf{y}) = Var(\mathbf{z}_k^* \beta_k^* + \mathbf{Z}_{\neq k}^* \boldsymbol{\beta}_{\neq k}^* + \mathbf{e}) = \mathbf{z}_k^* \mathbf{z}_k^{*\prime} \sigma_{\beta^*}^2 + Var(\mathbf{Z}_{\neq k}^* \boldsymbol{\beta}_{\neq k}^*) + Var(\mathbf{e})$**,** and similarly, the variance of $\hat{\mathbf{y}}$ is equal to $Var(\hat{\mathbf{y}}) = \mathbf{z}_k^* \mathbf{z}_k^{*\prime} \sigma_{\beta^*}^2 + Var(\boldsymbol{\varepsilon}_k)$. The variance of $\boldsymbol{\varepsilon}_k$ is unknown and can be derived as follows:

$Var(\boldsymbol{\varepsilon}_k) = Var\left(\mathbf{Z}_{\neq k}^* \boldsymbol{\beta}_{\neq k}^* - \mathbf{Z}_{\neq k}^* \widehat{\boldsymbol{\beta}_{\neq k}^*} + \mathbf{e}\right)$

$= \mathbf{Z}_{\neq k}^* \mathbf{Z}_{\neq k}^{*\prime} Var\left(\boldsymbol{\beta}_{\neq k}^* - \widehat{\boldsymbol{\beta}_{\neq k}^*}\right) + Var(\mathbf{e})$

$= \mathbf{Z}_{\neq k}^* \mathbf{Z}_{\neq k}^{*\prime} \sigma_{\beta^*}^2 \left(1 - r_{\beta^*}^2\right) + \mathbf{I}\sigma_e^2$

$\approx \mathbf{I}\left(\sigma_a^2 \left(1 - r_{\beta^*}^2\right) + \sigma_e^2\right) = \mathbf{I}\sigma_\varepsilon^2,$

where σ_e^2 is the residual variance; null covariances between random effects are assumed due to independent loci; and $Cov(\mathbf{Z}_{\neq k}^{*}\widehat{\boldsymbol{\beta}_{\neq k}^{*}}, \mathbf{e}) = \mathbf{0}$.

The approximation $\mathbf{Z}_{\neq k}^{*}\mathbf{Z}_{\neq k}^{*\prime}\sigma_{\beta^*}^2 \approx \mathbf{I}\sigma_a^2$ is performed because unrelated animals were assumed and because a single independent locus explains a small amount of σ_a^2.

It is worth noting that $\sigma_\varepsilon^2 = \sigma_a^2(1 - r_{\beta^*}^2) + \sigma_e^2$ is equivalent to the correction developed by Daetwyler et al. [12] in their Appendix, assuming that the phenotypic variance $\sigma_P^2 = 1$.

Therefore, the prediction of β_k^*, $\widehat{\beta_k^*}$, is equal to, following the mixed model theory [17]:

$$\widehat{\beta_k^*} = \left(\mathbf{z}_k^{*\prime}\mathbf{z}_k^{*}\sigma_\varepsilon^{-2} + \sigma_{\beta^*}^{-2}\right)^{-1}\sigma_\varepsilon^{-2}\mathbf{z}_k^{*\prime}\hat{\mathbf{y}},$$

and, the reliability of $\widehat{\beta_k^*}$ is equal to:

$$r_{\beta^*}^2 = \frac{Var(\beta_k^*) - Var\left(\widehat{\beta_k^*} - \beta_k^*\right)}{Var(\beta_k^*)}$$

$$= \frac{\sigma_{\beta^*}^2 - \left(\mathbf{z}_k^{*\prime}\mathbf{z}_k^{*}\sigma_\varepsilon^{-2} + \sigma_{\beta^*}^{-2}\right)^{-1}}{\sigma_{\beta^*}^2}$$

$$= \frac{\sigma_{\beta^*}^2 - \left(S_{zz,k}\sigma_\varepsilon^{-2} + \sigma_{\beta^*}^{-2}\right)^{-1}}{\sigma_{\beta^*}^2}$$

$$= \frac{\sigma_{\beta^*}^2 - \left(N\sigma_\varepsilon^{-2} + \sigma_{\beta^*}^{-2}\right)^{-1}}{\sigma_{\beta^*}^2}$$

$$= \frac{\left(N\sigma_\varepsilon^{-2} + \sigma_{\beta^*}^{-2}\right)\sigma_{\beta^*}^2 - 1}{\left(N\sigma_\varepsilon^{-2} + \sigma_{\beta^*}^{-2}\right)\sigma_{\beta^*}^2} = \frac{N\sigma_{\beta^*}^2}{N\sigma_{\beta^*}^2 + \sigma_\varepsilon^2},$$

with $S_{zz,k} = N$ being the adjusted sum of squares for the kth locus [12, 14].

Because $r_{a_i}^2 = r_{\beta^*}^2$, $\sigma_a^2 = M_e\sigma_{\beta^*}^2$, and $\sigma_\varepsilon^2 = \left(\sigma_a^2\left(1 - r_{\beta^*}^2\right) + \sigma_e^2\right)$, the reliability of $\hat{a}_i$, $r_{a_i}^2$, is equal to:

$$r_{a_i}^2 = r_{\beta^*}^2 = \frac{N\sigma_{\beta^*}^2}{N\sigma_{\beta^*}^2 + \sigma_\varepsilon^2}$$

$$= \frac{N\sigma_a^2}{N\sigma_a^2 + Me\left(\sigma_a^2\left(1 - r_{a_i}^2\right) + \sigma_e^2\right)} = \frac{Nh_a^2}{Nh_a^2 + Me\left(1 - h_a^2 r_{a_i}^2\right)}.$$

The equation for $r_{a_i}^2 = \frac{Nh_a^2}{Nh_a^2 + Me\left(1 - h_a^2 r_{a_i}^2\right)}$ is equivalent to the equation developed by Daetwyler et al. [12] in the Appendix of their paper. However, similarly to these authors, and because h^2 is considered as small for most traits of interest, the prediction equation reported in the main text of Daetwyler et al. [12] will be used in the main text of this manuscript, i.e. $r_{a_i}^2 = \frac{Nh_a^2}{Nh_a^2 + Me}$. As explained by Daetwyler et al. [12], this approximation has the consequence that the predicted reliabilities are slightly underestimated.
