# Supplementary material for: Prediction of the reliability of genomic breeding values for crossbred performance
Source: Genet Sel Evol. 2017 May 12;49:43. doi: 10.1186/s12711-017-0318-1 (PMC5439167; doi:10.1186/s12711-017-0318-1)
Supplement: Supplementary file 2 — Additional file 2. Derivation of the prediction equation without availability of data, using a breed-specific allele substitution effects model and only crossbred reference animals. A derivation of the equation that predicts the reliability of genomic estimated breeding values for crossbred performance using a breed-specific allele substitution effects model and only crossbred animals without availability of genotyping data is detailed in this document. [file 12711_2017_318_MOESM2_ESM.pdf]

Additional file 2: Derivation of the prediction equation without availability of data, using a breed-specific allele substitution effects model and only crossbred reference animals.

The derivation of the equation that predicts the reliability of genomic estimated breeding values for crossbred performance using a breed-specific allele substitution effects model and only crossbred animals without availability of genotyping data, $r^2_{C\_BSAM\_without}$, is hereafter detailed. Consider N_{AB} unrelated crossbred AB reference animals. It is assumed that the breed-specific effect $\beta^{*(A)}_{c_k}$ ($\beta^{*(B)}_{c_k}$) of each kth independent locus explains an equal amount of the breed-specific additive genetic variance $\sigma^2_{c_A}$ ($\sigma^2_{c_B}$), i.e. $\sigma^2_{c_A} = Me^{(A)}\sigma^2_{\beta^{*(A)}_c}$ ($\sigma^2_{c_B} = Me^{(B)}\sigma^2_{\beta^{*(B)}_c}$) with $Me^{(A)}$ ($Me^{(B)}$) being the effective number of chromosome segments underlying the crossbred performance trait for the breed A (B). The breed-specific effects $\boldsymbol{\beta}^{*(A)}_c$ and $\boldsymbol{\beta}^{*(B)}_c$ are assumed to be independent, i.e., $Cov\left(\boldsymbol{\beta}^{*(A)}_c, \boldsymbol{\beta}^{*(B)}_c\right) = \mathbf{0}$, and the expectations of $\boldsymbol{\beta}^{*(A)}_c$ and $\boldsymbol{\beta}^{*(B)}_c$ are defined as $E\begin{bmatrix}\boldsymbol{\beta}^{*(A)}_c \\ \boldsymbol{\beta}^{*(B)}_c\end{bmatrix} = \begin{bmatrix}\mathbf{0} \\ \mathbf{0}\end{bmatrix}$. The entries of the matrix $\mathbf{Z}^{*(A)}_{AB}$ are defined as $\mathbf{Z}^{*(A)}_{AB_{lk}} = \frac{\mathbf{M}_{lk} - p_{Ak}}{\sqrt{2p_{Ak}(1-p_{Ak})}}$ where the element $\mathbf{M}_{ik}$ is set to 0 or 1 if the kth locus of the lth individual has the breed A allele 1 or 2, respectively, and p_{Ak} is the breed A specific allele frequency at the kth locus. The matrix $\mathbf{Z}^{*(B)}_{AB}$ is defined similarly.

The genomic breeding value ($c^{(A)}_{a_i}$) for the ith selection candidate of breed A can be predicted as follows:

$$\hat{c}^{(A)}_{a_i} = \mathbf{z}^{*(A)}_{a_i}\,\hat{\boldsymbol{\beta}}^{*(A)}_c,$$

where $\mathbf{z}^{*(A)}_{a_i}$ is a row vector of the standardized genotypes for the $Me^{(A)}$ independent loci of the ith selection candidate of breed A; and the vector $\hat{\boldsymbol{\beta}}^{*(A)}_c$ is the vector of the predictions of

$\boldsymbol{\beta}_c^{*(A)}$.

Following the mixed model theory [17, 19], the reliability of $\hat{c}_{a_i}^{(A)}$, $r^2_{c_{a_i}^{(A)}}$, is equal to:

$$r^2_{c_{a_i}^{(A)}} = \frac{Var\left(\hat{c}_{a_i}^{(A)}\right)}{Var\left(c_{a_i}^{(A)}\right)} = \frac{Var\left(\mathbf{z}_{a_i}^{*(A)}\, \hat{\boldsymbol{\beta}}_c^{*(A)}\right)}{Var\left(\mathbf{z}_{a_i}^{*(A)}\, \boldsymbol{\beta}_c^{*(A)}\right)}$$

$$= \frac{\mathbf{z}_{a_i}^{*(A)} Var\left(\hat{\boldsymbol{\beta}}_c^{*(A)}\right) \mathbf{z}_{a_i}^{*(A)\prime}}{\mathbf{z}_{a_i}^{*(A)} Var\left(\boldsymbol{\beta}_c^{*(A)}\right) \mathbf{z}_{a_i}^{*(A)\prime}} = \frac{Var\left(\hat{\beta}_{c_k}^{*(A)}\right)}{Var\left(\beta_{c_k}^{*(A)}\right)} = r^2_{\beta_c^{*(A)}},$$

because it is assumed that the allele substitution effect $\beta_{c_k}^{*(A)}$ of each kth independent locus explains an equal amount of the breed A specific additive genetic variance $\sigma^2_{c_A}$, and that the accuracy of the estimated effect, $r^2_{\beta_c^{*(A)}}$, is the same for each locus.

The reliability $r^2_{\beta_c^{*(A)}}$ can be approximated as follows. The prediction of $\beta_{c_k}^{*(A)}$ for the kth locus can be performed from the phenotypes, $\mathbf{y}_{AB}$, corrected for all other fixed effects, for the breed A specific effects other than the kth effect, $\hat{\boldsymbol{\beta}}_{c_{\neq k}}^{*(A)}$, as well as for the breed B specific effects, $\hat{\boldsymbol{\beta}}_c^{*(B)}$, $\widehat{\mathbf{y}_{AB}^{*}}$, using the model:

$$\widehat{\mathbf{y}_{AB}^{*}} = \mathbf{y}_{AB} - \mathbf{Z}_{AB_{\neq k}}^{*(A)}\, \hat{\boldsymbol{\beta}}_{c_{\neq k}}^{*(A)} - \mathbf{Z}_{AB}^{*(B)} \hat{\boldsymbol{\beta}}_c^{*(B)}$$

$$= \mathbf{z}_{AB_k}^{*(A)}\, \beta_{c_k}^{*(A)} + \mathbf{Z}_{AB_{\neq k}}^{*(A)}\, \boldsymbol{\beta}_{c_{\neq k}}^{*(A)} + \mathbf{Z}_{AB}^{*(B)} \boldsymbol{\beta}_c^{*(B)}$$

$$+\mathbf{e}_{AB} - \mathbf{Z}_{AB_{\neq k}}^{*(A)}\, \hat{\boldsymbol{\beta}}_{c_{\neq k}}^{*(A)} - \mathbf{Z}_{AB}^{*(B)} \hat{\boldsymbol{\beta}}_c^{*(B)} = \mathbf{z}_{AB_k}^{*(A)}\, \beta_{c_k}^{*(A)} + \boldsymbol{\varepsilon}_{AB_k},$$

where $\mathbf{Z}_{AB}^{*(A)} = \left[\mathbf{z}_{AB_k}^{*(A)} \quad \mathbf{Z}_{AB_{\neq k}}^{*(A)}\right]$; and $\boldsymbol{\varepsilon}_{AB_k}$ is the residual vector.

The variance of $\mathbf{y}_{AB}$ is equal to:

$$Var(\mathbf{y}_{AB}) = Var\left(\mathbf{z}_{AB_k}^{*(A)}\,\beta_{c_k}^{*(A)} + \mathbf{Z}_{AB_{\neq k}}^{*(A)}\,\boldsymbol{\beta}_{c_{\neq k}}^{*(A)} + \mathbf{Z}_{AB}^{*(B)}\boldsymbol{\beta}_{c}^{*(B)} + \mathbf{e}_{AB}\right)$$

$$= \left(\mathbf{z}_{AB_k}^{*(A)}\mathbf{z}_{AB_k}^{*(A)\prime} + \mathbf{Z}_{AB_{\neq k}}^{*(A)}\mathbf{Z}_{AB_{\neq k}}^{*(A)\prime}\right)\sigma_{\beta_c^{*(A)}}^2 + \mathbf{Z}_{AB}^{*(B)}\mathbf{Z}_{AB}^{*(B)\prime}\sigma_{\beta_c^{*(B)}}^2 + \mathbf{I}\sigma_{e_{AB}}^2,$$

with $\sigma_{e_{AB}}^2$ being the residual variance of the crossbred performance trait, and assuming null covariances among all random effects.

Similarly, the variance of $\widehat{\mathbf{y}_{AB}^{*}}$ is equal to:

$$Var\left(\widehat{\mathbf{y}_{AB}^{*}}\right) = \mathbf{z}_{AB_k}^{*(A)}\mathbf{z}_{AB_k}^{*(A)\prime}\sigma_{\beta_c^{*(A)}}^2 + Var\left(\boldsymbol{\varepsilon}_{AB_k}\right).$$

The unknown variance of $\boldsymbol{\varepsilon}_{AB_k}$, $Var\left(\boldsymbol{\varepsilon}_{AB_k}\right)$, can be derived as follows:

$$Var\left(\boldsymbol{\varepsilon}_{AB_k}\right) = Var\left(\widehat{\mathbf{y}_{AB}^{*}}\right) - \mathbf{z}_{AB_k}^{*(A)}\mathbf{z}_{AB_k}^{*(A)\prime}\sigma_{\beta_c^{*(A)}}^2$$

$$= Var\left(\mathbf{y}_{AB} - \mathbf{Z}_{AB_{\neq k}}^{*(A)}\,\widehat{\boldsymbol{\beta}}_{c_{\neq k}}^{*(A)} - \mathbf{Z}_{AB}^{*(B)}\widehat{\boldsymbol{\beta}}_{c}^{*(B)}\right) - \mathbf{z}_{AB_k}^{*(A)}\mathbf{z}_{AB_k}^{*(A)\prime}\sigma_{\beta_c^{*(A)}}^2$$

$$= Var(\mathbf{y}_{AB}) + Var\left(\mathbf{Z}_{AB_{\neq k}}^{*(A)}\,\widehat{\boldsymbol{\beta}}_{c_{\neq k}}^{*(A)} + \mathbf{Z}_{AB}^{*(B)}\widehat{\boldsymbol{\beta}}_{c}^{*(B)}\right)$$

$$-Cov\left(\mathbf{y}_{AB}, \mathbf{Z}_{AB_{\neq k}}^{*(A)}\,\widehat{\boldsymbol{\beta}}_{c_{\neq k}}^{*(A)} + \mathbf{Z}_{AB}^{*(B)}\widehat{\boldsymbol{\beta}}_{c}^{*(B)}\right)$$

$$-Cov\left(\mathbf{Z}_{AB_{\neq k}}^{*(A)}\,\widehat{\boldsymbol{\beta}}_{c_{\neq k}}^{*(A)} + \mathbf{Z}_{AB}^{*(B)}\widehat{\boldsymbol{\beta}}_{c}^{*(B)}, \mathbf{y}_{AB}\right) - \mathbf{z}_{AB_k}^{*(A)}\mathbf{z}_{AB_k}^{*(A)\prime}\sigma_{\beta_c^{*(A)}}^2$$

$$= Var(\mathbf{y}_{AB}) + Var\left(\mathbf{Z}_{AB_{\neq k}}^{*(A)}\,\widehat{\boldsymbol{\beta}}_{c_{\neq k}}^{*(A)} + \mathbf{Z}_{AB}^{*(B)}\widehat{\boldsymbol{\beta}}_{c}^{*(B)}\right)$$

$$-2Cov\begin{pmatrix} \mathbf{z}_{AB_k}^{*(A)}\,\beta_{c_k}^{*(A)} + \mathbf{Z}_{AB_{\neq k}}^{*(A)}\,\boldsymbol{\beta}_{c_{\neq k}}^{*(A)} + \mathbf{Z}_{AB}^{*(B)}\boldsymbol{\beta}_{c}^{*(B)} \\ +\mathbf{e}_{AB}, \mathbf{Z}_{AB_{\neq k}}^{*(A)}\,\widehat{\boldsymbol{\beta}}_{c_{\neq k}}^{*(A)} + \mathbf{Z}_{AB}^{*(B)}\widehat{\boldsymbol{\beta}}_{c}^{*(B)} \end{pmatrix} - \mathbf{z}_{AB_k}^{*(A)}\mathbf{z}_{AB_k}^{*(A)\prime}\sigma_{\beta_c^{*(A)}}^2$$

$$= Var(\mathbf{y}_{AB}) - Var\left(\mathbf{Z}_{AB_{\neq k}}^{*(A)}\,\widehat{\boldsymbol{\beta}}_{c_{\neq k}}^{*(A)} + \mathbf{Z}_{AB}^{*(B)}\widehat{\boldsymbol{\beta}}_{c}^{*(B)}\right) - \mathbf{z}_{AB_k}^{*(A)}\mathbf{z}_{AB_k}^{*(A)\prime}\sigma_{\beta_c^{*(A)}}^2$$

$$= \left(\mathbf{z}_{AB_k}^{*(A)}\mathbf{z}_{AB_k}^{*(A)\prime} + \mathbf{Z}_{AB_{\neq k}}^{*(A)}\mathbf{Z}_{AB_{\neq k}}^{*(A)\prime}\right)\sigma_{\beta_c^{*(A)}}^2$$

$$+\mathbf{Z}_{AB}^{*(B)}\mathbf{Z}_{AB}^{*(B)\prime}\sigma_{\beta_c^{*(B)}}^2 + \mathbf{I}\sigma_{e_{AB}}^2 - Var\left(\mathbf{Z}_{AB_{\neq k}}^{*(A)}\,\widehat{\boldsymbol{\beta}}_{c_{\neq k}}^{*(A)}\right)$$

$$-Var\left(\mathbf{Z}_{AB}^{*(B)}\widehat{\boldsymbol{\beta}}_{c}^{*(B)}\right) - \mathbf{z}_{AB_k}^{*(A)}\mathbf{z}_{AB_k}^{*(A)\prime}\sigma_{\beta_c^{*(A)}}^2$$

$$= \mathbf{Z}_{AB_{\neq k}}^{*(A)}\mathbf{Z}_{AB_{\neq k}}^{*(A)\prime}\sigma_{\beta_c^{*(A)}}^2\left(1 - r_{\beta_c^{*(A)}}^2\right) + \mathbf{Z}_{AB}^{*(B)}\mathbf{Z}_{AB}^{*(B)\prime}\sigma_{\beta_c^{*(B)}}^2\left(1 - r_{\beta_c^{*(B)}}^2\right) + \mathbf{I}\sigma_{e_{AB}}^2$$

$$\approx \mathbf{I}\left(\frac{\sigma_{c_A}^2}{2}\left(1 - r_{\beta_c^{*(A)}}^2\right) + \frac{\sigma_{c_B}^2}{2}\left(1 - r_{\beta_c^{*(B)}}^2\right) + \sigma_{e_{AB}}^2\right)$$

$$\approx \mathbf{I}\left(\sigma_{P_{AB}}^2 - \frac{\sigma_{c_A}^2}{2}r_{\beta_c^{*(A)}}^2 - \frac{\sigma_{c_B}^2}{2}r_{\beta_c^{*(B)}}^2\right),$$

where $\sigma_{P_{AB}}^2$ is the phenotypic variance of the crossbred performance trait; $r_{\beta_c^{*(B)}}^2$ is the reliability of the estimated effects $\widehat{\boldsymbol{\beta}}_c^{*(B)}$;

$$Var\left(\mathbf{Z}_{AB_{\neq k}}^{*(A)}\,\widehat{\boldsymbol{\beta}}_{c_{\neq k}}^{*(A)} + \mathbf{Z}_{AB}^{*(B)}\widehat{\boldsymbol{\beta}}_{c}^{*(B)}\right)$$

$$= Cov\left(\mathbf{Z}_{AB_{\neq k}}^{*(A)}\,\boldsymbol{\beta}_{c_{\neq k}}^{*(A)} + \mathbf{Z}_{AB}^{*(B)}\boldsymbol{\beta}_{c}^{*(B)}, \mathbf{Z}_{AB_{\neq k}}^{*(A)}\,\widehat{\boldsymbol{\beta}}_{c_{\neq k}}^{*(A)} + \mathbf{Z}_{AB}^{*(B)}\widehat{\boldsymbol{\beta}}_{c}^{*(B)}\right);$$

$$Cov\left(\mathbf{z}_{AB_k}^{*(A)}\,\beta_{c_k}^{*(A)} + \mathbf{e}_{AB}, \mathbf{Z}_{AB_{\neq k}}^{*(A)}\,\widehat{\boldsymbol{\beta}}_{c_{\neq k}}^{*(A)} + \mathbf{Z}_{AB}^{*(B)}\widehat{\boldsymbol{\beta}}_{c}^{*(B)}\right) = \mathbf{0}.$$

The approximations $\mathbf{Z}_{AB_{\neq k}}^{*(A)}\mathbf{Z}_{AB_{\neq k}}^{*(A)\prime}\sigma_{\beta_c^{*(A)}}^2 \approx \mathbf{I}\frac{\sigma_{c_A}^2}{2}$ and $\mathbf{Z}_{AB}^{*(B)}\mathbf{Z}_{AB}^{*(B)\prime}\sigma_{\beta_c^{*(B)}}^2 \approx \mathbf{I}\frac{\sigma_{c_B}^2}{2}$ are performed because unrelated animals were assumed and because a single independent locus explains a small amount of $\sigma_{c_A}^2$ and $\sigma_{c_B}^2$.

Therefore, following the mixed model theory [17, 19], the prediction of $\beta_{c_k}^{*(A)}$, $\hat{\beta}_{c_k}^{*(A)}$, is equal to:

$$\hat{\beta}_{c_k}^{*(A)} = \left(\mathbf{z}_{AB_k}^{*(A)\prime} \mathbf{z}_{AB_k}^{*(A)} \sigma_{\varepsilon_{AB}}^{-2} + \sigma_{\beta_c^{*(A)}}^{-2} \right)^{-1} \sigma_{\varepsilon_{AB}}^{-2} \mathbf{z}_{AB_k}^{*(A)\prime} \widehat{\mathbf{y}_{AB}^{*}},$$

with $\sigma_{\varepsilon_{AB}}^2 = \sigma_{P_{AB}}^2 - \frac{\sigma_{c_A}^2}{2} r_{\beta_c^{*(A)}}^2 - \frac{\sigma_{c_B}^2}{2} r_{\beta_c^{*(B)}}^2.$

and, the reliability of $\hat{\beta}_{c_k}^{*(A)}$ is equal to:

$$r_{\beta_c^{*(A)}} = \frac{Var\left(\beta_{c_k}^{*(A)}\right) - Var\left(\hat{\beta}_{c_k}^{*(A)} - \beta_{c_k}^{*(A)}\right)}{Var\left(\beta_{c_k}^{*(A)}\right)}$$

$$= \frac{\sigma_{\beta_c^{*(A)}}^2 - \left(\mathbf{z}_{AB_k}^{*(A)\prime} \mathbf{z}_{AB_k}^{*(A)} \sigma_{\varepsilon_{AB}}^{-2} + \sigma_{\beta_c^{*(A)}}^{-2} \right)^{-1}}{\sigma_{\beta_c^{*(A)}}^2}$$

$$= \frac{\sigma_{\beta_c^{*(A)}}^2 - \left(S_{zz,k}^{(A)} \sigma_{\varepsilon_{AB}}^{-2} + \sigma_{\beta_c^{*(A)}}^{-2} \right)^{-1}}{\sigma_{\beta_c^{*(A)}}^2}$$

$$= \frac{\sigma_{\beta_c^{*(A)}}^2 - \left(\frac{N_{AB}}{2} \sigma_{\varepsilon_{AB}}^{-2} + \sigma_{\beta_c^{*(A)}}^{-2} \right)^{-1}}{\sigma_{\beta_c^{*(A)}}^2}$$

$$= \frac{\left(\frac{N_{AB}}{2} \sigma_{\varepsilon_{AB}}^{-2} + \sigma_{\beta_c^{*(A)}}^{-2} \right) \sigma_{\beta_c^{*(A)}}^2 - 1}{\left(\frac{N_{AB}}{2} \sigma_{\varepsilon_{AB}}^{-2} + \sigma_{\beta_c^{*(A)}}^{-2} \right) \sigma_{\beta_c^{*(A)}}^2}$$

$$= \frac{\frac{N_{AB}}{2} \sigma_{\varepsilon_{AB}}^{-2} \sigma_{\beta_c^{*(A)}}^2}{\frac{N_{AB}}{2} \sigma_{\varepsilon_{AB}}^{-2} \sigma_{\beta_c^{*(A)}}^2 + 1} = \frac{N_{AB} \sigma_{\varepsilon_{AB}}^{-2} \sigma_{\beta_c^{*(A)}}^2}{N_{AB} \sigma_{\varepsilon_{AB}}^{-2} \sigma_{\beta_c^{*(A)}}^2 + 2},$$

with $S_{zz,k}^{(A)} = \frac{N_{AB}}{2}$ being the adjusted sum of squares for the kth locus [12, 14].

Because $r^2_{c_{a_i}^{(A)}} = r^2_{\beta_c^{*(A)}}$, $\sigma^2_{\varepsilon_{AB}} = \left(\sigma^2_{P_{AB}} - \frac{\sigma^2_{c_A}}{2} r^2_{\beta_c^{*(A)}} - \frac{\sigma^2_{c_B}}{2} r^2_{\beta_c^{*(B)}}\right)$, and $\sigma^2_{c_A} = Me^{(A)}\sigma^2_{\beta_c^{*(A)}}$, the predicted reliability of the genomic estimated breeding values for the ith selection candidate of breed A, $r^2_{c_{a_i}^{(A)}}$, is equal to:

$$r^2_{c_{a_i}^{(A)}} = \frac{N_{AB}\sigma^{-2}_{\varepsilon_{AB}}\sigma^2_{\beta_c^{*(A)}}}{N_{AB}\sigma^{-2}_{\varepsilon_{AB}}\sigma^2_{\beta_c^{*(A)}} + 2}$$

$$= \frac{N_{AB}\sigma^2_{c_A}}{N_{AB}\sigma^2_{c_A} + 2Me^{(A)}\left(\sigma^2_{P_{AB}} - \frac{\sigma^2_{c_A}}{2} r^2_{\beta_c^{*(A)}} - \frac{\sigma^2_{c_B}}{2} r^2_{\beta_c^{*(B)}}\right)}$$

$$= \frac{N_{AB}h^2_{c_A}}{N_{AB}h^2_{c_A} + 2Me^{(A)}\left(1 - \frac{1}{2}h^2_{c_A} r^2_{c_a^{(A)}} - \frac{1}{2}h^2_{c_B} r^2_{c_a^{(B)}}\right)},$$

where $h^2_{c_A}$ ($h^2_{c_B}$) is the breed A (B) specific heritability of the crossbred performance.

By ignoring the term $\left(-\frac{1}{2}h^2_{c_A} r^2_{c_a^{(A)}} - \frac{1}{2}h^2_{c_B} r^2_{c_a^{(B)}}\right)$ for small $h^2_{c_A}$ and $h^2_{c_B}$, the prediction equation simplifies to:

$$r^2_{C\_BSAM\_without} = \frac{N_{AB}h^2_{c_A}}{N_{AB}h^2_{c_A} + 2Me^{(A)}}.$$
