# Supplementary material for: Prediction of the reliability of genomic breeding values for crossbred performance
Source: Genet Sel Evol. 2017 May 12;49:43. doi: 10.1186/s12711-017-0318-1 (PMC5439167; doi:10.1186/s12711-017-0318-1)
Supplement: Supplementary file 3 — Additional file 3. Derivation of the prediction equation without availability of data, using a breed-specific allele substitution effects model, and purebred and crossbred reference animals. A derivation of the equation for predicting the reliability of genomic estimated breeding values for crossbred performance using a breed-specific allele substitution effects model and a reference population including both purebred and crossbred animals, without availability of genotyping data, is detailed in this document. [file 12711_2017_318_MOESM3_ESM.pdf]

## Additional file 3: Derivation of the prediction equation without availability of data, using a breed-specific allele substitution effects model, and purebred and crossbred reference animals.

The derivation of the equation for predicting the reliability of genomic estimated breeding values for crossbred performance using a breed-specific allele substitution effects model and a reference population including both purebred and crossbred animals (each associated with one record), without availability of genotyping data, is hereafter detailed. Consider $N_A$ unrelated genotyped breed A reference animals and $N_{AB}$ unrelated genotyped crossbred AB reference animals. It is assumed that the breed A (B) specific effect $\beta_{c_k}^{*(A)}$ ($\beta_{c_k}^{*(B)}$) of each $k^{\text{th}}$ independent locus explains an equal amount of the breed A (B) specific additive genetic variance for the crossbred performance trait $\sigma_{c_A}^2$ ($\sigma_{c_B}^2$), i.e. $\sigma_{c_A}^2 = Me^{(A)}\sigma_{\beta_c^{*(A)}}^2$ ($\sigma_{c_B}^2 = Me^{(B)}\sigma_{\beta_c^{*(B)}}^2$) with $Me^{(A)}$ ($Me^{(B)}$) being the effective number of chromosome segments underlying the crossbred performance trait for the breed A (B). It is also assumed that the effect $\beta_{a_k}^{*(A)}$ of each $k^{\text{th}}$ independent locus explains an equal amount of the additive genetic variance for the purebred performance trait $\sigma_{a_A}^2$, i.e. $\sigma_{a_A}^2 = Me\sigma_{\beta_a^{*(A)}}^2$ with $Me$ being the effective number of chromosome segments underlying the purebred performance trait for the breed A. The (co)variances among $\boldsymbol{\beta}_a^{*(A)}$, $\boldsymbol{\beta}_c^{*(A)}$, and $\boldsymbol{\beta}_c^{*(B)}$, are assumed to be:

$$Var\begin{bmatrix}\boldsymbol{\beta}_a^{*(A)}\\ \boldsymbol{\beta}_c^{*(A)}\\ \boldsymbol{\beta}_c^{*(B)}\end{bmatrix} = \begin{bmatrix}\mathbf{I}\sigma_{\beta_a^{*(A)}}^2 & \mathbf{I}\sigma_{\beta_a^{*(A)}\beta_c^{*(A)}} & \mathbf{0}\\ \mathbf{I}\sigma_{\beta_a^{*(A)}\beta_c^{*(A)}} & \mathbf{I}\sigma_{\beta_c^{*(A)}}^2 & \mathbf{0}\\ \mathbf{0} & \mathbf{0} & \mathbf{I}\sigma_{\beta_c^{*(B)}}^2\end{bmatrix}.$$

The entries of the matrix $\mathbf{Z}_A^{*(A)}$, $\mathbf{Z}_{AB}^{*(A)}$, and $\mathbf{Z}_{AB}^{*(B)}$ are defined as previously in Appendices A and B.

The genomic breeding value ($c_{a_i}^{(A)}$) for the $i^{\text{th}}$ purebred selection candidate can be predicted as

follows:

$$\hat{c}_{a_i}^{(A)} = \mathbf{z}_{a_i}^{*(A)}\, \hat{\boldsymbol{\beta}}_c^{*(A)},$$

where $\mathbf{z}_{a_i}^{(A)}$ is a row vector of the standardized genotypes for the $Me^{(A)}$ independent loci of the $i^{\text{th}}$ selection candidate of breed A; and the vector $\hat{\boldsymbol{\beta}}_c^{*(A)}$ is the vector of the predictions of $\boldsymbol{\beta}_c^{*(A)}$.

Following the mixed model theory [17, 19], the reliability of $\hat{c}_{a_i}^{(A)}$, $r^2_{c_{a_i}^{(A)}}$, is equal to:

$$r^2_{c_{a_i}^{(A)}} = \frac{Var\left(\hat{c}_{a_i}^{(A)}\right)}{Var\left(c_{a_i}^{(A)}\right)} = \frac{Var\left(\mathbf{z}_{a_i}^{*(A)}\, \hat{\boldsymbol{\beta}}_c^{*(A)}\right)}{Var\left(\mathbf{z}_{a_i}^{*(A)}\, \boldsymbol{\beta}_c^{*(A)}\right)}$$

$$= \frac{\mathbf{z}_{a_i}^{*(A)} Var\left(\hat{\boldsymbol{\beta}}_c^{*(A)}\right) \mathbf{z}_{a_i}^{*(A)\prime}}{\mathbf{z}_{a_i}^{*(A)} Var\left(\boldsymbol{\beta}_c^{*(A)}\right) \mathbf{z}_{a_i}^{*(A)\prime}} = \frac{Var\left(\hat{\beta}_{c_k}^{*(A)}\right)}{Var\left(\beta_{c_k}^{*(A)}\right)} = r^2_{\beta_c^{*(A)}},$$

because it is assumed that the effect $\beta_{c_k}^{*(A)}$ of each $k^{\text{th}}$ independent locus explains an equal amount of $\sigma^2_{c_A}$, and that the accuracy of the estimated effect, $r^2_{\beta_c^{*(A)}}$, is the same for each locus.

The reliability $r^2_{\beta_c^{*(A)}}$ can be approximated as follows. The prediction of $\beta_{c_k}^{*(A)}$ for the $k^{\text{th}}$ locus can be performed from the phenotypes, $\mathbf{y}_A$ and $\mathbf{y}_{AB}$, corrected for all other fixed effects, for the breed A specific effects other than the $k^{\text{th}}$ effect, $\hat{\boldsymbol{\beta}}_{a_{\neq k}}^{*(A)}$ and $\hat{\boldsymbol{\beta}}_{c_{\neq k}}^{*(A)}$, as well as for the breed B specific effects , $\hat{\boldsymbol{\beta}}_c^{*(B)}$, $\widehat{\mathbf{y}_A^*}$ and $\widehat{\mathbf{y}_{AB}^*}$ respectively, using the model:

$$\begin{bmatrix} \widehat{\mathbf{y}_A^*} \\ \widehat{\mathbf{y}_{AB}^*} \end{bmatrix} = \begin{bmatrix} \mathbf{z}_{A_k}^{*(A)} & \mathbf{0} \\ \mathbf{0} & \mathbf{z}_{AB_k}^{*(A)} \end{bmatrix} \begin{bmatrix} \beta_{a_k}^{*(A)} \\ \beta_{c_k}^{*(A)} \end{bmatrix} + \begin{bmatrix} \boldsymbol{\varepsilon}_{A_k} \\ \boldsymbol{\varepsilon}_{AB_k} \end{bmatrix},$$

where the vectors $\mathbf{z}_{A_k}^{*(A)}$ and $\mathbf{z}_{AB_k}^{*(A)}$ contain the standardized breed A genotypes of breed A reference animals and breed A alleles of crossbred AB reference animals, respectively; and

$\boldsymbol{\varepsilon}_{A_k}$ and $\boldsymbol{\varepsilon}_{AB_k}$ are the residual vectors.

For simplicity, it will be assumed that $Var\left(\begin{bmatrix}\boldsymbol{\varepsilon}_{A_k}\\ \boldsymbol{\varepsilon}_{AB_k}\end{bmatrix}\right)=\begin{bmatrix}\mathbf{I}\sigma^2_{\varepsilon_A} & \mathbf{0}\\ \mathbf{0} & \mathbf{I}\sigma^2_{\varepsilon_{AB}}\end{bmatrix}$. Following the mixed model theory [17,19], the prediction of $\begin{bmatrix}\beta^{*(A)}_{a_k}\\ \beta^{*(A)}_{c_k}\end{bmatrix}$, $\begin{bmatrix}\hat{\beta}^{*(A)}_{a_k}\\ \hat{\beta}^{*(A)}_{c_k}\end{bmatrix}$, is equal to:

$$\begin{bmatrix}\hat{\beta}^{*(A)}_{a_k}\\ \hat{\beta}^{*(A)}_{c_k}\end{bmatrix}=$$

$$\left(\begin{bmatrix}\mathbf{z}^{*(A)\prime}_{A_k} & \mathbf{0}\\ \mathbf{0} & \mathbf{z}^{*(A)\prime}_{AB_k}\end{bmatrix}\begin{bmatrix}\mathbf{I}\sigma^{-2}_{\varepsilon_A} & \mathbf{0}\\ \mathbf{0} & \mathbf{I}\sigma^{-2}_{\varepsilon_{AB}}\end{bmatrix}\begin{bmatrix}\mathbf{z}^{*(A)}_{A_k} & \mathbf{0}\\ \mathbf{0} & \mathbf{z}^{*(A)}_{AB_k}\end{bmatrix}+\right.$$

$$\left.\begin{bmatrix}\sigma^2_{\beta^{*(A)}_a} & \sigma_{\beta^{*(A)}_a\beta^{*(A)}_c}\\ \sigma_{\beta^{*(A)}_a\beta^{*(A)}_c} & \sigma^2_{\beta^{*(A)}_c}\end{bmatrix}^{-1}\right)^{-1}\begin{bmatrix}\mathbf{z}^{*(A)\prime}_{A_k} & \mathbf{0}\\ \mathbf{0} & \mathbf{z}^{*(A)\prime}_{AB_k}\end{bmatrix}\begin{bmatrix}\mathbf{I}\sigma^{-2}_{\varepsilon_A} & \mathbf{0}\\ \mathbf{0} & \mathbf{I}\sigma^{-2}_{\varepsilon_{AB}}\end{bmatrix}\begin{bmatrix}\widehat{\mathbf{y}^*_A}\\ \widehat{\mathbf{y}^*_{AB}}\end{bmatrix}, \qquad \text{(C1)}$$

and, the reliability of $\hat{\beta}^{*(A)}_{c_k}$ is equal to:

$$r^2_{\beta^{*(A)}_c}=\frac{Var\left(\beta^{*(A)}_{c_k}\right)-Var\left(\hat{\beta}^{*(A)}_{c_k}-\beta^{*(A)}_{c_k}\right)}{Var\left(\beta^{*(A)}_{c_k}\right)}=\frac{\sigma^2_{\beta^{*(A)}_c}-PEV_{\hat{\beta}^{*(A)}_{c_k}}}{\sigma^2_{\beta^{*(A)}_c}},$$

where $PEV_{\hat{\beta}^{*(A)}_{c_k}}$ is the prediction error variance of $\hat{\beta}^{*(A)}_{c_k}$ and is equal to the diagonal element of inverse of the left-hand-side of the mixed model equations (C1) [17].

The inverse of left-hand-side of the mixed model equations (C1) can be simplified as:

$$\left(\begin{bmatrix}\mathbf{z}^{*(A)\prime}_{A_k} & \mathbf{0}\\ \mathbf{0} & \mathbf{z}^{*(A)\prime}_{AB_k}\end{bmatrix}\begin{bmatrix}\mathbf{I}\sigma^{-2}_{\varepsilon_A} & \mathbf{0}\\ \mathbf{0} & \mathbf{I}\sigma^{-2}_{\varepsilon_{AB}}\end{bmatrix}\begin{bmatrix}\mathbf{z}^{*(A)}_{A_k} & \mathbf{0}\\ \mathbf{0} & \mathbf{z}^{*(A)}_{AB_k}\end{bmatrix}+\begin{bmatrix}\sigma^2_{\beta^{*(A)}_a} & \sigma_{\beta^{*(A)}_a\beta^{*(A)}_c}\\ \sigma_{\beta^{*(A)}_a\beta^{*(A)}_c} & \sigma^2_{\beta^{*(A)}_c}\end{bmatrix}^{-1}\right)^{-1}$$

$$= \left( \begin{bmatrix} \mathbf{z}_{A_k}^{*(A)\prime}\mathbf{z}_{A_k}^{*(A)}\sigma_{\varepsilon_A}^{-2} & 0 \\ 0 & \mathbf{z}_{AB_k}^{*(A)\prime}\mathbf{z}_{AB_k}^{*(A)}\sigma_{\varepsilon_{AB}}^{-2} \end{bmatrix} \right.$$

$$\left. + \frac{1}{\sigma_{\beta_a^{*(A)}}^2\sigma_{\beta_c^{*(A)}}^2 - \sigma_{\beta_a^{*(A)}\beta_c^{*(A)}}^2} \begin{bmatrix} \sigma_{\beta_c^{*(A)}}^2 & -\sigma_{\beta_a^{*(A)}\beta_c^{*(A)}} \\ -\sigma_{\beta_a^{*(A)}\beta_c^{*(A)}} & \sigma_{\beta_a^{*(A)}}^2 \end{bmatrix} \right)^{-1}$$

$$= \left( \begin{bmatrix} N_A\sigma_{\varepsilon_A}^{-2} & 0 \\ 0 & \frac{N_{AB}}{2}\sigma_{\varepsilon_{AB}}^{-2} \end{bmatrix} + \frac{1}{\sigma_{\beta_a^{*(A)}}^2\sigma_{\beta_c^{*(A)}}^2 - \sigma_{\beta_a^{*(A)}\beta_c^{*(A)}}^2} \begin{bmatrix} \sigma_{\beta_c^{*(A)}}^2 & -\sigma_{\beta_a^{*(A)}\beta_c^{*(A)}} \\ -\sigma_{\beta_a^{*(A)}\beta_c^{*(A)}} & \sigma_{\beta_a^{*(A)}}^2 \end{bmatrix} \right)^{-1}$$

Defining the correlation between $\beta_a^{*(A)}$ and $\beta_c^{*(A)}$ as $r_{\beta_a^{*(A)}\beta_c^{*(A)}} = \frac{\sigma_{\beta_a^{*(A)}\beta_c^{*(A)}}}{\sigma_{\beta_a^{*(A)}}\sigma_{\beta_c^{*(A)}}}$, it follows that:

$\frac{1}{\sigma_{\beta_a^{*(A)}}^2\sigma_{\beta_c^{*(A)}}^2 - \sigma_{\beta_a^{*(A)}}^2\sigma_{\beta_c^{*(A)}}^2 r_{\beta_a^{*(A)}\beta_c^{*(A)}}^2} = \frac{1}{\sigma_{\beta_a^{*(A)}}^2\sigma_{\beta_c^{*(A)}}^2\left(1-r_{\beta_a^{*(A)}\beta_c^{*(A)}}^2\right)} = \frac{1}{\tau}$, and

$$\left( \begin{bmatrix} \mathbf{z}_{A_k}^{*(A)\prime} & \mathbf{0} \\ \mathbf{0} & \mathbf{z}_{AB_k}^{*(A)\prime} \end{bmatrix} \begin{bmatrix} \mathbf{I}\sigma_{\varepsilon_A}^{-2} & \mathbf{0} \\ \mathbf{0} & \mathbf{I}\sigma_{\varepsilon_{AB}}^{-2} \end{bmatrix} \begin{bmatrix} \mathbf{z}_{A_k}^{*(A)} & \mathbf{0} \\ \mathbf{0} & \mathbf{z}_{AB_k}^{*(A)} \end{bmatrix} + \begin{bmatrix} \sigma_{\beta_a^{*(A)}}^2 & \sigma_{\beta_a^{*(A)}\beta_c^{*(A)}} \\ \sigma_{\beta_a^{*(A)}\beta_c^{*(A)}} & \sigma_{\beta_c^{*(A)}}^2 \end{bmatrix}^{-1} \right)^{-1}$$

$$= \tau \left( \begin{bmatrix} \tau N_A\sigma_{\varepsilon_A}^{-2} + \sigma_{\beta_c^{*(A)}}^2 & -\sigma_{\beta_a^{*(A)}\beta_c^{*(A)}} \\ -\sigma_{\beta_a^{*(A)}\beta_c^{*(A)}} & \tau\frac{N_{AB}}{2}\sigma_{\varepsilon_{AB}}^{-2} + \sigma_{\beta_a^{*(A)}}^2 \end{bmatrix} \right)^{-1}$$

$$= \frac{\tau}{\left(\tau N_A\sigma_{\varepsilon_A}^{-2} + \sigma_{\beta_c^{*(A)}}^2\right)\left(\tau\frac{N_{AB}}{2}\sigma_{\varepsilon_{AB}}^{-2} + \sigma_{\beta_a^{*(A)}}^2\right) - \sigma_{\beta_a^{*(A)}\beta_c^{*(A)}}^2} \begin{bmatrix} \tau\frac{N_{AB}}{2}\sigma_{\varepsilon_{AB}}^{-2} + \sigma_{\beta_a^{*(A)}}^2 & \sigma_{\beta_a^{*(A)}\beta_c^{*(A)}} \\ \sigma_{\beta_a^{*(A)}\beta_c^{*(A)}} & \tau N_A\sigma_{\varepsilon_A}^{-2} + \sigma_{\beta_c^{*(A)}}^2 \end{bmatrix}$$

The prediction error variance of $\hat{\beta}_{c_k}^{*(A)}$, $PEV_{\hat{\beta}_{c_k}^{*(A)}}$, is then equal to

$PEV_{\hat{\beta}_{c_k}^{*(A)}} = \frac{\tau\left(\tau N_A\sigma_{\varepsilon_A}^{-2}+\sigma_{\beta_c^{*(A)}}^2\right)}{\left(\tau N_A\sigma_{\varepsilon_A}^{-2}+\sigma_{\beta_c^{*(A)}}^2\right)\left(\tau\frac{N_{AB}}{2}\sigma_{\varepsilon_{AB}}^{-2}+\sigma_{\beta_a^{*(A)}}^2\right)-\sigma_{\beta_a^{*(A)}\beta_c^{*(A)}}^2}$, and the reliability of $\hat{\beta}_{c_k}^{*(A)}$ is equal to:

$$r_{\beta_c^{*(A)}} = \frac{\sigma^2_{\beta_c^{*(A)}} - PEV_{\widehat{\beta}_{c_k}^{*(A)}}}{\sigma^2_{\beta_c^{*(A)}}} = \frac{\sigma^2_{\beta_c^{*(A)}} - \frac{\tau\left(\tau N_A \sigma_{\varepsilon_A}^{-2} + \sigma^2_{\beta_c^{*(A)}}\right)}{\left(\tau N_A \sigma_{\varepsilon_A}^{-2} + \sigma^2_{\beta_c^{*(A)}}\right)\left(\tau \frac{N_{AB}}{2}\sigma_{\varepsilon_{AB}}^{-2} + \sigma^2_{\beta_a^{*(A)}}\right) - \sigma^2_{\beta_a^{*(A)}\beta_c^{*(A)}}}}{\sigma^2_{\beta_c^{*(A)}}}$$

$$= \frac{\left(\tau N_A \sigma_{\varepsilon_A}^{-2} + \sigma^2_{\beta_c^{*(A)}}\right)\left(\tau \frac{N_{AB}}{2}\sigma_{\varepsilon_{AB}}^{-2} + \sigma^2_{\beta_a^{*(A)}}\right)\sigma^2_{\beta_c^{*(A)}} - \sigma^2_{\beta_a^{*(A)}\beta_c^{*(A)}}\sigma^2_{\beta_c^{*(A)}} - \tau\left(\tau N_A \sigma_{\varepsilon_A}^{-2} + \sigma^2_{\beta_c^{*(A)}}\right)}{\left(\tau N_A \sigma_{\varepsilon_A}^{-2} + \sigma^2_{\beta_c^{*(A)}}\right)\left(\tau \frac{N_{AB}}{2}\sigma_{\varepsilon_{AB}}^{-2} + \sigma^2_{\beta_a^{*(A)}}\right)\sigma^2_{\beta_c^{*(A)}} - \sigma^2_{\beta_a^{*(A)}\beta_c^{*(A)}}\sigma^2_{\beta_c^{*(A)}}}$$

$$= \frac{\left(\tau\sigma_{\varepsilon_A}^{-2} + \frac{1}{N_A}\sigma^2_{\beta_c^{*(A)}}\right)\left(\frac{1}{2}\tau\sigma_{\varepsilon_{AB}}^{-2} + \frac{1}{N_{AB}}\sigma^2_{\beta_a^{*(A)}}\right) - \frac{1}{N_A}\frac{1}{N_{AB}}\sigma^2_{\beta_a^{*(A)}\beta_c^{*(A)}} - \frac{1}{N_{AB}}\tau\sigma^{-2}_{\beta_c^{*(A)}}\left(\tau\sigma_{\varepsilon_A}^{-2} + \frac{1}{N_A}\sigma^2_{\beta_c^{*(A)}}\right)}{\left(\tau\sigma_{\varepsilon_A}^{-2} + \frac{1}{N_A}\sigma^2_{\beta_c^{*(A)}}\right)\left(\frac{1}{2}\tau\sigma_{\varepsilon_{AB}}^{-2} + \frac{1}{N_{AB}}\sigma^2_{\beta_a^{*(A)}}\right) - \frac{1}{N_A}\frac{1}{N_{AB}}\sigma^2_{\beta_a^{*(A)}\beta_c^{*(A)}}}$$

$$= \frac{\left(\tau\sigma_{\varepsilon_A}^{-2} + \frac{1}{N_A}\sigma^2_{\beta_c^{*(A)}}\right)\left(\frac{1}{2}\tau\sigma_{\varepsilon_{AB}}^{-2} + \frac{1}{N_{AB}}\sigma^2_{\beta_a^{*(A)}}\right) - \frac{1}{N_A}\frac{1}{N_{AB}}\sigma^2_{\beta_a^{*(A)}\beta_c^{*(A)}} - \frac{1}{N_{AB}}\tau\sigma^{-2}_{\beta_c^{*(A)}}\left(\tau\sigma_{\varepsilon_A}^{-2} + \frac{1}{N_A}\sigma^2_{\beta_c^{*(A)}}\right)}{\frac{1}{2}\tau\tau\sigma_{\varepsilon_A}^{-2}\sigma_{\varepsilon_{AB}}^{-2} + \frac{1}{N_{AB}}\tau\sigma_{\varepsilon_A}^{-2}\sigma^2_{\beta_a^{*(A)}} + \frac{1}{2}\frac{1}{N_A}\tau\sigma^2_{\beta_c^{*(A)}}\sigma_{\varepsilon_{AB}}^{-2} + \frac{1}{N_A}\frac{1}{N_{AB}}\left(\sigma^2_{\beta_c^{*(A)}}\sigma^2_{\beta_a^{*(A)}} - \sigma^2_{\beta_a^{*(A)}\beta_c^{*(A)}}\right)}$$

$$= \frac{\frac{1}{2}\tau\tau\sigma_{\varepsilon_A}^{-2}\sigma_{\varepsilon_{AB}}^{-2} + \frac{1}{N_{AB}}\tau\sigma_{\varepsilon_A}^{-2}\sigma^2_{\beta_a^{*(A)}} + \frac{1}{2}\frac{1}{N_A}\tau\sigma^2_{\beta_c^{*(A)}}\sigma_{\varepsilon_{AB}}^{-2} + \frac{1}{N_A}\frac{1}{N_{AB}}\tau - \frac{1}{N_{AB}}\tau\sigma^{-2}_{\beta_c^{*(A)}}\left(\tau\sigma_{\varepsilon_A}^{-2} + \frac{1}{N_A}\sigma^2_{\beta_c^{*(A)}}\right)}{\frac{1}{2}\tau\tau\sigma_{\varepsilon_A}^{-2}\sigma_{\varepsilon_{AB}}^{-2} + \frac{1}{N_{AB}}\tau\sigma_{\varepsilon_A}^{-2}\sigma^2_{\beta_a^{*(A)}} + \frac{1}{2}\frac{1}{N_A}\tau\sigma^2_{\beta_c^{*(A)}}\sigma_{\varepsilon_{AB}}^{-2} + \frac{1}{N_A}\frac{1}{N_{AB}}\tau}$$

$$= \frac{\frac{1}{2}\sigma^2_{\beta_a^{*(A)}}\sigma^2_{\beta_c^{*(A)}}\sigma_{\varepsilon_A}^{-2}\sigma_{\varepsilon_{AB}}^{-2} - \frac{1}{2}\sigma^2_{\beta_a^{*(A)}}\sigma^2_{\beta_c^{*(A)}}\sigma_{\varepsilon_A}^{-2}\sigma_{\varepsilon_{AB}}^{-2}r^2_{\beta_a^{*(A)}\beta_c^{*(A)}} + \frac{1}{N_{AB}}\sigma^2_{\beta_a^{*(A)}}\sigma_{\varepsilon_A}^{-2} + \frac{1}{2}\frac{1}{N_A}\sigma^2_{\beta_c^{*(A)}}\sigma_{\varepsilon_{AB}}^{-2} + \frac{1}{N_A}\frac{1}{N_{AB}} - \frac{1}{N_{AB}}\sigma^{-2}_{\beta_c^{*(A)}}\left(\tau\sigma_{\varepsilon_A}^{-2} + \frac{1}{N_A}\sigma^2_{\beta_c^{*(A)}}\right)}{\frac{1}{2}\sigma^2_{\beta_a^{*(A)}}\sigma^2_{\beta_c^{*(A)}}\sigma_{\varepsilon_A}^{-2}\sigma_{\varepsilon_{AB}}^{-2} - \frac{1}{2}\sigma^2_{\beta_a^{*(A)}}\sigma^2_{\beta_c^{*(A)}}\sigma_{\varepsilon_A}^{-2}\sigma_{\varepsilon_{AB}}^{-2}r^2_{\beta_a^{*(A)}\beta_c^{*(A)}} + \frac{1}{N_{AB}}\sigma^2_{\beta_a^{*(A)}}\sigma_{\varepsilon_A}^{-2} + \frac{1}{2}\frac{1}{N_A}\sigma^2_{\beta_c^{*(A)}}\sigma_{\varepsilon_{AB}}^{-2} + \frac{1}{N_A}\frac{1}{N_{AB}}}$$

$$= \frac{\frac{1}{2}\sigma^2_{\beta_a^{*(A)}}\sigma^2_{\beta_c^{*(A)}}\sigma_{\varepsilon_A}^{-2}\sigma_{\varepsilon_{AB}}^{-2} - \frac{1}{2}\sigma^2_{\beta_a^{*(A)}}\sigma^2_{\beta_c^{*(A)}}\sigma_{\varepsilon_A}^{-2}\sigma_{\varepsilon_{AB}}^{-2}r^2_{\beta_a^{*(A)}\beta_c^{*(A)}} + \frac{1}{N_{AB}}\sigma^2_{\beta_a^{*(A)}}\sigma_{\varepsilon_A}^{-2} + \frac{1}{2}\frac{1}{N_A}\sigma^2_{\beta_c^{*(A)}}\sigma_{\varepsilon_{AB}}^{-2} - \frac{1}{N_{AB}}\tau\sigma^{-2}_{\beta_c^{*(A)}}\sigma_{\varepsilon_A}^{-2}}{\frac{1}{2}\sigma^2_{\beta_a^{*(A)}}\sigma^2_{\beta_c^{*(A)}}\sigma_{\varepsilon_A}^{-2}\sigma_{\varepsilon_{AB}}^{-2} - \frac{1}{2}\sigma^2_{\beta_a^{*(A)}}\sigma^2_{\beta_c^{*(A)}}\sigma_{\varepsilon_A}^{-2}\sigma_{\varepsilon_{AB}}^{-2}r^2_{\beta_a^{*(A)}\beta_c^{*(A)}} + \frac{1}{N_{AB}}\sigma^2_{\beta_a^{*(A)}}\sigma_{\varepsilon_A}^{-2} + \frac{1}{2}\frac{1}{N_A}\sigma^2_{\beta_c^{*(A)}}\sigma_{\varepsilon_{AB}}^{-2} + \frac{1}{N_A}\frac{1}{N_{AB}}}$$

$$= \frac{\frac{1}{2}\sigma^2_{\beta_a^{*(A)}}\sigma^2_{\beta_c^{*(A)}}\sigma_{\varepsilon_A}^{-2}\sigma_{\varepsilon_{AB}}^{-2} - \frac{1}{2}\sigma^2_{\beta_a^{*(A)}}\sigma^2_{\beta_c^{*(A)}}\sigma_{\varepsilon_A}^{-2}\sigma_{\varepsilon_{AB}}^{-2}r^2_{\beta_a^{*(A)}\beta_c^{*(A)}} + \frac{1}{N_{AB}}\sigma^2_{\beta_a^{*(A)}}\sigma_{\varepsilon_A}^{-2} + \frac{1}{2}\frac{1}{N_A}\sigma^2_{\beta_c^{*(A)}}\sigma_{\varepsilon_{AB}}^{-2} - \frac{1}{N_{AB}}\left(1 - r^2_{\beta_a^{*(A)}\beta_c^{*(A)}}\right)\sigma^2_{\beta_a^{*(A)}}\sigma_{\varepsilon_A}^{-2}}{\frac{1}{2}\sigma^2_{\beta_a^{*(A)}}\sigma^2_{\beta_c^{*(A)}}\sigma_{\varepsilon_A}^{-2}\sigma_{\varepsilon_{AB}}^{-2} - \frac{1}{2}\sigma^2_{\beta_a^{*(A)}}\sigma^2_{\beta_c^{*(A)}}\sigma_{\varepsilon_A}^{-2}\sigma_{\varepsilon_{AB}}^{-2}r^2_{\beta_a^{*(A)}\beta_c^{*(A)}} + \frac{1}{N_{AB}}\sigma^2_{\beta_a^{*(A)}}\sigma_{\varepsilon_A}^{-2} + \frac{1}{2}\frac{1}{N_A}\sigma^2_{\beta_c^{*(A)}}\sigma_{\varepsilon_{AB}}^{-2} + \frac{1}{N_A}\frac{1}{N_{AB}}}$$

$$= \frac{\frac{1}{2}\sigma^2_{\beta_a^{*(A)}}\sigma^2_{\beta_c^{*(A)}}\sigma_{\varepsilon_A}^{-2}\sigma_{\varepsilon_{AB}}^{-2} - \frac{1}{2}\sigma^2_{\beta_a^{*(A)}}\sigma^2_{\beta_c^{*(A)}}\sigma_{\varepsilon_A}^{-2}\sigma_{\varepsilon_{AB}}^{-2}r^2_{\beta_a^{*(A)}\beta_c^{*(A)}} + \frac{1}{2}\frac{1}{N_A}\sigma^2_{\beta_c^{*(A)}}\sigma_{\varepsilon_{AB}}^{-2} + \frac{1}{N_{AB}}\sigma^2_{\beta_a^{*(A)}}\sigma_{\varepsilon_A}^{-2}r^2_{\beta_a^{*(A)}\beta_c^{*(A)}}}{\left(\sigma^2_{\beta_a^{*(A)}}\sigma_{\varepsilon_A}^{-2} + \frac{1}{N_A}\right)\left(\frac{1}{2}\sigma^2_{\beta_c^{*(A)}}\sigma_{\varepsilon_{AB}}^{-2} + \frac{1}{N_{AB}}\right) - \frac{1}{2}\sigma^2_{\beta_a^{*(A)}}\sigma^2_{\beta_c^{*(A)}}\sigma_{\varepsilon_A}^{-2}\sigma_{\varepsilon_{AB}}^{-2}r^2_{\beta_a^{*(A)}\beta_c^{*(A)}}}$$

$$
= \frac{\frac{1}{2}\sigma^2_{\beta_c^{*(A)}}\sigma^{-2}_{\varepsilon_{AB}}\left(\sigma^2_{\beta_a^{*(A)}}\sigma^{-2}_{\varepsilon_A}+\frac{1}{N_A}\right)+\sigma^2_{\beta_a^{*(A)}}\sigma^{-2}_{\varepsilon_A}r^2_{\beta_a^{*(A)}\beta_c^{*(A)}}\left(\frac{1}{2}\sigma^2_{\beta_c^{*(A)}}\sigma^{-2}_{\varepsilon_{AB}}+\frac{1}{N_{AB}}\right)-\sigma^2_{\beta_a^{*(A)}}\sigma^2_{\beta_c^{*(A)}}\sigma^{-2}_{\varepsilon_A}\sigma^{-2}_{\varepsilon_{AB}}r^2_{\beta_a^{*(A)}\beta_c^{*(A)}}}{\left(\sigma^2_{\beta_a^{*(A)}}\sigma^{-2}_{\varepsilon_A}+\frac{1}{N_A}\right)\left(\frac{1}{2}\sigma^2_{\beta_c^{*(A)}}\sigma^{-2}_{\varepsilon_{AB}}+\frac{1}{N_{AB}}\right)-\frac{1}{2}\sigma^2_{\beta_a^{*(A)}}\sigma^2_{\beta_c^{*(A)}}\sigma^{-2}_{\varepsilon_A}\sigma^{-2}_{\varepsilon_{AB}}r^2_{\beta_a^{*(A)}\beta_c^{*(A)}}}
$$

It is worth noting that the denominator:

$$
\left(\sigma^2_{\beta_a^{*(A)}}\sigma^{-2}_{\varepsilon_A}+\frac{1}{N_A}\right)\left(\frac{1}{2}\sigma^2_{\beta_c^{*(A)}}\sigma^{-2}_{\varepsilon_{AB}}+\frac{1}{N_{AB}}\right)-\frac{1}{2}\sigma^2_{\beta_a^{*(A)}}\sigma^2_{\beta_c^{*(A)}}\sigma^{-2}_{\varepsilon_A}\sigma^{-2}_{\varepsilon_{AB}}r^2_{\beta_a^{*(A)}\beta_c^{*(A)}}
$$

is the determinant of the 2-by-2 matrix:

$$
\begin{bmatrix} \sigma^2_{\beta_a^{*(A)}}\sigma^{-2}_{\varepsilon_A}+\frac{1}{N_A} & \sqrt{\frac{1}{2}\sigma^2_{\beta_a^{*(A)}}\sigma^2_{\beta_c^{*(A)}}\sigma^{-2}_{\varepsilon_A}\sigma^{-2}_{\varepsilon_{AB}}r^2_{\beta_a^{*(A)}\beta_c^{*(A)}}} \\ \sqrt{\frac{1}{2}\sigma^2_{\beta_a^{*(A)}}\sigma^2_{\beta_c^{*(A)}}\sigma^{-2}_{\varepsilon_A}\sigma^{-2}_{\varepsilon_{AB}}r^2_{\beta_a^{*(A)}\beta_c^{*(A)}}} & \frac{1}{2}\sigma^2_{\beta_c^{*(A)}}\sigma^{-2}_{\varepsilon_{AB}}+\frac{1}{N_{AB}} \end{bmatrix}.
$$

Therefore, after reordering the terms, it follows that:

$$
r_{\beta_c^{*(A)}} =
$$

$$
\begin{bmatrix} \sqrt{\frac{\sigma^2_{\beta_a^{*(A)}}r^2_{\beta_a^{*(A)}\beta_c^{*(A)}}}{\sigma^2_{\varepsilon_A}}} \\ \sqrt{\frac{\sigma^2_{\beta_c^{*(A)}}}{2\sigma^2_{\varepsilon_{AB}}}} \end{bmatrix}' \begin{bmatrix} \frac{\sigma^2_{\beta_a^{*(A)}}}{\sigma^2_{\varepsilon_A}}+\frac{1}{N_A} & \sqrt{\frac{\sigma^2_{\beta_a^{*(A)}}\sigma^2_{\beta_c^{*(A)}}r^2_{\beta_a^{*(A)}\beta_c^{*(A)}}}{2\sigma^2_{\varepsilon_A}\sigma^2_{\varepsilon_{AB}}}} \\ \sqrt{\frac{\sigma^2_{\beta_a^{*(A)}}\sigma^2_{\beta_c^{*(A)}}r^2_{\beta_a^{*(A)}\beta_c^{*(A)}}}{2\sigma^2_{\varepsilon_A}\sigma^2_{\varepsilon_{AB}}}} & \frac{\sigma^2_{\beta_c^{*(A)}}}{2\sigma^2_{\varepsilon_{AB}}}+\frac{1}{N_{AB}} \end{bmatrix}^{-1} \begin{bmatrix} \sqrt{\frac{\sigma^2_{\beta_a^{*(A)}}r^2_{\beta_a^{*(A)}\beta_c^{*(A)}}}{\sigma^2_{\varepsilon_A}}} \\ \sqrt{\frac{\sigma^2_{\beta_c^{*(A)}}}{2\sigma^2_{\varepsilon_{AB}}}} \end{bmatrix}.
$$

By approximating the residual variances $\sigma^2_{\varepsilon_A}$ and $\sigma^2_{\varepsilon_{AB}}$ by the corresponding $\sigma^2_{P_A}$ and $\sigma^2_{P_{AB}}$, and because $r^2_{c^{(A)}_{a_i}} = r^2_{\beta_c^{*(A)}}$, $\sigma^2_{c_A} = Me^{(A)}\sigma^2_{\beta_c^{*(A)}}$, $\sigma^2_{a_A} = Me\sigma^2_{\beta_a^{*(A)}}$, and, following Wientjes et al. [14], $r_{\beta_a^{*(A)}\beta_c^{*(A)}} = \frac{\sigma_{\beta_a^{*(A)}\beta_c^{*(A)}}}{\sigma_{\beta_a^{*(A)}}\,\sigma_{\beta_c^{*(A)}}} = r_{PC}^{(A)}$ with $r_{PC}^{(A)}$ being the breed A specific genetic correlation between the purebred and crossbred performance traits, the predicted reliability of the genomic estimated breeding values for the $i^{\text{th}}$ selection candidate of breed A is equal to, without availability of genotyping data:

$$r^2_{c^{(A)}_{a_i}} = r^2_{\beta^{*(A)}_c} = \begin{bmatrix} r^{(A)}_{PC}\sqrt{\frac{h^2_{a_A}}{Me}} & \sqrt{\frac{h^2_{c_A}}{2Me^{(A)}}} \end{bmatrix} \begin{bmatrix} \frac{h^2_{a_A}}{Me} + \frac{1}{N_A} & r^{(A)}_{PC}\sqrt{\frac{h^2_{c_A}}{2Me^{(A)}}\frac{h^2_{a_A}}{Me}} \\ r^{(A)}_{PC}\sqrt{\frac{h^2_{c_A}}{2Me^{(A)}}\frac{h^2_{a_A}}{Me}} & \frac{h^2_{c_A}}{2Me^{(A)}} + \frac{1}{N_{AB}} \end{bmatrix}^{-1} \begin{bmatrix} r^{(A)}_{PC}\sqrt{\frac{h^2_{a_A}}{Me}} \\ \sqrt{\frac{h^2_{c_A}}{2Me^{(A)}}} \end{bmatrix},$$

where $h^2_{a_A}$ is the heritability of the purebred performance trait for the breed A and $h^2_{c_A}$ is the breed A specific heritability for the crossbred performance trait.
