# Supplementary material for: Prediction of the reliability of genomic breeding values for crossbred performance
Source: Genet Sel Evol. 2017 May 12;49:43. doi: 10.1186/s12711-017-0318-1 (PMC5439167; doi:10.1186/s12711-017-0318-1)
Supplement: Supplementary file 4 — Additional file 4: Table S1. Reliabilities for crossbred performance with a purebred reference population and a genetic correlation equal to 0.3. Table S2. Reliabilities for crossbred performance with a purebred reference population and a genetic correlation equal to 0.7. Table S3. Reliabilities for crossbred performance with a crossbred reference population originated from two related breeds. Table S4. Reliabilities for crossbred performance with a crossbred reference population originated from two unrelated breeds. Table S5. Reliabilities for crossbred performance with a mixed reference population assuming two related breeds and a genetic correlation of 0.3. Table S6. Reliabilities for crossbred performance with a mixed reference population assuming two related breeds and a genetic correlation of 0.7. Table S7. Reliabilities for crossbred performance with a mixed reference population assuming two unrelated breeds and a genetic correlation of 0.3. Table S8. Reliabilities for crossbred performance with a mixed reference population assuming two unrelated breeds and a genetic correlation of 0.7. [file 12711_2017_318_MOESM4_ESM.pdf]

## Additional file 4

Table S1. Reliabilities of genomic breeding values for crossbred performance with a purebred reference population and a genetic correlation equal to 0.3. Reliabilities were computed with (With) and without (Without) availability of genotyping data, using a reference population with 2000 or 4000 breed A animals, and separated from the breed A selection candidates by 1 (G1), 2 (G2), or 3 (G3) generation(s). Results are averages (SD) across replicates.

| Heritability | Number of animals | Method | Related breeds | | | Unrelated breeds | | |
|---|---|---|---|---|---|---|---|---|
| | | | G1 | G2 | G3 | G1 | G2 | G3 |
| 0.20 | 2000 | With | 0.014 (0.000) | 0.011 (0.000) | 0.009 (0.000) | 0.015 (0.000) | 0.012 (0.000) | 0.011 (0.000) |
| | | Without | 0.009 (0.000) | 0.008 (0.000) | 0.008 (0.000) | 0.010 (0.000) | 0.010 (0.000) | 0.010 (0.000) |
| | 4000 | With | 0.023 (0.000) | 0.018 (0.000) | 0.015 (0.000) | 0.025 (0.000) | 0.020 (0.000) | 0.018 (0.000) |
| | | Without | 0.016 (0.000) | 0.016 (0.000) | 0.015 (0.000) | 0.018 (0.000) | 0.018 (0.000) | 0.017 (0.000) |
| 0.40 | 2000 | With | 0.028 (0.000) | 0.022 (0.000) | 0.019 (0.000) | 0.030 (0.000) | 0.025 (0.000) | 0.022 (0.000) |
| | | Before | 0.016 (0.000) | 0.016 (0.000) | 0.015 (0.000) | 0.018 (0.000) | 0.018 (0.000) | 0.017 (0.000) |
| | 4000 | With | 0.042 (0.000) | 0.034 (0.000) | 0.030 (0.000) | 0.045 (0.000) | 0.038 (0.000) | 0.034 (0.000) |
| | | Without | 0.027 (0.000) | 0.026 (0.000) | 0.026 (0.000) | 0.030 (0.000) | 0.030 (0.000) | 0.029 (0.000) |
| 0.95 | 2000 | With | 0.051 (0.000) | 0.042 (0.000) | 0.037 (0.000) | 0.055 (0.000) | 0.047 (0.000) | 0.043 (0.000) |
| | | Without | 0.030 (0.000) | 0.030 (0.000) | 0.029 (0.000) | 0.034 (0.000) | 0.033 (0.000) | 0.033 (0.000) |
| | 4000 | With | 0.072 (0.000) | 0.062 (0.000) | 0.057 (0.000) | 0.075 (0.000) | 0.067 (0.000) | 0.063 (0.000) |
| | | Without | 0.045 (0.000) | 0.045 (0.000) | 0.044 (0.000) | 0.049 (0.000) | 0.048 (0.000) | 0.048 (0.000) |

Table S2. Reliabilities of genomic breeding values for crossbred performance with a purebred reference population and a genetic correlation equal to 0.7. Reliabilities were computed with (With) and without (Without) availability of genotyping data, using a reference population with 2000 or 4000 breed A animals, and separated from the breed A selection candidates by 1 (G1), 2 (G2), or 3 (G3) generation(s). Results are averages (SD) across replicates.

| Heritability | Number of animals | Method | Related breeds | | | Unrelated breeds | | |
|---|---|---|---|---|---|---|---|---|
| | | | G1 | G2 | G3 | G1 | G2 | G3 |
| 0.20 | 2000 | With | 0.074 (0.001) | 0.058 (0.000) | 0.049 (0.000) | 0.082 (0.001) | 0.066 (0.001) | 0.058 (0.000) |
| | | Without | 0.047 (0.000) | 0.046 (0.000) | 0.045 (0.000) | 0.055 (0.000) | 0.054 (0.000) | 0.052 (0.000) |
| | 4000 | With | 0.123 (0.000) | 0.098 (0.000) | 0.083 (0.000) | 0.135 (0.000) | 0.111 (0.001) | 0.097 (0.000) |
| | | Without | 0.086 (0.001) | 0.084 (0.001) | 0.082 (0.000) | 0.099 (0.001) | 0.097 (0.001) | 0.094 (0.001) |
| 0.40 | 2000 | With | 0.152 (0.001) | 0.121 (0.001) | 0.104 (0.001) | 0.166 (0.001) | 0.137 (0.001) | 0.121 (0.001) |
| | | Without | 0.086 (0.001) | 0.084 (0.001) | 0.082 (0.001) | 0.099 (0.001) | 0.097 (0.001) | 0.094 (0.001) |
| | 4000 | With | 0.228 (0.000) | 0.186 (0.000) | 0.163 (0.000) | 0.243 (0.001) | 0.205 (0.001) | 0.184 (0.001) |
| | | Without | 0.147 (0.001) | 0.144 (0.001) | 0.141 (0.001) | 0.164 (0.001) | 0.161 (0.001) | 0.158 (0.001) |
| 0.95 | 2000 | With | 0.278 (0.001) | 0.230 (0.001) | 0.204 (0.001) | 0.300 (0.002) | 0.258 (0.002) | 0.234 (0.001) |
| | | Without | 0.165 (0.001) | 0.162 (0.001) | 0.159 (0.001) | 0.183 (0.001) | 0.181 (0.001) | 0.177 (0.001) |
| | 4000 | With | 0.390 (0.000) | 0.339 (0.001) | 0.309 (0.001) | 0.409 (0.000) | 0.366 (0.001) | 0.342 (0.001) |
| | | Without | 0.247 (0.001) | 0.244 (0.001) | 0.240 (0.001) | 0.267 (0.001) | 0.264 (0.001) | 0.260 (0.001) |

Table S3. Reliabilities of genomic breeding values for crossbred performance with a crossbred reference population originated from two related breeds. Reliabilities were computed with (With) and without (Without) availability of genotyping data, based on an across-breed SNP genotype model (ASGM) or on a breed-specific allele substitution effects model (BSAM), and using a reference population with 2000 or 4000 crossbred AB animals. Reference animals were separated from the breed A selection candidates by 1 (G1), 2 (G2), or 3 (G3) generation(s). Results are averages (SD) across replicates.

| Heritability | Generation | Model | 2000 animals | | 4000 animals | |
|---|---|---|---|---|---|---|
| | | | With | Without | With | Without |
| 0.20 | G1 | ASGM | 0.090 (0.001) | 0.081 (0.001) | 0.157 (0.001) | 0.150 (0.001) |
| | | BSAM | 0.064 (0.001) | 0.051 (0.000) | 0.112 (0.001) | 0.097 (0.001) |
| | G2 | ASGM | 0.081 (0.000) | 0.079 (0.000) | 0.141 (0.001) | 0.146 (0.001) |
| | | BSAM | 0.054 (0.000) | 0.049 (0.000) | 0.096 (0.001) | 0.094 (0.001) |
| | G3 | ASGM | 0.075 (0.000) | 0.076 (0.000) | 0.132 (0.001) | 0.142 (0.001) |
| | | BSAM | 0.048 (0.000) | 0.048 (0.000) | 0.087 (0.000) | 0.091 (0.001) |
| 0.40 | G1 | ASGM | 0.195 (0.001) | 0.150 (0.001) | 0.309 (0.001) | 0.261 (0.001) |
| | | BSAM | 0.139 (0.001) | 0.097 (0.001) | 0.228 (0.001) | 0.176 (0.001) |
| | G2 | ASGM | 0.176 (0.001) | 0.146 (0.001) | 0.284 (0.001) | 0.255 (0.001) |
| | | BSAM | 0.120 (0.001) | 0.094 (0.001) | 0.199 (0.001) | 0.172 (0.001) |
| | G3 | ASGM | 0.165 (0.001) | 0.142 (0.001) | 0.268 (0.001) | 0.249 (0.001) |
| | | BSAM | 0.108 (0.001) | 0.091 (0.001) | 0.182 (0.001) | 0.168 (0.001) |
| 0.95 | G1 | ASGM | 0.376 (0.002) | 0.295 (0.002) | 0.582 (0.001) | 0.456 (0.002) |
| | | BSAM | 0.265 (0.002) | 0.202 (0.001) | 0.428 (0.002) | 0.337 (0.002) |
| | G2 | ASGM | 0.348 (0.001) | 0.289 (0.001) | 0.552 (0.001) | 0.448 (0.002) |
| | | BSAM | 0.234 (0.001) | 0.198 (0.001) | 0.389 (0.001) | 0.331 (0.002) |
| | G3 | ASGM | 0.330 (0.001) | 0.282 (0.001) | 0.532 (0.001) | 0.440 (0.002) |
| | | BSAM | 0.215 (0.001) | 0.193 (0.001) | 0.365 (0.001) | 0.323 (0.002) |

Table S4. Reliabilities of genomic breeding values for crossbred performance with a crossbred reference population originated from two unrelated breeds. Reliabilities were computed with (With) and without (Without) availability of genotyping data, based on an across-breed SNP genotype model (ASGM) or on a breed-specific allele substitution effects model (BSAM), and using a reference population with 2000 or 4000 crossbred AB animals. Reference animals were separated from the breed A selection candidates by 1 (G1), 2 (G2), or 3 (G3) generation(s). Results are averages (SD) across replicates.

| Heritability | Generation | Model | 2000 animals | | 4000 animals | |
|---|---|---|---|---|---|---|
| | | | With | Without | With | Without |
| 0.20 | G1 | ASGM | 0.089 (0.001) | 0.079 (0.001) | 0.154 (0.001) | 0.147 (0.001) |
| | | BSAM | 0.075 (0.001) | 0.059 (0.000) | 0.130 (0.001) | 0.112 (0.001) |
| | G2 | ASGM | 0.080 (0.001) | 0.077 (0.001) | 0.139 (0.001) | 0.144 (0.001) |
| | | BSAM | 0.065 (0.001) | 0.058 (0.001) | 0.114 (0.001) | 0.109 (0.001) |
| | G3 | ASGM | 0.074 (0.000) | 0.075 (0.001) | 0.130 (0.001) | 0.140 (0.001) |
| | | BSAM | 0.059 (0.000) | 0.056 (0.000) | 0.105 (0.001) | 0.106 (0.001) |
| 0.40 | G1 | ASGM | 0.192 (0.001) | 0.147 (0.001) | 0.305 (0.001) | 0.256 (0.002) |
| | | BSAM | 0.162 (0.001) | 0.112 (0.001) | 0.260 (0.001) | 0.201 (0.001) |
| | G2 | ASGM | 0.174 (0.001) | 0.144 (0.001) | 0.281 (0.001) | 0.251 (0.001) |
| | | BSAM | 0.143 (0.001) | 0.109 (0.001) | 0.233 (0.001) | 0.197 (0.002) |
| | G3 | ASGM | 0.163 (0.001) | 0.140 (0.001) | 0.265 (0.001) | 0.246 (0.001) |
| | | BSAM | 0.131 (0.001) | 0.106 (0.001) | 0.216 (0.001) | 0.192 (0.001) |
| 0.95 | G1 | ASGM | 0.374 (0.001) | 0.291 (0.002) | 0.582 (0.001) | 0.450 (0.002) |
| | | BSAM | 0.309 (0.001) | 0.230 (0.002) | 0.489 (0.001) | 0.374 (0.002) |
| | G2 | ASGM | 0.347 (0.001) | 0.285 (0.002) | 0.553 (0.002) | 0.443 (0.002) |
| | | BSAM | 0.279 (0.001) | 0.226 (0.002) | 0.453 (0.002) | 0.368 (0.002) |
| | G3 | ASGM | 0.329 (0.001) | 0.279 (0.002) | 0.534 (0.001) | 0.436 (0.002) |
| | | BSAM | 0.260 (0.001) | 0.220 (0.002) | 0.430 (0.001) | 0.361 (0.002) |

Table S5. Reliabilities of genomic breeding values for crossbred performance with a mixed reference population assuming two related breeds and a genetic correlation of 0.3. Reliabilities were computed with (With) and without (Without) availability of genotyping data, based on an across-breed SNP genotype model (ASGM) or on a breed-specific allele substitution effects model (BSAM). The reference population included 4000 breed A animals, and 2000, or 4000, crossbred AB animals. Reference animals were separated from the breed A selection candidates by 1 (G1), 2 (G2), or 3 (G3) generation(s). Results are averages (SD) across replicates.

| Heritability | Generation | Model | 6000 animals | | 8000 animals | |
|---|---|---|---|---|---|---|
| | | | With | Without | With | Without |
| 0.20 | G1 | ASGM | 0.097 (0.002) | 0.094 (0.001) | 0.153 (0.004) | 0.161 (0.001) |
| | | BSAM | 0.069 (0.003) | 0.065 (0.000) | 0.117 (0.003) | 0.109 (0.001) |
| | G2 | ASGM | 0.089 (0.001) | 0.092 (0.001) | 0.140 (0.003) | 0.157 (0.001) |
| | | BSAM | 0.059 (0.003) | 0.064 (0.000) | 0.101 (0.002) | 0.107 (0.001) |
| | G3 | ASGM | 0.084 (0.000) | 0.089 (0.001) | 0.132 (0.002) | 0.153 (0.001) |
| | | BSAM | 0.053 (0.002) | 0.062 (0.000) | 0.091 (0.002) | 0.104 (0.001) |
| 0.40 | G1 | ASGM | 0.190 (0.007) | 0.169 (0.001) | 0.286 (0.010) | 0.275 (0.001) |
| | | BSAM | 0.149 (0.005) | 0.119 (0.001) | 0.235 (0.004) | 0.194 (0.001) |
| | G2 | ASGM | 0.175 (0.006) | 0.165 (0.001) | 0.266 (0.008) | 0.270 (0.001) |
| | | BSAM | 0.130 (0.004) | 0.116 (0.001) | 0.207 (0.003) | 0.191 (0.001) |
| | G3 | ASGM | 0.165 (0.005) | 0.161 (0.001) | 0.253 (0.007) | 0.264 (0.001) |
| | | BSAM | 0.118 (0.003) | 0.113 (0.001) | 0.190 (0.002) | 0.186 (0.001) |
| 0.95 | G1 | ASGM | 0.354 (0.014) | 0.318 (0.001) | 0.532 (0.017) | 0.469 (0.002) |
| | | BSAM | 0.284 (0.006) | 0.231 (0.001) | 0.440 (0.004) | 0.357 (0.002) |
| | G2 | ASGM | 0.331 (0.012) | 0.312 (0.001) | 0.509 (0.015) | 0.462 (0.002) |
| | | BSAM | 0.253 (0.005) | 0.227 (0.001) | 0.402 (0.003) | 0.351 (0.002) |
| | G3 | ASGM | 0.317 (0.011) | 0.305 (0.001) | 0.493 (0.013) | 0.454 (0.002) |
| | | BSAM | 0.234 (0.005) | 0.222 (0.001) | 0.378 (0.003) | 0.344 (0.002) |

Table S6. Reliabilities of genomic breeding values for crossbred performance with a mixed reference population assuming two related breeds and a genetic correlation of 0.7. Reliabilities were computed with (With) and without (Without) availability of genotyping data, based on an across-breed SNP genotype model (ASGM) or on a breed-specific allele substitution effects model (BSAM). The reference population included 4000 breed A animals, and 2000, or 4000, crossbred AB animals. Reference animals were separated from the breed A selection candidates by 1 (G1), 2 (G2), or 3 (G3) generation(s). Results are averages (SD) across replicates.

| Heritability | Generation | Model | 6000 animals | | 8000 animals | |
|---|---|---|---|---|---|---|
| | | | With | Without | With | Without |
| 0.20 | G1 | ASGM | 0.185 (0.006) | 0.154 (0.001) | 0.236 (0.005) | 0.213 (0.001) |
| | | BSAM | 0.095 (0.017) | 0.129 (0.001) | 0.139 (0.014) | 0.168 (0.001) |
| | G2 | ASGM | 0.175 (0.009) | 0.151 (0.001) | 0.223 (0.007) | 0.208 (0.001) |
| | | BSAM | 0.084 (0.014) | 0.126 (0.001) | 0.122 (0.011) | 0.164 (0.001) |
| | G3 | ASGM | 0.170 (0.011) | 0.147 (0.001) | 0.215 (0.009) | 0.203 (0.001) |
| | | BSAM | 0.076 (0.012) | 0.123 (0.001) | 0.111 (0.010) | 0.160 (0.001) |
| 0.40 | G1 | ASGM | 0.293 (0.007) | 0.258 (0.001) | 0.377 (0.006) | 0.344 (0.002) |
| | | BSAM | 0.195 (0.027) | 0.218 (0.001) | 0.271 (0.019) | 0.278 (0.001) |
| | G2 | ASGM | 0.276 (0.003) | 0.253 (0.001) | 0.356 (0.002) | 0.338 (0.001) |
| | | BSAM | 0.174 (0.022) | 0.214 (0.001) | 0.242 (0.016) | 0.274 (0.001) |
| | G3 | ASGM | 0.265 (0.001) | 0.248 (0.001) | 0.342 (0.001) | 0.331 (0.001) |
| | | BSAM | 0.161 (0.019) | 0.209 (0.001) | 0.224 (0.014) | 0.267 (0.001) |
| 0.95 | G1 | ASGM | 0.481 (0.021) | 0.427 (0.001) | 0.625 (0.015) | 0.538 (0.002) |
| | | BSAM | 0.369 (0.036) | 0.368 (0.002) | 0.500 (0.021) | 0.455 (0.002) |
| | G2 | ASGM | 0.459 (0.017) | 0.421 (0.001) | 0.603 (0.012) | 0.531 (0.002) |
| | | BSAM | 0.339 (0.031) | 0.363 (0.001) | 0.464 (0.018) | 0.450 (0.002) |
| | G3 | ASGM | 0.444 (0.014) | 0.414 (0.001) | 0.588 (0.010) | 0.524 (0.002) |
| | | BSAM | 0.320 (0.028) | 0.357 (0.001) | 0.441 (0.017) | 0.442 (0.002) |

Table S7. Reliabilities of genomic breeding values for crossbred performance with a mixed reference population assuming two unrelated breeds and a genetic correlation of 0.3. Reliabilities were computed with (With) and without (Without) availability of genotyping data, based on an across-breed SNP genotype model (ASGM) or on a breed-specific allele substitution effects model (BSAM). The reference population included 4000 breed A animals, and 2000, or 4000, crossbred AB animals. Reference animals were separated from the breed A selection candidates by 1 (G1), 2 (G2), or 3 (G3) generation(s). Results are averages (SD) across replicates.

| | | | 6000 animals | | 8000 animals | |
|---|---|---|---|---|---|---|
| Heritability | Generation | Model | With | Without | With | Without |
| 0.20 | G1 | ASGM | 0.096 (0.001) | 0.095 (0.001) | 0.147 (0.001) | 0.160 (0.001) |
| | | BSAM | 0.078 (0.001) | 0.075 (0.001) | 0.133 (0.001) | 0.126 (0.001) |
| | G2 | ASGM | 0.089 (0.001) | 0.092 (0.001) | 0.136 (0.001) | 0.157 (0.001) |
| | | BSAM | 0.068 (0.001) | 0.074 (0.001) | 0.117 (0.001) | 0.124 (0.001) |
| | G3 | ASGM | 0.085 (0.000) | 0.090 (0.001) | 0.130 (0.001) | 0.153 (0.001) |
| | | BSAM | 0.062 (0.001) | 0.072 (0.001) | 0.108 (0.001) | 0.120 (0.001) |
| 0.40 | G1 | ASGM | 0.180 (0.002) | 0.169 (0.001) | 0.270 (0.002) | 0.273 (0.002) |
| | | BSAM | 0.168 (0.001) | 0.136 (0.001) | 0.265 (0.001) | 0.221 (0.001) |
| | G2 | ASGM | 0.167 (0.001) | 0.165 (0.001) | 0.252 (0.002) | 0.268 (0.002) |
| | | BSAM | 0.149 (0.001) | 0.133 (0.001) | 0.238 (0.001) | 0.216 (0.002) |
| | G3 | ASGM | 0.160 (0.001) | 0.162 (0.001) | 0.242 (0.002) | 0.262 (0.001) |
| | | BSAM | 0.137 (0.001) | 0.130 (0.001) | 0.221 (0.001) | 0.211 (0.001) |
| 0.95 | G1 | ASGM | 0.332 (0.003) | 0.316 (0.002) | 0.505 (0.004) | 0.465 (0.002) |
| | | BSAM | 0.321 (0.002) | 0.260 (0.002) | 0.497 (0.001) | 0.394 (0.002) |
| | G2 | ASGM | 0.313 (0.002) | 0.310 (0.002) | 0.485 (0.003) | 0.459 (0.002) |
| | | BSAM | 0.292 (0.002) | 0.255 (0.002) | 0.462 (0.002) | 0.388 (0.002) |
| | G3 | ASGM | 0.301 (0.002) | 0.304 (0.001) | 0.472 (0.003) | 0.452 (0.002) |
| | | BSAM | 0.273 (0.001) | 0.250 (0.002) | 0.440 (0.002) | 0.381 (0.002) |

Table S8. Reliabilities of genomic breeding values for crossbred performance with a mixed reference population assuming two unrelated breeds and a genetic correlation of 0.7. Reliabilities were computed with (With) and without (Without) availability of genotyping data, based on an across-breed SNP genotype model (ASGM) or on a breed-specific allele substitution effects model (BSAM). The reference population included 4000 breed A animals, and 2000, or 4000, crossbred AB animals. Reference animals were separated from the breed A selection candidates by 1 (G1), 2 (G2), or 3 (G3) generation(s). Results are averages (SD) across replicates.

| Heritability | Generation | Model | 6000 animals | | 8000 animals | |
|---|---|---|---|---|---|---|
| | | | With | Without | With | Without |
| 0.20 | G1 | ASGM | 0.210 (0.002) | 0.164 (0.001) | 0.257 (0.002) | 0.220 (0.001) |
| | | BSAM | 0.091 (0.003) | 0.147 (0.001) | 0.145 (0.002) | 0.190 (0.001) |
| | G2 | ASGM | 0.203 (0.002) | 0.160 (0.001) | 0.246 (0.002) | 0.215 (0.001) |
| | | BSAM | 0.082 (0.003) | 0.144 (0.001) | 0.130 (0.002) | 0.187 (0.001) |
| | G3 | ASGM | 0.199 (0.002) | 0.156 (0.001) | 0.240 (0.002) | 0.211 (0.001) |
| | | BSAM | 0.076 (0.002) | 0.141 (0.001) | 0.120 (0.002) | 0.182 (0.001) |
| 0.40 | G1 | ASGM | 0.300 (0.001) | 0.269 (0.002) | 0.380 (0.001) | 0.351 (0.002) |
| | | BSAM | 0.193 (0.004) | 0.244 (0.001) | 0.286 (0.003) | 0.309 (0.002) |
| | G2 | ASGM | 0.288 (0.001) | 0.265 (0.001) | 0.363 (0.001) | 0.345 (0.002) |
| | | BSAM | 0.175 (0.004) | 0.240 (0.002) | 0.260 (0.003) | 0.305 (0.002) |
| | G3 | ASGM | 0.280 (0.001) | 0.260 (0.001) | 0.353 (0.001) | 0.339 (0.002) |
| | | BSAM | 0.164 (0.004) | 0.235 (0.001) | 0.244 (0.003) | 0.299 (0.002) |
| 0.95 | G1 | ASGM | 0.467 (0.003) | 0.436 (0.002) | 0.611 (0.003) | 0.542 (0.002) |
| | | BSAM | 0.377 (0.006) | 0.399 (0.002) | 0.537 (0.004) | 0.490 (0.002) |
| | G2 | ASGM | 0.450 (0.003) | 0.431 (0.002) | 0.592 (0.002) | 0.536 (0.002) |
| | | BSAM | 0.350 (0.006) | 0.394 (0.002) | 0.505 (0.004) | 0.485 (0.002) |
| | G3 | ASGM | 0.439 (0.003) | 0.425 (0.002) | 0.580 (0.002) | 0.529 (0.002) |
| | | BSAM | 0.334 (0.006) | 0.388 (0.002) | 0.484 (0.004) | 0.478 (0.002) |
